# Selective CBP/EP300 Bromodomain Inhibitors: Novel Epigenetic Tools to Counter TNF-α-Driven Inflammation

**DOI:** 10.1021/jacsau.5c00085

**Published:** 2025-06-05

**Authors:** Katherine A. Gosselé, Irene Latino, Eleen Laul, Mariia S. Kirillova, Vlad Pascanu, Emanuele Carloni, Rajiv K. Bedi, Chiara Pizzichetti, Amedeo Caflisch, Santiago F. González, Cristina Nevado

**Affiliations:** † Department of Chemistry, 27217 University of Zurich, Zurich CH-8057, Switzerland; ‡ Department of Biochemistry, University of Zurich, Zurich CH-8057, Switzerland; § Faculty of Biomedical Sciences, Università della Svizzera Italiana, Institute for Research in Biomedicine, Bellinzona CH-6500, Switzerland

**Keywords:** CBP/EP300, epigenetics, bromodomain inhibitors, TNF-α, NFκB, inflammation

## Abstract

Tumor necrosis factor α (TNF-α) is a central driver of inflammation in autoimmune conditions such as Crohn’s disease and rheumatoid arthritis (RA). Targeting epigenetic regulators involved in cytokine expression holds therapeutic promise, yet the precise role of the CBP/EP300 bromodomains (BRDs) in modulating immune responses remains poorly understood. Here, we introduce a distinct class of selective CBP/EP300-BRD inhibitors based on a unique 3-methylcinnoline acetyl-lysine mimic, identified through high-throughput fragment docking. These inhibitors significantly reduce TNF-α-driven cytokine expression *in vitro* by blocking NFκB signaling in immune cells. *In vivo*, BRD inhibition led to a robust anti-inflammatory effect, decreasing cytokine secretion (including IL-1β, MCP-1, IL-1α, and IL-6) and preventing immune cell migration to inflamed lymph nodes in a TNF-α-stimulated murine model. Our findings highlight CBP/EP300-BRDs as promising targets for autoimmune therapy, with these non-cytotoxic inhibitors offering a potential complementary approach for RA and other TNF-α-mediated inflammatory conditions.

## Graphical abstract

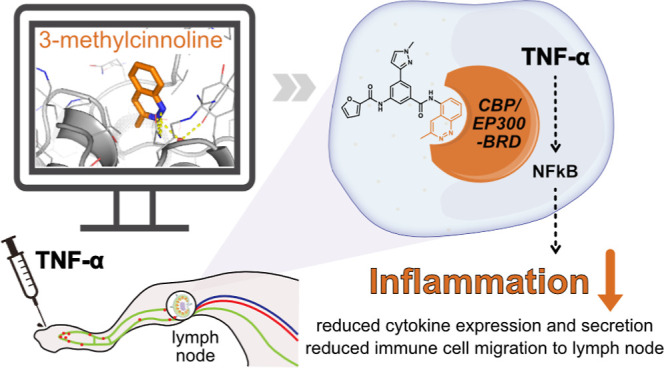

## Introduction

Inflammation is an evolutionary conserved mechanism of the immune system which, among other functions, initiates the host response to pathogens and tissue repair.^1^ Tumor necrosis factor α (TNF-α) is one of the best-characterized mediators of the inflammatory response, playing a key role in autoimmune diseases such as rheumatoid arthritis (RA), psoriasis and Crohn’s disease.^2,3^ Two transmembrane receptors TNFR1 (CD120a) and TNFR2 (CD120b) mediate the mechanism of action of TNF-α by activation of the transcription factor nuclear factor κB (NFκB).^2,4–8^ Recombinant proteins that inhibit TNF-α activity have proved to be effective in treating inflammatory autoimmune diseases, but immunogenicity and supply chain complexity have hampered their broad application.^3,9^ Interestingly, several approaches are now being investigated to block TNF-α using small molecules, none of which have reached the clinic to date.^10,11^ However, epigenetics—known to regulate the signaling pathways downstream of TNF-α^12^—offers an alternative approach to inhibiting TNF-α beyond directly blocking the interaction between TNF-α and TNFR1/2.

The homologous proteins CREB-binding protein (CBP) and E1A-associated protein (EP300) are key epigenetic regulators, able to both “read” and “write” protein lysine acetylation marks through their bromo-(BRD) and histone acetyltransferase (HAT) domains, respectively.^13–17^ One mechanism through which acetylation regulates inflammation is by modulating the transcriptional capacity, DNA-binding ability, and duration of activation of NFκB.^18–20^ In fact, CBP/EP300 are known to acetylate the p65 subunit of NFκB at K310, which is required for NFκB’s full transcriptional activity.^21,22^ Additionally, CBP acts as a co-activator and is essential for NFκB-mediated transcription independently of its HAT activity.^23^

Several inhibitors that target the HAT, BRD and KIX domains of CBP/EP300 have been reported^24–32^ (chemical structures and binding affinities of selected landmark BRD inhibitors in Table S1). Recently, a potent CBP/EP300-HAT inhibitor, C646,^33^ was shown to reduce pro-inflammatory gene expression in LPS-stimulated macrophages.^34,35^ In contrast, the contribution of the CBP/EP300-BRD to inflammation is much less clear—upon treatment with different small molecule BRD inhibitors, both anti- and pro-inflammatory effects have been reported, highlighting the need to characterize better the connection between these two proteins, particularly their BRD, and the inflammatory response.^36–44^ Here, we present the protein structure-based development of a novel, structurally distinct class of CBP/EP300-BRD ligands based on an unprecedented acetyl-lysine mimic—3-methylcinnoline. Our findings reveal a critical role of CBP/EP300-BRD in the TNF-α-induced inflammatory cascade, demonstrating that selective inhibition of this BRD reduces NFκB transcriptional activity, leading to significant downregulation of cytokine gene expression *in vitro*. These effects extend to *in vivo* models, where CBP/EP300-BRD inhibition lowers cytokine secretion levels, and immune cell recruitment within lymphatic tissues in an TNF-α-driven acute inflammation model in mice. This study not only clarifies the role of the CBP/EP300-BRD in inflammation but also highlights its potential as a therapeutic target in treating RA and other TNF-α-mediated inflammatory diseases.

## Results

### Development of CBP/EP300 Inhibitors Bearing a Novel 3-Methylcinnoline Moiety

Our previously reported docking campaign of 419 fragments identified a unique acetylated lysine mimicking scaffold, 3-methylcinnoline, which exhibited the most favorable binding energy when targeting CBP/EP300-BRD.^45^ To validate this fragment, we chimerized a 3-methylcinnoline containing an amino group in 5-position with a 3-acetamido-5-(furancarboxamido) benzoic acid, present in the most potent derivatives of previously in-house developed acetophenone-based CBP inhibitors (Table S1, **16** in Batiste et al.).^32^ This campaign produced our first hit, compound **1** (Figure 1a, for detailed synthetic schemes see Supporting Information). It is important to note that the pose predicted by SEED-docking^46^ was indeed confirmed by the crystal structure of the CBP-BRD in complex with the cinnoline derivative **1** (Figure 1b; PDB code 6SQM).^45^ The conserved polar interactions of acetylated lysines with the side chains of Asn1168 (direct hydrogen bond) and Tyr1125 (water-bridged) were preserved by the two adjacent nitrogens of the cinnoline core. Furthermore, the electrostatic interactions between the furane oxygen and adjacent carbonyl oxygen of ligand **1** and the guanidinium of Arg1173 impart selectivity against the BRD4(1) bromodomain, which has an Asp in the corresponding position in the BC-loop.^32^ Further contribution to the selectivity is due to the difference in a three-residue segment of the ZA-loop (the so-called shelf), namely the triad Leu-Pro-Phe (LPF-shelf, Figure 1b) in CBP/EP300 which is Trp-Pro-Phe in BRD4(1) and the majority of the 61 human bromodomains.^47^

**Figure 1:**
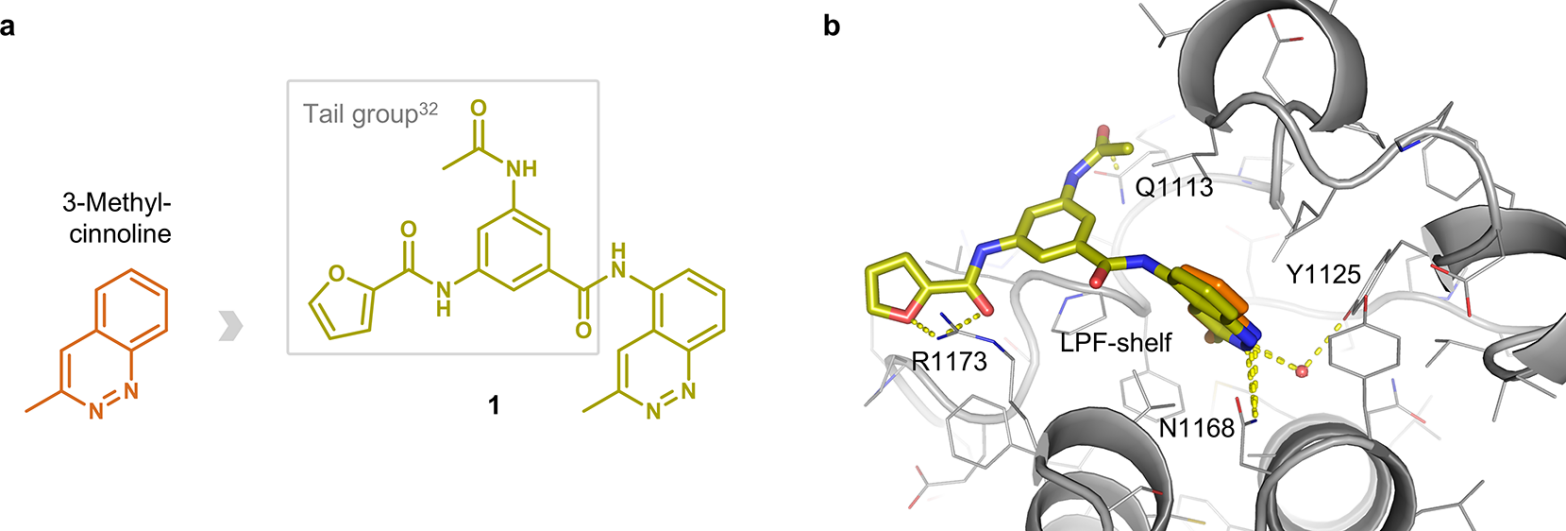
Novel acetyl-lysine mimic 3-methylcinnoline, its derived hit compound **1**, and their binding modes to the CBP-BRD. (a) Chemical structure of **1**—a chimera of 3-methylcinnoline and a tail group of the most potent in house developed acetophenone-based inhibitors.^32^ (b) Overlay of the co-crystal structure of ligand **1** (olive) in the CBP-BRD binding pocket (PDB code: 6SQM)^45^ and the docked 3-methylcinnoline (orange). The key binding interactions are highlighted: direct H-bond interactions with Asn1168 and Arg1173, water mediated interaction with Tyr1125 and stacking interaction with Gln1113. Arg1173 and the Leu-Pro-Phe shelf in CBP/EP300 contribute to the selectivity against BRD4(1).

Although **1** displayed remarkable potency towards CBP and excellent selectivity over BRD4(1), while exhibiting acceptable kinetic solubility (Figure 2a), no ligand engagement *in cellulo* (via InCELL Pulse) could be confirmed (Figure 2b). Similarly, upon treatment of myeloma LP1 cells with **1**, the expected decrease in transcription factor *myc* expression—an established downstream effect of an efficient CBP/EP300-BRD inhibition^48^—was not observed (Figure 2c). This lack of cellular activity is in stark contrast to that of GNE-272 ^25^ (Figure 2b,c), a well-characterized and structurally unrelated CBP/EP300-BRD inhibitor with similar affinity (IC_50_ = 12 nM in-house TR-FRET; Kin. Sol = 75.1 ± 1.0 μM). In fact, the poor cellular activity of **1** could be explained by a bidirectional Caco-2 assay (Table S2), which confirmed a very low membrane permeability for this compound.

**Figure 2:**
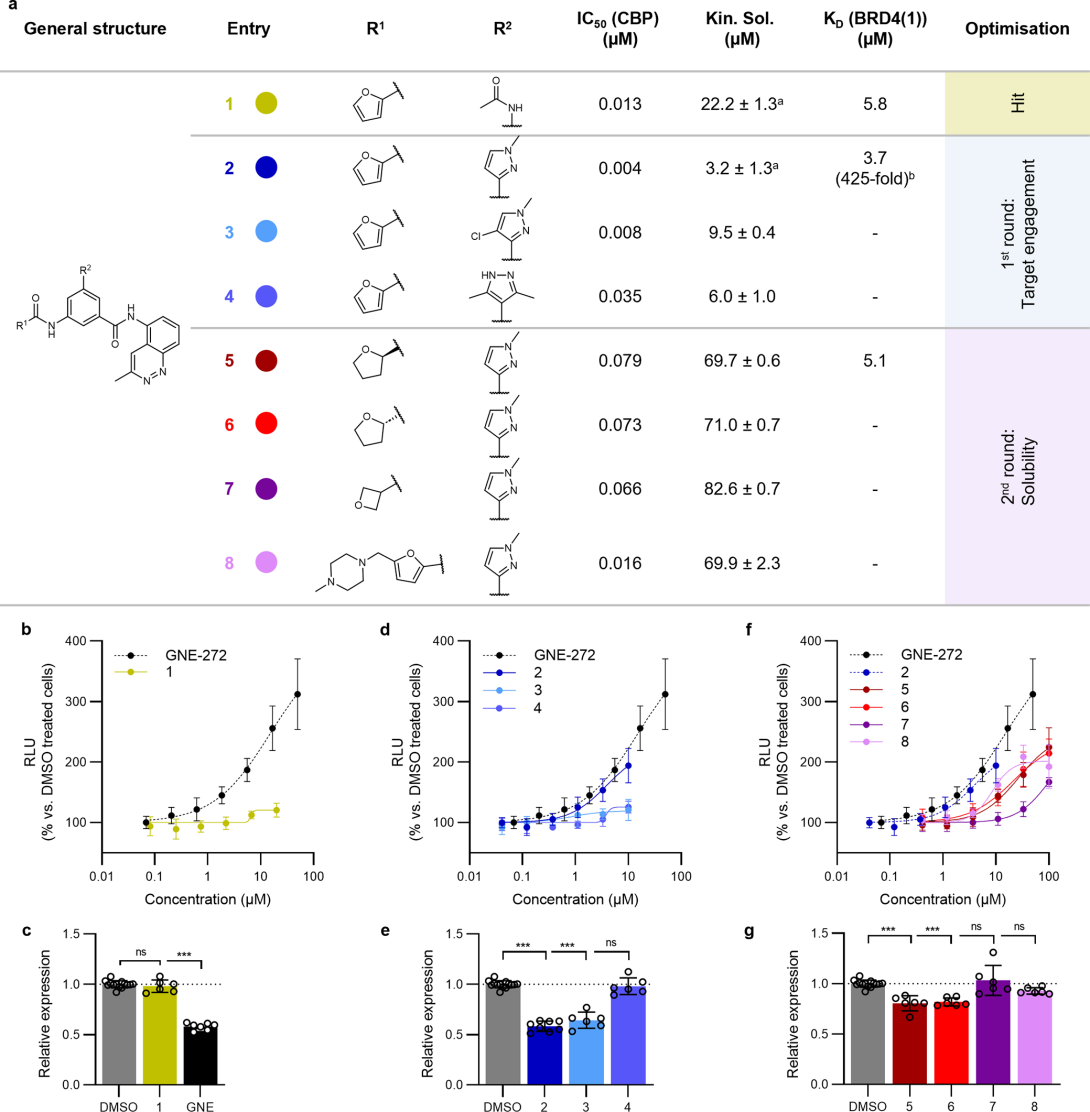
Optimization of cinnoline based CBP/EP300-BRD inhibitors. (a) Structural optimization and biochemical data of the inhibitor series; IC_50_ (CBP) obtained by in-house TR-FRET assay; *K*_D_ (BRD4(1)) and *K*_D_ (CBP) by commercial Bromoscan assay; ± st. dev. between technical replicates, ^a^ *n*=4, all other kinetic solubility measurements *n*=2; ^b^fold-selectivity calculated based on *K*_D_ (CBP) = 0.009 μM. (b,d,f) Cellular binding of developed inhibitors and GNE-272 ^25^ to CBP-BRD as determined by the InCELL Pulse assay following 1 h compound treatment of HEK293T cells transiently transfected with ePL-CBP-BRD; RLU—relative light unit. (c,e,g) *myc* mRNA expression following 4 h treatment of LP1 cells with 1 μM compound; GNE = GNE-272. mRNA expression was quantified by RT-qPCR, and gene expression was normalized to cells treated with DMSO in the same experimental run.

### Hit to Lead Optimization for *In Vitro* and *In Vivo* Studies

Several modifications were designed to improve both the cell permeability and solubility of compound **1**. First, different bioisosters were introduced to replace the acetamide moiety while retaining the stacking interaction with the Gln1113 side chain (Figure 1b). Derivatives bearing 1-methyl- (**2**), 1-methyl-4-chloro- (**3**) and 3,5-dimethyl- (**4**) pyrazole units were synthesized and displayed comparable binding affinities *in vitro* (Figure 2a). Compound **2** showed a significant improvement in terms of cellular target engagement (Figure 2d) and was able to significantly decrease *myc* expression levels (Figure 2e). However, the low kinetic solubility of all three derivatives (Figure 2a) encouraged further modifications targeting saturation at the carboxamide unit.

To address this, (*R*)- and (*S*)-tetrahydrofuran- (**5** and **6**) as well as oxetane-containing (**7**) derivatives were prepared. Further, an *N*-methyl piperazine was added as an ionizable solubilizing group in C5 position of the furane (**8**). As a result, the solubility of these compounds increased drastically by up to 27-fold (Figure 2a). Interestingly, **5** and **6** were able to engage the target very similarly in the InCELL Pulse assay and both significantly decreased the expression level of *myc* (Figure 2f,g). In addition to their excellent binding affinities, **2** and **5** were highly selective over BRD4(1) (Figure 2a), and a broader panel of various BRD-containing proteins (Figure S1), while also exhibiting improved cellular permeability in a Caco-2 assay (Table S2) in comparison to the parent compound **1**. Thus, **2** and **5** were selected for the subsequent biological evaluation of the CBP/EP300-BRD inhibitors in inflammation.

### CBP/EP300-BRD Inhibition Reduces TNF-α-Induced Cytokine Expression by Inhibiting NFκB Signaling

To characterize the anti-inflammatory effect of the CBP/EP300-BRD inhibitors **2** and **5**, we explored their effect on the TNF-α response in THP-1, a human acute monocytic leukemia cell line commonly used to study monocyte and macrophage functions.^49^ Firstly, we validated that stimulation of these cells with 10 ng/mL TNF-α (dose response in Figure S2) increased the expression of several pro-inflammatory cytokines (Figures 3a and S3). Specifically, *il1*β, *il*8 and *tnf*-α all peaked 1 h after TNF-α treatment before reducing again to a level which was elevated in comparison to pre-stimulation. In contrast, *mcp-1* expression displayed different kinetics, continuing to increase for at least 8 h. *il23a* was not strongly induced by TNF-α under these conditions.

**Figure 3:**
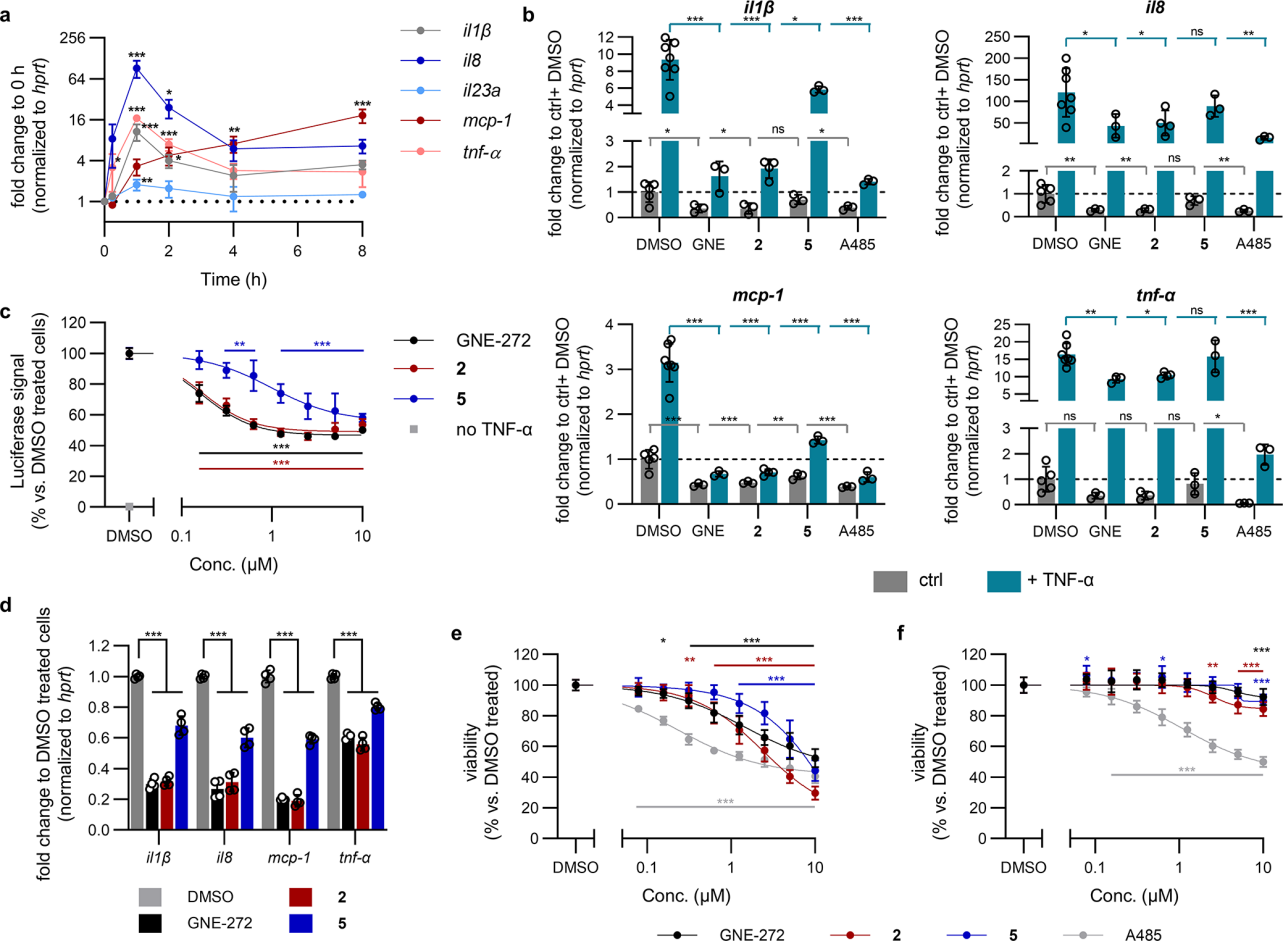
CBP/EP300-BRD inhibitors target TNF-α-induced inflammation *in vitro* with reduced toxicity. (a) Cytokine mRNA expression in THP-1 cells following treatment with 10 ng/mL TNF-α. Gene expression was determined by RT-qPCR and normalized to *hprt* and then to unstimulated cells in the same experimental run. Raw *C*_p_ values shown in Figure S3. (b) Cytokine mRNA expression following 1 h co-treatment of THP-1 cells with 10 ng/mL TNF-α and 1 μM GNE-272 (abbr. GNE), **2**, **5** or A485. mRNA expression quantified by RT-qPCR, normalized to *hprt* and then to the average of ctrl + DMSO treated cells from all experimental runs. Raw *C*_P_ values shown in Figure S4. (c) NFκB-RE luciferase reporter assay. HEK293T cells transfected with an NFκB-RE luciferase reporter plasmid were treated for 2 h with BRD inhibitors then stimulated with 10 ng/mL TNF-α for 4 h before luciferase activity was determined. Signal was normalized to cells treated with DMSO and TNF-α on the same plate. (d) Cytokine mRNA expression in THP-1 cells following a therapeutic treatment protocol with BRD inhibitors: 5 h 10 ng/mL TNF-α with 1 μM compounds added 1.5 h after TNF-α stimulation. mRNA expression quantified by RT-qPCR, normalized to *hprt* and then to cells treated with TNF-α and DMSO in the same experimental run. Raw *C*_p_ values shown in Figure S6. Cellular viability of (e) THP-1 and (f) MRC5 cells following three-day treatment with compounds. Viability determined using resazurin and normalized to DMSO treated cells on the same plate.

To determine if the CBP/EP300-BRD contributes to this TNF-α-induced cytokine expression, THP-1 cells were co-treated for 1 h with 10 ng/mL TNF-α and 1 μM of **2** or **5**. The commercial CBP/EP300-BRD inhibitor GNE-272 ^25^ as well as the CBP/EP300-HAT inhibitor A485 ^28^ were included for comparison. Promisingly, CBP/EP300-BRD inhibition led to a strong and highly significant reduction in the TNF-α-induced expression of *il1*β, *il*8, *mcp-1* and *tnf*-α (Figures 3b and S4). In this single-dose set-up, GNE-272 and compound **2** inhibited expression to a greater extent than the less potent but more soluble inhibitor **5**. Likewise, the HAT inhibitor A485 significantly reduced the TNF-α-induced expression of all four tested genes. Among these cytokines, only the protein levels of IL-8 could be quantified, with **2**, GNE-272 and A485 significantly reducing its TNF-α-induced secretion (Figure S5). Additionally, both CBP/EP300-BRD and HAT inhibition significantly reduced the expression of these cytokines in the absence of TNF-α (Figures 3b and S4).

It is well established that TNF-α strongly induces NFκB-regulated gene expression, as shown here using an NFκB-response element (RE) luciferase reporter assay (Figure 3c). We hypothesized that CBP/EP300-BRD inhibition may have its effect on cytokine gene expression by affecting NFκB signaling and indeed all three BRD inhibitors were able to partially block TNF-α-stimulated NFκB-mediated gene expression with their relative activities in line with their BRD binding affinities (Figure 3c). It is thus clear that the CBP/EP300-BRD plays an important role in reaching the maximal TNF-α-induced NFκB activity, but is not essential for this pathway as its inhibition did not entirely reduce the activity to the level of unstimulated cells even at saturating inhibitor concentrations.

In a clinical setting, treatment occurs following the onset of inflammation so we also determined if CBP/EP300-BRD inhibition can reduce pro-inflammatory cytokine expression following a therapeutic paradigm. Similarly to co-administration, treatment with CBP/EP300-BRD inhibitors 1.5 h after TNF-α stimulation significantly reduced the expression of *il1*β, *il*8, *mcp-1* and *tnf*-α (Figures 3d and S5).

Compound **2** was able to strongly inhibit the growth of leukemia, melanoma and breast cancer cell lines in the NCI-60 antiproliferation screen^50^ (Table S3), in agreement with the published anti-proliferative effects of CBP/EP300-BRD inhibitors in leukemia lines.^24,25,51^ It was, thus, not surprising to observe a reduction in THP-1 cell proliferation following a 3 day treatment with all of our tested compounds (Figure 3e), likely due, at least in part, to their effects on *myc* expression (Figure 2e,g). To determine if these compounds are generally cytotoxic, their anti-proliferative effects were also investigated in MRC5 cells, a lung fibroblast line derived from normal tissue.^52^ In these non-cancerous cells, the anti-proliferative effect of CBP/EP300-BRD inhibition was almost completely lost (GI_30_ > 10 μM [**2**, **5** and GNE-272]), in contrast to A485 which continued to be toxic even at lower concentrations (GI_30_ = 1.2 μM, Figure 3f). These results demonstrate that inhibiting the CBP/EP300-BRD and HAT domains strongly interferes with the initiation of the inflammatory cascade, and highlight the reduced general cytotoxicity of targeting the BRD rather than the catalytic HAT domain of CBP/EP300.

### Development of a Lymphatic Model of TNF-α-Induced Inflammation

To evaluate the anti-inflammatory effect of the BRD inhibitors **2** and **5** *in vivo*, we developed a novel murine model of TNF-α-induced inflammation in the lymphatic system by injecting 300 ng of recombinant murine TNF-α (rmTNF-α) subcutaneously (s.c.) into the mouse footpad (Figure S7a). We have previously applied a similar approach to study the induction of local inflammation mediated by IFN-β and IL-1α.^53^ We expect that after injection rmTNF-α will be transported via lymphatic drainage to the popliteal lymph node (pLN), where it will trigger an inflammatory response (Figure S7a), and indeed we observed a significant increase in the secreted levels of the inflammatory cytokines IL-1α, MCP-1, IL-6, IL-17a and TNF-α at 3 h post-administration of 300 ng of rmTNF-α (Figure S7b). Furthermore, there was a trend towards increased secretion of IL-1β but the effect after 3 h was not statistically significant (Figure S7b).

### CBP/EP300-BRD Inhibition Reduces TNF-α-Induced Inflammation

To confirm if **2** and **5** were able to inhibit the production of the previously described inflammatory cytokines, we administered 12.5% CAPTISOL solutions of CBP/EP300-BRD inhibitors at different concentrations (Table S4) by s.c. (footpad) and intraperitoneal (i.p.) injection 90 min after rmTNF-α, which was also administered s.c. in the footpad and i.p (Figure 4a). We then measured the concentration of several inflammatory cytokines in the pLN at 5 h post-rmTNF-α administration using the LEGENDplex assay (Biolegend). Following this approach, we observed a significant reduction in the concentrations of the inflammatory cytokines IL-1β, MCP-1, IL-1α and IL-6 in the groups treated with **2** or **5**, compared with the group injected with rmTNF-α and CAPTISOL (Figure 4b–e). The magnitude of the reduction was equivalent to that with GNE-272 for all cytokines except for IL-6, for which we could observe a more considerable decrease with **2** (4-fold) and **5** (7-fold) compared to GNE-272 (Figure 4e). The observed reduction in cytokines may result from the inhibition of the NFκB pathway, as per our *in vitro* studies, as the IL-1 family has been reported to be linked to this pathway^54^ and the induction of IL-6 release by TNF-α has been previously associated with the inhibitory kappa B (IκB)-NFκB, p38 mitogen-activated protein (MAP) kinase and stress-activated protein kinase (SAPK)/c-Jun N-terminal kinase (JNK)^55^ pathways. The immunomodulatory effects of CBP/EP300-BRD inhibition are highlighted here by the inhibition of MCP-1 (CCL2), a potent chemoattractant responsible for the recruitment of monocytes/macrophages to the lymphatic compartment.^56^ Moreover, these inhibitors additionally reduce the TNF-α-induced production of the pleiotropic cytokine IL-6 whose dysregulation is associated with the progression of several diseases such as diabetes, RA, and Crohn’s disease.^57^

**Figure 4:**
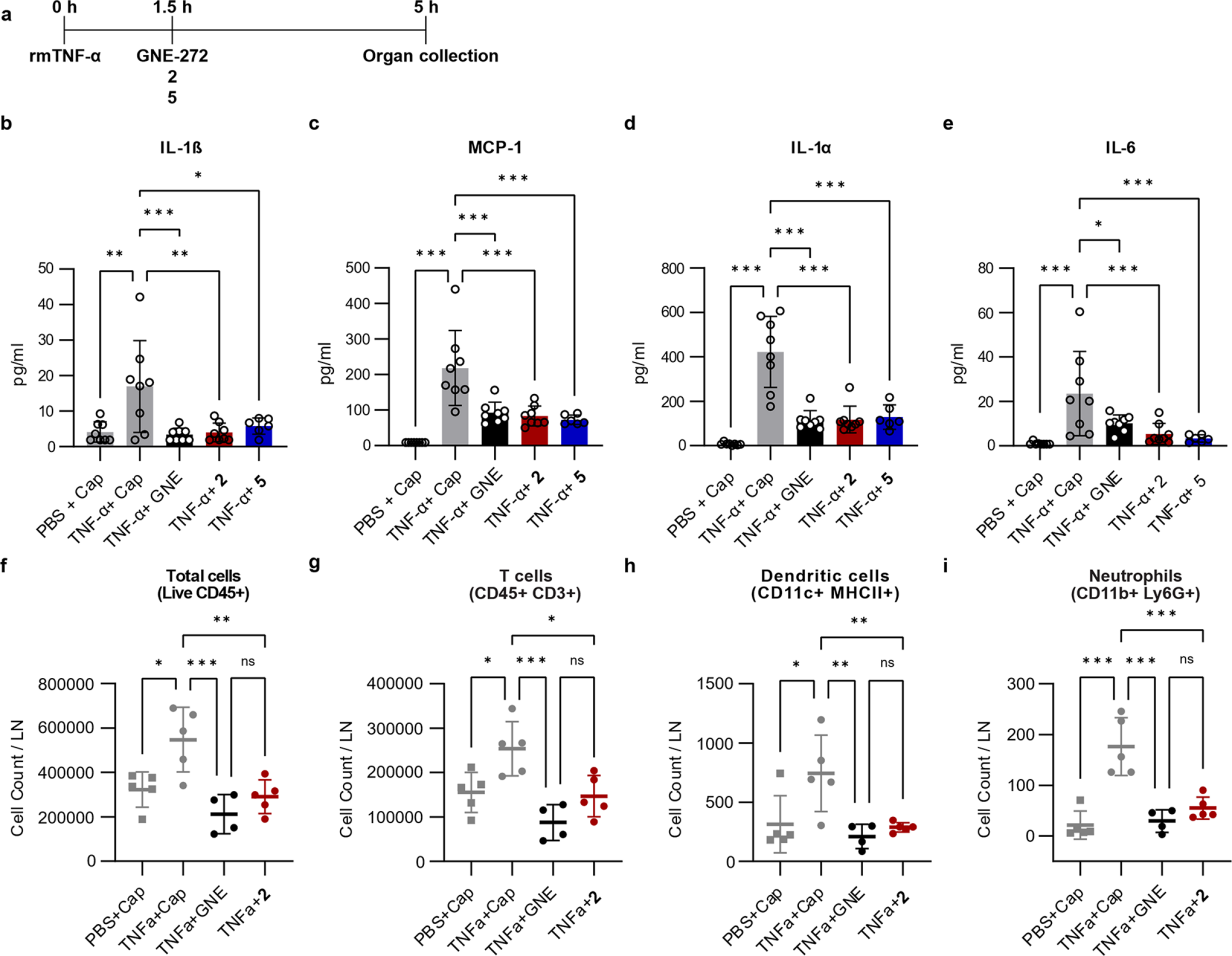
CBP/EP300-BRD inhibition reduces TNF-α-induced inflammation *in vivo*. (a) Schematic representation of the experimental set-up. Mice were injected s.c. (footpad) and i.p. with 300 ng of rmTNF-α 90 min before administering s.c. (footpad) and i.p. 10 μL each of the CAPTISOL (abbr. Cap) solutions of **2** (160 ± 1 μM), **5** (790 ± 18 μM) or GNE-272 (715 ± 12 μM) (abbr. GNE). Organs were collected for analysis 5 h post-rmTNF-α administration. Total concentration of IL-1β (b), MCP-1 (c), IL-1α (d), and IL-6 (e), and flow cytometry analysis showing the absolute counts of total lymphocytes (f), T cells (g), dendritic cells (h), and neutrophils (i) in the pLN.

To further characterize the action of the tested compounds on the immune system, we also performed flow cytometric analysis of the immune cell population in the pLN at 5 h post-injection of rmTNF-α in the presence or absence of **2** or GNE-272. Interestingly, we found that both compounds significantly inhibited the recruitment of lymphocytes, including T and B cells (Figures 4f,g and S7c, respectively), as well as dendritic cells (DC) and neutrophils (Figure 4h,i, respectively), compared to the control group. Furthermore, we could observe a significant reduction in recruitment of both CD11b- and CD11b+ DC subpopulations (Figure S7d,e). This effect could be beneficial for the treatment of autoimmune inflammatory conditions such as RA, in which neutrophil depletion has been associated with the amelioration of disease severity in an experimental arthritis mouse model.^58^

## Conclusions

While CBP/EP300 are known to play a role in NFκB activity in inflammation, the involvement of their BRD in these mechanisms is much less established and inconclusive. Here, we developed a novel, structurally distinct class of CBP/EP300-BRD binders featuring a 3-methylcinnoline as an acetyl-lysine mimicking fragment. This motif, identified *in silico* by library docking was used as the basis for a structure-based hit-to-lead optimization, culminating in highly potent, selective, and cell permeable compounds. The introduced structural novelty affords a distinct selectivity profile, with identified off-targets differing from other published inhibitors utilizing different acetyl-lysine mimics, for example GNE-272 and SGC-CBP30.^25,36^ With our cinnoline derivatives **2** and **5**, we have demonstrated both *in vitro* and *in vivo* that the CBP/EP300-BRD plays a critical role in regulating TNF-α-induced NFκB activity. Our results demonstrate that inhibition of the CBP/EP300-BRD interferes with the inflammatory pathways triggered by TNF-α, thus reducing cytokine expression, production, and the subsequent recruitment of immune cells. As similar results were obtained with GNE-272, a reported structurally distinct chemical probe, this adds confidence that the observed effects are due to on-target engagement of the CBP/EP300-BRD. This work provides tool compounds to further unravel the mechanism through which the CBP/EP300-BRD affects NFκB activity, to identify other pathways through which CBP/EP300-BRD affects inflammatory gene expression, and to determine the contribution of the CBP/EP300-BRD to TNF-α-mediated disease in a more physiological context. The absence of *in vitro* cytotoxicity of our CBP/EP300-BRD inhibitors opens a promising avenue for the treatment of acute inflammation and for clinical applications in RA or other TNF-α-mediated diseases.

## Supplementary Material



## Data Availability

The X-ray structural data of CBP-BRD complexed with ligand **1** generated in this study have been deposited in the RSCB PDB database under accession code 6SQM. This data can be obtained free of charge from RSCB Protein Data Bank via http://www.rcsb.org. The authors declare that the data supporting the findings of this study are available within the paper and its Supporting Information files. Crystallographic data, details of n numbers and statistical tests, chemical synthesis experimental procedures, compound characterization data, NMR spectra, and HPLC traces are available in the Supporting Information. Data is available from the corresponding authors upon request.

## Notes

Supplementary figures and tables, crystallographic data, materials and methods, details of n numbers and statistical tests, detailed synthetic procedures and compound characterization data, NMR spectra, and HPLC traces (PDF)
