# Supplementary material for: Selective CBP/EP300 Bromodomain Inhibitors: Novel Epigenetic Tools to Counter TNF-α-Driven Inflammation
Source: JACS Au. 2025 Jun 5;5(6):2491–9. doi: 10.1021/jacsau.5c00085 (PMC12188386; doi:10.1021/jacsau.5c00085)
Supplement: Supplementary file 1 [file au5c00085_si_001.pdf]

# Selective CBP/EP300 Bromodomain Inhibitors: Novel Epigenetic Tools to Counter TNF-α-driven Inflammation

*Katherine A. Gosselé,[1,2,‡] Irene Latino,[3,‡] Eleen Laul,[1,‡] Mariia S. Kirillova,[1] Vlad Pascanu,[1] Emanuele Carloni,[3] Rajiv K. Bedi,[2] Chiara Pizzichetti,[3] Amedeo Caflisch,[2,*] Santiago F. González,[3,*] Cristina Nevado[1,*]*

*1: Department of Chemistry, University of Zurich, Zurich, Switzerland*

*2: Department of Biochemistry, University of Zurich, Zurich, Switzerland*

*3: Faculty of Biomedical Sciences, Università della Svizzera Italiana, Institute for Research in Biomedicine, Bellinzona, Switzerland*

[‡]*These authors contributed equally*

## Supporting information

**Table of Contents**

Supplementary Figures ..... 2

Supplementary Tables ..... 5

Materials and Methods ..... 12

Details of n numbers and statistical tests ..... 20

Synthetic Experimental Procedures, Schemes and Compound Characterisation ..... 25

$^{1}$H and $^{13}$C NMR Spectra and Chiral HPLC Traces ..... 36

References ..... 54

# Supplementary Figures

a

| Target Gene Symbol | 2 %Ctrl @1 µM | Target Gene Symbol | 2 %Ctrl @1 µM |
|---|---|---|---|
| *ATAD2A* | 84 | BRPF3 | 83 |
| ATAD2B | 89 | CECR2 | 79 |
| BAZ2A | 85 | CREBBP | 0 |
| BAZ2B | 90 | EP300 | 0 |
| BRD1 | 86 | FALZ | 97 |
| BRD2(1) | 47 | GCN5L2 | 33 |
| BRD2(2) | 90 | PBRM1(2) | 89 |
| BRD3(1) | 91 | PBRM1(5) | 65 |
| BRD3(2) | 88 | PCAF | 97 |
| BRD4(1) | 68 | SMARCA2 | 49 |
| BRD4(2) | 91 | SMARCA4 | 33 |
| BRD7 | 72 | TAF1(2) | 83 |
| BRD9 | 53 | TAF1L(2) | 95 |
| BRDT(1) | 62 | TRIM24(PHD, Bromo.) | 100 |
| BRDT(2) | 69 | TRIM33(PHD, Bromo.) | 72 |
| BRPF1 | 88 | WDR9(2) | 70 |

b

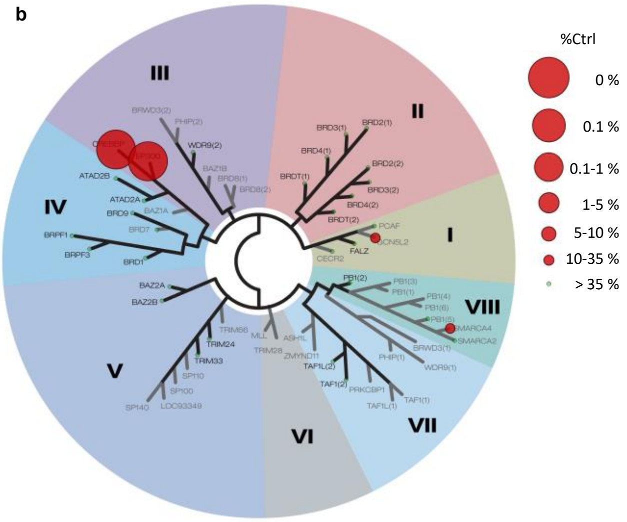


**Figure S1:** Selectivity of **2** across a panel of 32 bromodomains as determined by the bromoMAX product of the BROMOscan™ Profiling service.[1,2] a) Binding profile of 1 µM **2**; percentage of control (%Ctrl) representing the inhibitory effect of **2** in comparison to a negative control (DMSO, 100%) and a positive control compound (0%). b) TREEspot™ Interaction Map visualising the selective inhibitory effect of **2** over all the BRD families.

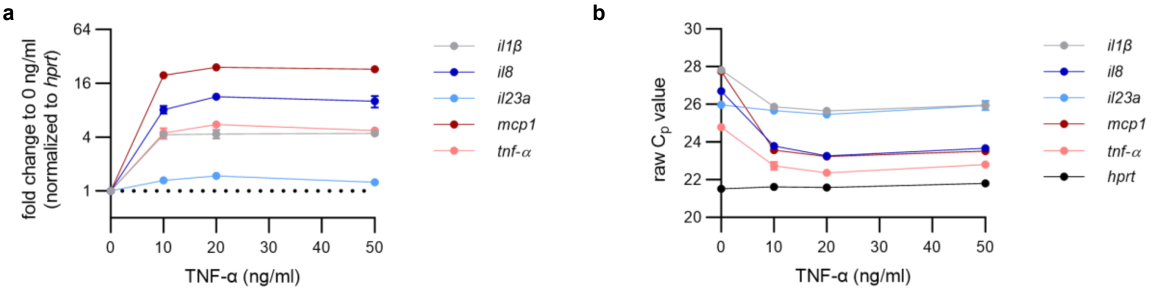


**Figure S2:** Cytokine mRNA expression in THP-1 cells upon TNF-α treatment. a) Cytokine mRNA expression in THP-1 cells, following treatment with different concentrations of TNF-α for 5h. Gene expression was determined by RT-qPCR and normalized first to *hprt* and then to untreated cells. n = 2 for 10 and 50 ng/mL, and n = 1 for 0 and 20 ng/mL TNF-α. b) Raw $C_p$ values (maximum of the second derivative) used to calculate the relative expression shown in a).

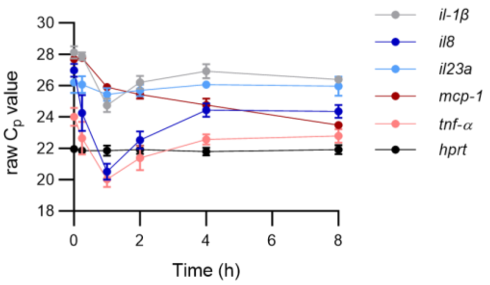


**Figure S3:** Raw $C_p$ values used for the calculation of the relative expression displayed in Figure 3a. *hprt* was used for normalization.

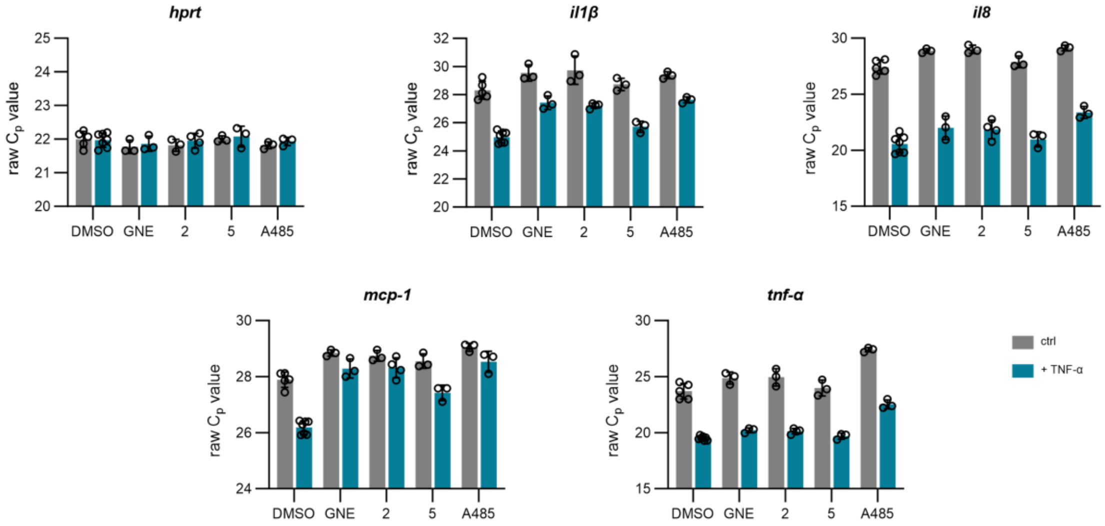


**Figure S4:** Raw $C_p$ values used for the calculation of the relative expression displayed in Figure 3b. *hprt* was used for normalization.

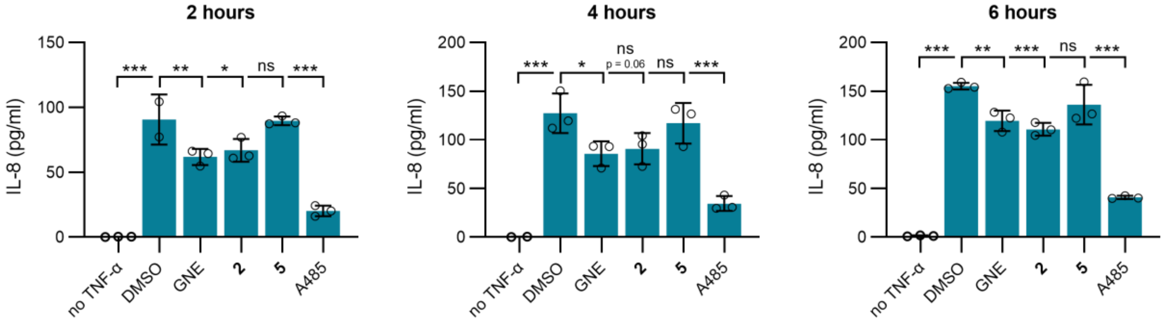


**Figure S5:** IL-8 protein secretion from THP-1 cells following co-treatment with 10 ng/mL TNF-α and 1 µM GNE-272 (abbr. GNE), **2**, **5** or A485.

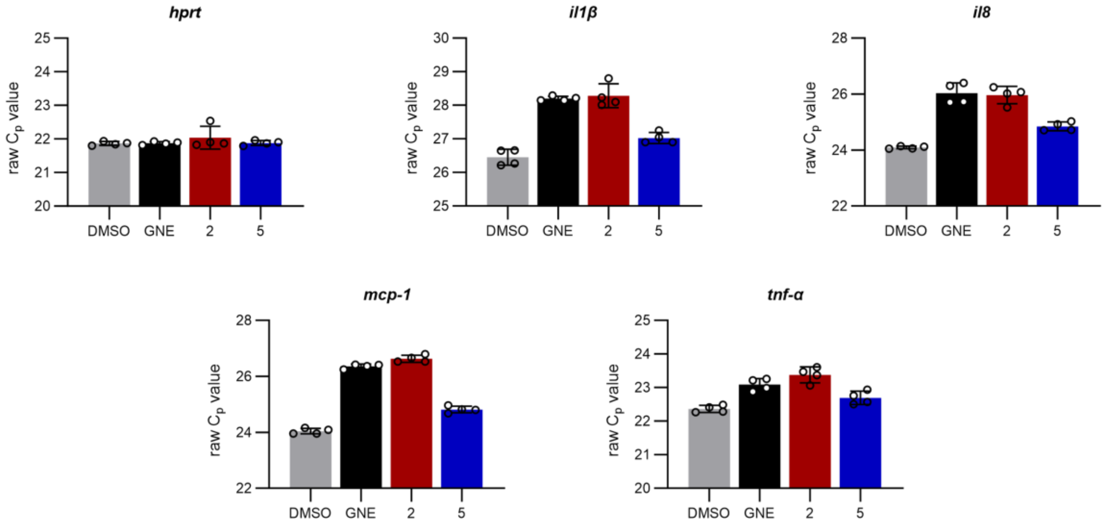


**Figure S6:** Raw $C_p$ values used for the calculation of the relative expression displayed in Figure 3d. *hprt* was used for normalization.

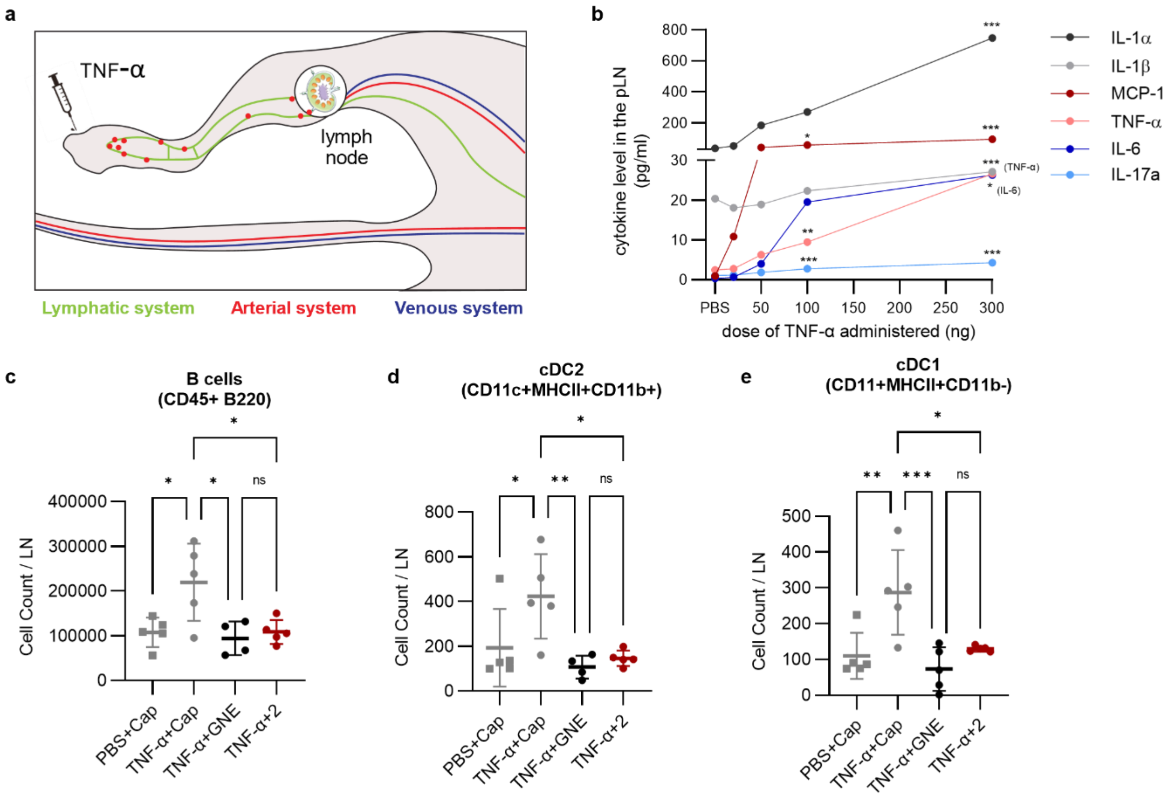


**Figure S7:** CBP/EP300-BRD inhibition reduces TNF-α-induced inflammation in a murine model. a) Murine lymphatic model of TNF-α induced inflammation: recombinant murine TNF-α (rmTNF-α) was injected subcutaneously and transported to the draining popliteal lymph node (pLN). (b) Levels of pro-inflammatory cytokines including IL-6, MCP-1, IL-1α, IL-1β, and IL-17a, induced by the administration of rmTNF at 3 h post-administration. Flow cytometry analysis showing the absolute counts of B cells (B220+) (c), CD11c+ DC (d), CD11c- DC (e), in the pLN at 5 h post-rmTNF-α administration.

## Supplementary Tables

**Table S1**: A representative selection of published CBP/EP300-BRD inhibitors in chronological order, with KAc mimicking fragments depicted in blue.

| Name and structure | Affinity to CBP-BRD (method) | Sel. over BRD4(1)-BRD | Ref. |
|---|---|---|---|
| SGC-CBP30 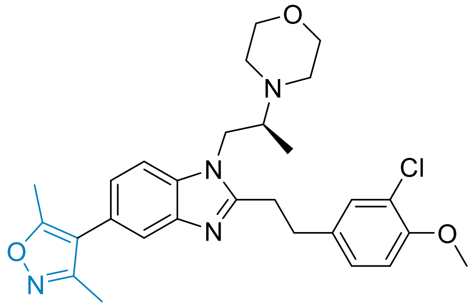 | $K_d$ = 21 nM (ITC) | 40-fold | (Hay et al., 2014)[3] |
| I-CBP112 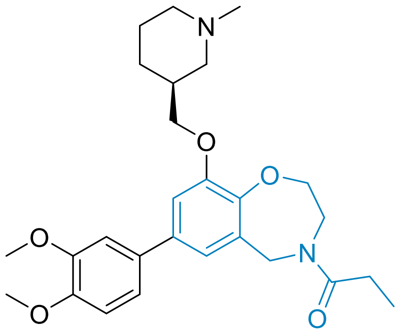 | $K_d$ = 151 nM (ITC) | 37-fold | (Picaud et al., 2015; Popp et al., 2016)[4,5] |
| CPI-637 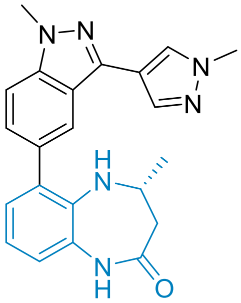 | $IC_{50}$= 30 nM (TR-FRET) | 367-fold | (Taylor et al., 2016)[6] |
| GNE-272 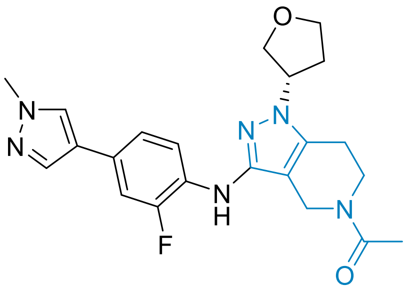 | Published $IC_{50}$= 20 nM (TR-FRET); In house $IC_{50}$= 12 nM (TR-FRET) | 650-fold | (Crawford et al., 2016)[7] |

| | | | |
|---|---|---|---|
| GNE-781 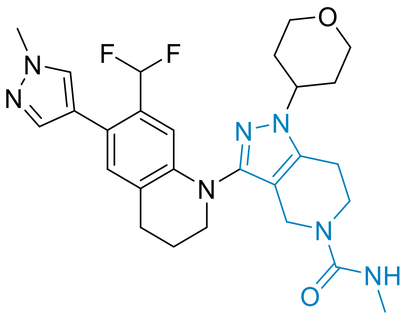 | $IC_{50}$= 0.9 nM (TR-FRET) | 5425-fold | (Romero et al., 2017)[8] |
| CCS1477 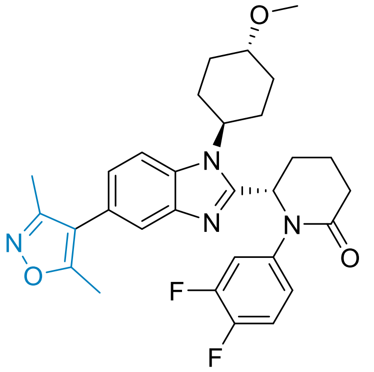 | $K_d$ = 1.7 nM (SPR) | 130-fold | Used in clinical trials. (Pegg et al., 2017; Brooks et al., 2017)[9,10] |
| 16 in Batiste et al. 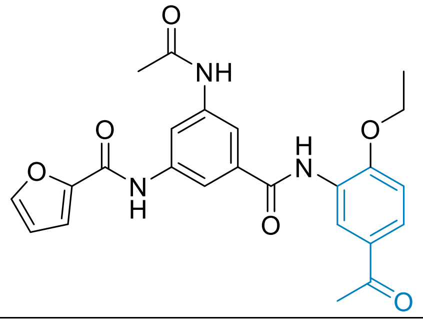 | $K_d$ = 19 nM (Alphascreen) | 10526-fold | Our previous work: (Batiste et al., 2018)[11] |
| UMB298 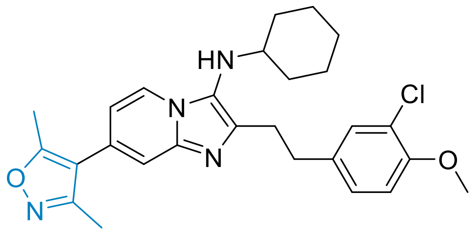 | $K_d$ = 72 nM (Alphascreen) | 72-fold | (Muthengi et al., 2021)[12] |

**Table S2:** Results for Caco-2 bidirectional permeability assay by Cyprotex Discovery (Cheshire, UK): Apprent permeability coefficients ($P_{app}$) along with mean recovery values for both apical to basolateral and basolateral to apical direction and the efflux ratio ($P_{app(B-A)}/P_{app(A-B)}$).

| Direction: A-B (apical-to-basolateral) | | | | | | | |
|---|---|---|---|---|---|---|---|
| Compound | $P_{app}$ ($10^{-6}$ $cms^{-1}$) | | Mean $P_{app}$ ($10^{-6}$ $cms^{-1}$) | SD | n | Mean % Recovery | |
| | Replicate 1 | Replicate 2 | | | | | |
| **1** | 0.0348 | 0.0401 | 0.0375* | 0.00371 | 2 | 80 | |
| **2** | 0.565 | 0.533 | 0.549 | 0.0227 | 2 | 47 | |
| **5** | 0.57 | 0.677 | 0.623 | 0.0759 | 2 | 68.2 | |
| Direction: B-A (basolateral-to-apical) | | | | | | | |
| Compound | $P_{app}$ ($10^{-6}$ $cms^{-1}$) | | Mean $P_{app}$ ($10^{-6}$ $cms^{-1}$) | SD | n | Mean % Recovery | Efflux Ratio |
| | Replicate 1 | Replicate 2 | | | | | |
| **1** | 11.6 | 11.5 | 11.5 | 0.0675 | 2 | 87.3 | 307 |
| **2** | 28.9 | 29.2 | 29 | 0.157 | 2 | 61.8 | 52.9 |
| **5** | 34.2 | 30.9 | 32.5 | 2.38 | 2 | 78 | 52.2 |

**Test compound sample concentration from the received compartment measured below the lower quantification signal – accuracy of the result likely affected.*

**Table S3:** NCI-60 Screen[13] results: Percent growth (%Growth)* of cancer cells upon treatment with 10 μM **2**.

| Panel | Cell Name | %Growth* | Panel | Cell Name | %Growth* |
|---|---|---|---|---|---|
| Leukemia | CCRF-CEM | 43 | CNS Cancer | SF-268 | 72 |
| | HL-60(TB) | 25 | | SF-295 | 42 |
| | K-562 | 29 | | SF-539 | 44 |
| | MOLT-4 | 53 | | SNB-19 | 66 |
| | RPMI-8226 | 32 | | SNB-75 | 27 |
| | SR | 19 | | U251 | 61 |
| Renal Cancer | 786-0 | 57 | Prostate Cancer | PC-3 | 54 |
| | A498 | 91 | | DU-145 | 87 |
| | ACHN | 73 | Breast Cancer | MCF7 | 22 |
| | CAKI-1 | 22 | | MDA-MB-231/ATCC | 57 |
| | RXF 393 | -5 | | HS 578T | 19 |
| | SN12C | 63 | | BT-549 | 33 |
| | TK-10 | 78 | | T-47D | 44 |
| | UO-31 | 43 | | MDA-MB-468 | -19 |
| Colon Cancer | COLO 205 | 32 | Ovarian Cancer | IGROV1 | 54 |
| | HCC-2998 | 66 | | OVCAR-3 | 23 |
| | HCT-116 | 38 | | OVCAR-4 | 63 |
| | HCT-15 | 64 | | OVCAR-5 | 89 |
| | HT29 | 21 | | OVCAR-8 | 67 |
| | KM12 | 28 | | NCI/ADR-RES | 91 |
| | SW-620 | 52 | | SK-OV-3 | 86 |
| Non-Small Cell Lung Cancer | A549/ATCC | 61 | Melanoma | LOX IMVI | 34 |
| | EKVX | 63 | | MALME-3M | 40 |
| | HOP-62 | 67 | | M14 | 25 |
| | HOP-92 | 63 | | MDA-MB-435 | -47 |
| | NCI-H226 | 49 | | SK-MEL-2 | 18 |
| | NCI-H23 | 71 | | SK-MEL-28 | 68 |
| | NCI-H322M | 86 | | SK-MEL-5 | -36 |
| | NCI-H460 | 18 | | UACC-257 | 66 |
| | NCI-H522 | 3 | | | |

** The reported %Growth is relative to no-drug control and relative to the time zero number of cells: values from 100-0 indicate growth inhibition; values <0 indicate lethality. The values are coloured by least growth inhibition (blue) to most growth inhibition/lethality (red).*

**Table S**: Absorption wavelengths (λ) used for calibration curve and determining compound concentration in the kinetic solubility assay, and the mean compound concentrations obtained in the 12.5% CAPTISOL® in water solution.

| Cmpd. | λ used for cal. curve (nm) | Conc. in 12.5% Captisol sol. (μM) |
|---|---|---|
| ***1*** | 260 | n/a |
| ***2*** | 260 | 160 ± 1* |
| ***3*** | 260 | n/a |
| ***4*** | 270 | n/a |
| ***5*** | 250 | 790 ± 18* |
| ***6*** | 260 | n/a |
| ***7*** | 270 | n/a |
| ***8*** | 290 | n/a |
| ***GNE272*** | 280 | 715 ± 12* |

**st. dev. between technical replicates (n=2)*

**Table S5:** X-ray data collection and refinement statistics for complex structures of the CBP bromodomain with **1**.

| **PDB ID:** | 6SQM |
|---|---|
| **Compound** | **1** |
| **Data Collection** | |
| Space group | C 1 2 1 |
| Cell dimension a, b, c (Å) | 126.8, 37.6, 90.46 |
| Cell dimension α, β, γ (°) | 90, 116.2, 90 |
| Resolution (Å)* | 43.2 (1.8) |
| Unique reflections* | 35264 (5556) |
| Completeness* | 98.4 (97.6) |
| Redundancy* | 3.39 (3.5) |
| $R_{merge}$* | 6.1 (61.9) |
| CC (1/2)* | 99.9 (88.6) |
| I/σI* | 16.16 (2.2) |
| **Refinement** | |
| $R_{work}/R_{free}$ | 0.2080/0.269 |
| RMSD bond (Å) | 0.007 |
| RMSD angle (°) | 0.968 |
| B-factor (Å$^2$)** | 25.55/27.76/33.05 |
| Ramanchandran Favored | 99.7 |
| Ramanchandran allowed | 0.3 |
| Ramanchandran Disallowed | 0 |

**Statistics for the highest resolution shell are shown in parentheses.*

*** P/L/W indicate protein, ligand/ion and water molecules, respectively.*

**Table S6**: Primer sequences used to determine gene expression by RT-qPCR. In all cases, *hprt* was used as the housekeeping gene for normalization.

| **Gene Name** | **Gene ID** | **Forward Primer (5'-3')** | **Reverse primer (5'-3')** |
|---|---|---|---|
| *hprt* | NM_000194.3 | CCCTGGCGTCGTGATTAGTG | TCGAGCAAGACGTTCAGTCC |
| *myc* | NM_001354870.1 | GTAGTGGAAAACCAGCC | AGAAATACGGCTGCACCGAG |
| *il1α* | NM_000575.5 | CAGTGCTGCTGAAGGAGATGC | TGGATGGGCAACTGATGTGAA |
| *il1β* | NM_000576.3 | GCCCTAAACAGATGAAGTGCTC | CCATGGCCACAACAACTGAC |
| *il6* | NM_000600.5 | TCCAATCTGGATTCAATGAGGAGA | GTCAGGGGTGGTTATTGCATC |
| *il8 (cxcl8)* | NM_000584.4 | TGCGCCAACACAGAAATTATTGTAA | CCTCTGCACCCAGTTTTCCT |
| *il23α* | NM_016584.3 | ATCCAGTGTGGAGATGGCTGT | GGATCCTTTGCAAGCAGAACTGAC |
| *mcp-1 (ccl2)* | NM_002982.4 | TCAAACTGAAGCTCGCACTCT | GGCATTGATTGCATCTGGC |
| *tnf-α* | NM_000594.4 | TGTAGCCCATGTTGTAGCAAACC | TGGTTATCTCTCAGCTCCACG |

# Materials and Methods

**Safety**
No unexpected or unusually high safety hazards were encountered.

**Chemicals**
GNE-272 and A485 were purchased from MedChemExpress (HY-100726, HY-107455), and CAPTISOL® from CyDex Pharmaceuticals. All other chemicals were synthesized and characterized in house (see section: Synthetic Experimental Procedures, Schemes and Compound Characterisation).

**Protein expression and purification**
The plasmid expressing the N-terminally hexahistidine-tagged CBP bromodomain (residues 1081-1197) of human CBP protein was a gift from Nicola Burgess-Brown (Addgene ID: 38977). Escherichia coli BL21 (DE3) cells transformed with the expression plasmids were plated out onto LB-agar plates containing 50 µg/mL kanamycin. The protein was overexpressed for 16 h at 18°C in Escherichia coli BL21 (DE3) cells upon induction with 0.2 mM IPTG. The recombinant protein was purified to homogeneity in two chromatographic steps. The cells were harvested and resuspended in lysis buffer containing 100 mM Tris-HCl at pH 8.0, 500 mM NaCl and 10 mM imidazole. The cells were lysed by sonication and the cell lysate was clarified by centrifugation at 48,000 g for 1 h and loaded onto a Ni-NTA affinity column (5 mL HisTrap FF from GE Healthcare). After extensive washing with wash buffer containing 100 mM Tris-HCl at pH 8.0, 500 mM NaCl and 50 mM imidazole, the target protein was eluted with elution buffer containing 100 mM Tris-HCl at pH 8.0, 500 mM NaCl and 250 mM imidazole. The N-terminal hexahistidine-tag was removed by cleavage with tobacco etch virus (TEV) protease at 1:50 ratio. The excess imidazole was removed by overnight dialysis and the sample was subjected to a secondary subtractive Ni-NTA affinity chromatography step to remove the protease and uncleaved protein. Finally, the protein was subjected to a gel filtration step using Superdex 75 16/60 column in a buffer containing 10 mM HEPES at pH 7.4, 150 mM NaCl and 5% glycerol. The protein was concentrated to 10 mg/mL, flash-frozen in liquid nitrogen and stored at -80 °C for future experiments.

**Protein production for TR-FRET**
GST-CBP (1081-1197) was created by cloning the CBP bromodomain into the pGEX_6P_1 vector between EcoRI and XhoI restriction sites using His-CBP plasmid as a template. This GST-CBP construct was overexpressed in Rosetta (DE3) cells upon induction with 100 µM isopropyl thio-beta-D-galactoside (IPTG) for 20 h at 18 °C. Harvested cells were resuspended in lysis buffer containing 100 mM TrisHCl, pH 8.0, 500 mM NaCl, 1 mM 1,4-dithiothreitol (DTT), 1 mM phenylmethylsulfonyl fluoride (PMSF), and 1 mM ethylenediaminetetraacetic acid (EDTA) and lysed by sonication. The lysates were centrifuged at 18 000 rpm at 4 °C for 1 h. The soluble proteins were loaded onto a column packed with glutathione sepharose 4B (GE Healthcare) and subsequently eluted with 10 mM reduced glutathione in buffer containing 20 mM Tris-HCl, pH 8.0, and 150 mM NaCl. Finally, a size exclusion step (HiLoad 16/600 Superdex 200 pg column from GE Healthcare) was used to further purify the protein in 50 mM HEPES, pH=7.5, 150 mM NaCl buffer. The purified protein was aliquoted and stored at −80 °C until use.

**Crystallization and data processing**

The purified CBP protein at 10 mg/mL was mixed with compound **1** at a 1:3 (protein:compound) molar ratio and was incubated at 4°C for 1 h. The mixture was centrifuged at 15,000 g for 10 minutes before setting up crystallization trials.

Crystals of CBP in complex with **1** were obtained by mixing 0.3 µL protein-ligand complex solution with 0.3 µL mother liquor containing 8% Ethylene glycol, 0.1M HEPES pH 7.5, 10% PEG 8000 at 4°C in a sitting drop vapor diffusion setup. The crystals obtained were harvested and frozen in liquid nitrogen with additional cryoprotectant in the mother liquor (8% Ethylene glycol, 0.1M HEPES pH 7.5, 30% PEG 8000).

Diffraction images were collected at the Swiss Light Source (Villigen, Switzerland) using the beamline X06DA (PXIII), and processed using XDS.[14] The structure was solved by molecular replacement using Phaser program from the Phenix package.[15,16] The unliganded structure of CBP (PDB ID: 5MME) was used as a search model. The model building and refinements were performed using COOT and phenix.refine.[17,18] Data collection and refinement statistics are summarized in Table S5.

**TR-FRET assay**

For HTRF measurements, all reagents were dissolved in the assay buffer (50 mM HEPES pH 7.5, 150 mM NaCl, 0.1 % BSA) to their final concentrations and compounds were serially diluted in a 1:3 series. The final content of each 50 µL reaction was as follows: 0.1% DMSO, 10 nM GST-CBP, 60 nM SK-20 (SGRGK(Ac)GGK(Ac)GLGK(Ac)GGAK(Ac)RHRK-biotin), 100 mM KF, 7.5 nM XL665-conjugated streptavidin (Cisbio, 610SAXLB) and 0.8 nM anti-GST mAb – $Eu^{3+}$-cryptate labelled antibody (Cisbio, 61GSTKLB). Assays were carried out in duplicate on a Corning 384 U Bottom White Polystyrene plate (20 µL working volume). The samples were incubated for 3 h (20°C, dark) and the signal was detected by Infinite M1000 plate reader (Tecan). After excitation at 317 nm, emission at 620 and 665 nm was recorded. The ratio of the emissions: $F = \frac{\mathrm{acceptor}_{665\ \mathrm{nm}}}{\mathrm{donor}_{620\ \mathrm{nm}}}$ was considered for further analysis.

The maximal control contained no compounds, the blank contained no CBP and no compounds – these were replaced by the appropriate buffer (with or without DMSO). The activity values of each compound were determined according to the following equation:

$$activity\ (\%) = \frac{\Delta F}{\Delta F_{DMSO}} * 100 = \frac{F - F_b}{F_t - F_b} * 100\ ,$$

where $F_t$ is the TR-FRET signal in the absence of any compound (maximal control, 100% activity), $F_b$ the TR-FRET signal in the absence of the bromodomain (blank, 0% activity) and F the TR-FRET signal in the presence of the compound. Dose-response curves were plotted in GraphPad Prism 8.4 and fitted with nonlinear regression "log(inhibitor) vs. normalized response – variable slope", from which $IC_{50}$ values were determined.

**Solubility measurement**

Starting from an initial concentration of 100, 50 or 20 µM, a 2-fold serial dilution of the compound (10 mM stock in DMSO) was prepared in a mixture of ACN/Buffer (PBS, pH = 7.4) 2:8, with 1% DMSO. 350 µL was transferred to a 96-Well Plate (Greiner, UV-star) then UV absorption (240-400 nm, 10 nm steps) was measured. The absorption was blank-corrected and plotted against concentration. The wavelength (λ) providing the best fitting straight line

was used as the calibration curve. To obtain saturated solutions of the compounds of interest, in duplicates 4.4 µL of a 10 or 5 mM stock in DMSO was added to 436 µL Buffer (PBS, pH = 7.4), to give a final concentration of 100 or 50 µM. Samples were incubated on a shaker (500 rpm) at 37°C for 90 minutes and then filtered (Millex-GV 13 mm, 0.22 µm). 304 µL of the filtrate was mixed with 76 µL of acetonitrile on a dilution plate, from which 350 µL was transferred to a UV-plate and UV-absorption was read using a SpectraMax M5 (Molecular devices). The absorbance was blank-corrected and compared to the calibration curve to obtain the concentration of dissolved compound in the saturated solution, hence the kinetic solubility. The wavelengths used for the calibration curve are available in Table S4.

**Sample preparation in CAPTISOL®**

We aimed for 1 mL of a 1 mM or 0.5 mM solution for higher solubility (**5** and GNE-272) and lower solubility (**2**) compounds respectively in a 12.5 w/v% CAPTISOL® solution in water. In a precisely tared vial, required amounts of compounds and CAPTISOL® were weighed out as solids. Based on the known density of 12.5 % CAPTISOL® solution (d = 1.05755 g/mL at 25 °C), the precise weight of the resulting solution and so the required amount of water by weight was calculated. The water was added portion wise with agitation, vortexing and sonication cycles in between additions. The saturated solutions were then filtered (Millex-GV 13 mm, 0.22 µm). In duplicates, aliquots of the filtrates were taken and diluted with blank 12.5 % CAPTISOL® water solution in either a 1:14 (higher sol. compounds) or 1:7 (lower sol. compounds) ratio, 350 µL was transferred to a 96-Well Plate (Greiner, UV-star) then UV absorption (240-400 nm, 10 nm steps) was measured using a SpectraMax M5 (Molecular devices). The absorption was blank-corrected, compared to the calibration curve obtained during the solubility measurement method above and finally corrected for the dilution ratio to obtain the compound concentration in the saturated 12.5 % CAPTISOL® solution. The calculated concentrations are available in Table S4.

**BROMOscan™ assays** (provided by Eurofins DiscoverX, US)[1,2]

T7 phage strains displaying bromodomains were grown in parallel in 24-well blocks in an E. coli host derived from the BL21 strain. E. coli were grown to log-phase and infected with T7 phage from a frozen stock (multiplicity of infection = 0.4) and incubated with shaking at 32°C until lysis (90-150 minutes). The lysates were centrifuged (5,000 x g) and filtered (0.2µm) to remove cell debris. Streptavidin-coated magnetic beads were treated with biotinylated small molecule or acetylated peptide ligands for 30 minutes at room temperature to generate affinity resins for bromodomain assays. The liganded beads were blocked with excess biotin and washed with blocking buffer (SeaBlock (Pierce), 1 % BSA, 0.05 % Tween 20, 1 mM DTT) to remove unbound ligand and to reduce non specific phage binding. Binding reactions were assembled by combining bromodomains, liganded affinity beads, and test compounds (see below) in 1x binding buffer (17% SeaBlock, 0.33x PBS, 0.04% Tween 20, 0.02% BSA, 0.004% Sodium azide, 7.4 mM DTT). All reactions were performed in polypropylene 384-well plates in a final volume of 0.02 mL. The assay plates were incubated at room temperature with shaking for 1 h and the affinity beads were washed with wash buffer (1x PBS, 0.05% Tween 20). The beads were then resuspended in elution buffer (1x PBS, 0.05% Tween 20, 2 µM non-

biotinylated affinity ligand) and incubated at room temperature with shaking for 30 minutes. The bromodomain concentration in the eluates was measured by qPCR.

***bromoK$_D$ELECT for BRD4(1)***

Test compounds were prepared as 1000X stocks in DMSO and distributed by acoustic transfer (non-contact dispensing). The compounds were then diluted directly into the assays such that the final concentration of DMSO was 0.09%. $K_D$s were determined using an 11-point 3-fold compound dilution series with one DMSO control point, and were calculated with a standard dose-response curve using the Hill equation:

$$Response = Background + \frac{Signal - Background}{1 + ({K_D}^{Hill\ Slope}/Dose^{Hill\ Slope})}$$

The Hill Slope was set to -1. Curves were fitted using a non-linear least square fit with the Levenberg-Marquardt algorithm.

***bromoMAX (32 targets)***

Test compounds were prepared as 1000X stocks in DMSO and subsequently diluted 1:25 in monoethylene glycol (MEG). The compounds were then diluted directly into the assays such that the final concentrations of DMSO and MEG were 0.1% and 2.4%, respectively. **2** was screened at the concentration of 1000 nM, and results for primary screen binding interactions are reported as '% Ctrl', calculated using the following equation, where lower numbers indicate stronger hits in the matrix (Figure S1):

$$\%Ctrl = \left(\frac{test\ compound\ signal - positive\ control\ signal}{negative\ control\ signal - positive\ control\ signal}\right) \times 100$$

test compound = **2**
negative control = DMSO (100%Ctrl)
positive control = control compound (0%Ctrl)

**Bidirectional Caco-2 Permeability Assay** (provided by Cyprotex Discovery Ltd; Cheshire, UK)[19]
Following a standard Cyprotex procedure, Caco-2 cells (ATCC), used at passages 40–60, were seeded onto Transwell inserts at a density of 1 × $10^5$ cells/cm² and maintained in DMEM, with media changes carried out every 2–3 days. Permeability assays were performed between days 18–22 post-seeding. All procedures were conducted at 37 °C in a humidified incubator with 5% $CO_2$. On the day of the assay, monolayers were prepared by rinsing both the apical and basolateral compartments with pre-warmed Hanks' Balanced Salt Solution (HBSS) at the desired pH and incubated with HBSS on both sides for 30 minutes to equilibrate the compartments. The dosing solutions were obtained by diluting the test compounds in assay buffer to a concentration of 10 µM with a final DMSO concentration of ≤1% (v/v) and Lucifer yellow was included as a fluorescent marker to assess monolayer integrity. Analytical standards were prepared similarly, omitting the Lucifer yellow. For apical-to-basolateral (A–B) measurements, the dosing solution was applied to the apical chamber and buffer to the basolateral side, while the configuration was reversed for basolateral-to-apical (B–A) measurements. After a 120 min incubation, samples from both compartments were collected and diluted for quantification. Permeability was assessed in duplicates, and reference

compounds with known characteristics were included as controls. Quantification of test and control compounds was performed by LC-MS/MS using an 8-point calibration curve under Cyprotex standard analytical conditions. The initial concentration ($C_0$) at time zero was determined from the dosing solution, and recovery was calculated based on concentrations in both compartments.

The apparent permeability coefficient ($P_{app}$) is calculated from the equation (where dQ/dt is the drug permeation rate across the cells, $C_0$ is the concentration in the donor compartment at time zero, and A is the area of the cell monolayer):

$$P_{app} = \left(\frac{dQ/dt}{C_0 \times A}\right)$$

Efflux ratio (ER) is calculated from mean A-B and B-A data:

$$ER = \frac{P_{app(B-A)}}{P_{app(A-B)}}$$

**NCI-60 Screen**[13]

Adapted from NCI-60 Screening Methodology, one-dose screen:[20] Briefly, human tumor cell lines, grown in RPMI 1640 containing 5% FBS and 2 mM L-glutamine, were plated in 96 well plates at densities ranging from 5,000 to 40,000 cells/well 24 h prior to addition of test compounds (here 10 μM **2**). 400x solutions of test compounds were prepared in DMSO, then diluted to 2x in medium supplemented with 50 μg/mL gentamicin. 100 μl of this 2x stock was added to cells previously plated in 100 μl medium, resulting in the desired final compound concentration. Cells were fixed *in situ* with cold TCA: untreated cells at the time of compound addition (Tz) and treated cells 48 h after addition of compound (Ti). Cells were washed, then sulforhodamine B (SRB) solution (100 μl) at 0.4 % (w/v) in 1 % acetic acid was added to each well and incubated for 10 minutes at room temperature. After washing again, the plates were air dried and bound stain solubilized with 10 mM Trizma base before absorbance was measured at 515 nm.

Percentage growth (%Growth) was calculated as follows:

$$when\ Ti \geq Tz, \%Growth = \frac{Ti - Tz}{C - Tz}\ x\ 100$$

$$when\ Ti \geq Tz, \%Growth = \frac{Ti - Tz}{Tz}\ x\ 100$$

where Ti = absorbance from compound treated cells, Tz = absorbance from untreated cells at time zero, and C = absorbance from control (DMSO) treated cells.

**Cell culture**

HEK293T, MRC5 and LP1 cells were cultured in DMEM (Gibco 41966-029) containing 10 % or 15 % (LP1) FBS (Gibco 10270106 or Sigma F9665), 1 % non-essential amino acids (MRC5 only, Gibco 11140035) and 1 % penicillin-streptomycin (P/S, Gibco 15070-063). THP-1 cells were cultured in RPMI 1640 (Gibco 21875-034) containing 10 % FBS and 1 % P/S. All cells were grown in a humidified incubator with 5 % $CO_2$ at 37 °C.

**CBP-BRD InCELL Pulse™**

The CBP-BRD (residues 1083-1197) was cloned into the pICP-ePL-C vector (DiscoverX, 94-4007S) between the EcoRI and XhoI restriction sites using the hexahistidine-CREBBP plasmid as a template. $5x10^5$ HEK293T cells were plated on a 6 well plate the day prior to transfection with 1.5 µg ePL-CBP-BRD plasmid using 5 µl FugeneHD (Promega E2311) in 500 µl optiMEM® (Gibco, 31985062). Proteins were allowed to express for 20 h before cells were trypsinized, washed and resuspended in low-serum medium (DMEM with 5 % FBS). Compounds were serially diluted in DMSO at 100x, pre-diluted to 5x in complete medium (DMEM with 10 % FBS and 1 % P/S), then added to a suspension of $5x10^3$ cells, giving a final DMSO concentration of 1 %. Treated cells were incubated for 1 h at 37 °C in an incubator, followed by a heat shock for 3 min at 45 °C and recovery for 3 min at 22 °C using a thermocycler with a ramp rate of 1 °C/s (SimpliAmp, Thermo Fisher). Stabilized ePL tag was quantified using the InCELL detection Kit (DiscoverX) as per the manufacturer's protocol, using the EA-10 working detection solution and a 2 h incubation before reading luminescence with a SpectraMax M5 plate reader (Molecular devices).

**Quantitative RT-PCR**

For *myc* expression, $1x10^6$ LP1 cells were plated the day prior to a 4 h treatment with compounds at a final DMSO concentration of 0.1 %. For cytokine expression determination, $5x10^5$ THP-1 cells were plated on 12 well plates the day prior to treatment with 10 ng/mL recombinant human TNF-α (aa 77-233) protein (R&D systems, 10291-TA) and compounds at a final concentration of 0.1 % DMSO. Timings are given in the figure legends. In all cases, total RNA was extracted using the NucleoSpin® RNA kit (Machery-Nagel) and used for cDNA synthesis with the High-Capacity cDNA Reverse Transcription kit (Applied Biosystems). RT-qPCR reactions were performed using *Power*SYBR™ green PCR Master Mix (Applied Biosystems) and a LightCycler® 480 (Roche). cDNA was denatured for 10 min at 95 °C followed by 40 cycles of 15 s at 95 °C and 1 min at 60 °C. $C_p$ values were determined as the maximum of the second derivative and target gene expression was compared to that of *hprt* from the same sample using the $2^{-\Delta Cp}$ method. Gene expression was then normalized to the average of the control samples from the same day, or all biological replicates. The primer sequences used are shown in Table S6.

**Cytokine protein measurements**

THP-1 cells were plated and treated as for the quantitative RT-PCR, then the cell culture medium was harvested by pelleting the cells (300g, 3min) and taking the supernatant. Cytokine protein concentrations were determined using the LEGENDPlex™ Human Inflammation Panel 1 (BioLegend) assay with 25 µL of culture medium following the manufacturer's instructions. Samples were analyzed by flow cytometry on an LSRFortessa (BD Biosciences), and data was analyzed using LEGENDPlex software (BioLegend).

**Viability assays**

MRC5 ($2x10^3$ cells/well) or THP-1 ($8x10^3$ cells/well) cells were plated onto 96 well plates and treated with compounds at a final DMSO concentration of 0.5 % (MRC5) or 0.1 % (THP-1). After 3 days of treatment, resazurin (Acros Organics) was added at a final concentration of 86 µM, and incubated for 2h15 (MRC5) or 3 h (THP-1). Fluorescence (ex. 560 nm, em. 590 nm) was read using a SpectraMax M5 (Molecular devices). $GI_{30}$ values were calculated as the compound concentration resulting in 70 % viability compared to DMSO treated controls using a nonlinear regression fit using GraphPad Prism with the following equation: (Y=Bottom + (100-Bottom)/(1+(IC50/X)^HillSlope)).

**NFκB-luciferase assay**

The NFκB-RE luciferase plasmid, pNL3.2.NF-kB-RE [NlucP/NF-kB-RE/Hygro] vector, was purchased from Promega (N1111), amplified in *E. coli*, and purified using the NucleoSpin Xtra Midi Plus kit (Macherey-Nagel). HEK293T cells were plated on 12 well plates the day prior to transfection with 1.5 µg NFκB-RE plasmid using 4.5 µl Lipofectamine™ 2000 (Thermo Fischer) per well. The next day, transfected cells were replated onto white 96 well plates at a density of $1x10^4$ cells/well. The following day, cells were treated with test compounds for 2 h with a final DMSO concentration of 0.1 % and subsequently stimulated with 10 ng/mL TNF-α for 4 h without removal of compounds. Luciferase activity was measured after diluting cell medium 1:1 with Nano-Glo® luciferase assay reagent (Promega, N1110) using a SpectraMax M5 plate reader (Molecular devices).

**Mice**

C57BL/6 mice were bred in-house or acquired from Charles River Laboratories. Mice were maintained in specific pathogen-free facilities at the Institute for Research in Biomedicine, Bellinzona. Experiments were performed following the Swiss Federal Veterinary Office guidelines and authorized by the relevant institutional committee of the Cantonal Veterinary (Commissione cantonale per gli esperimenti sugli animali, Ticino).

**TNF-α induced inflammation model**

Recombinant Mouse TNF-α (rmTNF-α) (aa 80-235) Protein (Cat# 410-MT-050/CF) was purchased from RnDsystems® and resuspended following instruction into sterile PBS. Mice were anesthetized with isoflurane and 300 ng of rmTNF-α was administered subcutaneously in the footpad (10 µL injection volume), and for the experiments with the CBP/EP300 inhibitors an additional 300 ng of rmTNF-α was also administered intraperitoneally (100 µL injection volume). After 1.5 h, mice were injected with 10 µL of the prepared CAPTISOL solutions of the CBP/EP300-BRD inhibitors (i.e. GNE-272, **2** and **5**) both subcutaneously in the foodpad and also intraperitoneally (for i.p. injection, the 10 µL of CAPTISOL solution was diluted to a final volume of 100 µL using PBS). Organs were collected 5 h after TNF-α induction.

**Cytoplex assay**

LEGENDPlexTM assays (Mouse Pro-inflammatory Chemokine Panel and Mouse Inflammation Panel; Biolegend) were performed to monitor cytokine/chemokine expression. Briefly, popliteal LNs were collected and carefully disrupted in 100 mL ice-cold phosphate buffer, minimizing cell rupture. The suspension was centrifuged at 1,500 rpm for 5 min, and the

supernatant was collected. 25 µL supernatant was used for the protocol following the manufacturer's instructions. Samples were analyzed by flow cytometry on an LSRFortessa (BD Biosciences), and data was analyzed using LEGENDPlex software (BioLegend).

**Flow cytometry**

Popliteal LNs were collected, disrupted with tweezers, and digested for 10 min at 37°C in an enzyme mix composed of DNase I (0.28 mg/mL, Amresco), dispase (1 U/mL, Corning), and collagenase P (0.5 mg/mL, Roche) in calcium- and magnesium-free PBS (PBS-, Sigma-Aldrich) followed by a stop solution composed of 2 mM EDTA (Sigma-Aldrich) and 2% heat-inactivated filter-sterilized fetal calf serum (Thermo Fisher Scientific) in PBS-. Fc receptors were blocked (aCD16/32, Biolegend) followed by surface staining and analysis by flow cytometry on an LSRFortessaTM (BD Biosciences). Where indicated, intracellular staining was performed using Intracellular Fixation and Permeabilization Buffer Set (eBioscience), following the manufacturer's instructions. Dead cells were excluded using Zombie fixable viability dye (Biolegend), and data were analyzed using FlowJo software (TriStar Inc).

**Antibodies**

In this study, various combinations of the following fluorescence-conjugated antibodies have been used for cell-surface phenotypic staining: αB220 (RA3-6B2), αCD3 (17A2), αCD11b (M1/70), αI-A/I- E (M5/114.15.2), αLy-6G (1A8), αF4/80 (BM8), αCD169 (3D6.112), αCD11c (N418), αGR-1 (RB6-8C5), αNK1.1 (PK136) (all from Biolegend).

**Data analysis and statistics**

Data was analysed and graphs prepared using GraphPad Prism. Error bars show mean ± SD. Significance is illustrated as follows: ns (or unlabelled data point) = $p > 0.05$; * = $p < 0.05$; ** = $p < 0.01$; *** = $p < 0.001$. The details of n numbers, statistical tests, and exact p-values are given in the following section.

## Details of n numbers and statistical tests

***Figure 2b, d, f:*** *Cellular target engagement by InCELL pulse.* Statistical tests not performed. n = 4 for **8** / **7**, n = 5 for **6**, n = 6 for **1** / **4** / **5** / **3**, n = 8 for **2**. Data was collected from at least two biologically independent experiments and the signal normalized to untreated cells from the same day.

***Figure 2c, e, g:*** *myc mRNA expression in LP1 cells.* Ordinary one-way ANOVA, expression from cells treated with all compounds from c, e and g compared to DMSO treated cells, giving eight comparisons in a single family (F = 39.81). Showing p-values adjusted using Dunnett's multiple comparisons test. Data was collected from three biologically independent experiments performed on different days, and expression normalized to DMSO treated cells from the same day.

| **Cpd** | **DMSO** | **1** | **GNE** | **2** | **3** | **4** |
|---|---|---|---|---|---|---|
| **n** | 14 | 6 | 7 | 6 | 6 | 6 |
| **p** | N/A | 0.9974 | <0.0001 | <0.0001 | <0.0001 | 0.9972 |

| **Cpd** | **DMSO** | **5** | **6** | **7** | **8** |
|---|---|---|---|---|---|
| **n** | 14 | 6 | 6 | 6 | 6 |
| **p** | N/A | <0.0001 | <0.0001 | 0.9368 | 0.2496 |

***Figure 3a****: Cytokine timecourse in THP-1 cells.* Ordinary one-way ANOVA performed per cytokine, each timepoint compared to time 0 h, five comparisons per family, showing adjusted p-values (Dunnett's multiple comparison test). Data was collected from three biologically independent experiments on different days, and expression normalized to untreated cells from the same day.

| **Cytokine** | **0 h** | | **0.25 h** | | **1 h** | | **2 h** | | **4 h** | | **8 h** | |
|---|---|---|---|---|---|---|---|---|---|---|---|---|
| | **n** | **p** | **n** | **p** | **n** | **p** | **n** | **p** | **n** | **p** | **n** | **p** |
| *IL1β* | 5 | N/A | 3 | 0.9997 | 3 | <0.0001 | 3 | 0.0210 | 3 | 0.4387 | 3 | 0.0602 |
| *IL8* | 5 | N/A | 3 | 0.8248 | 3 | <0.0001 | 3 | 0.0379 | 3 | 0.9564 | 3 | 0.9308 |
| *IL23a* | 5 | N/A | 3 | 0.9454 | 3 | 0.0086 | 3 | 0.0605 | 3 | 0.8692 | 3 | 0.6079 |
| *MCP1* | 5 | N/A | 3 | 0.9999 | 3 | 0.3745 | 3 | 0.0569 | 3 | 0.0022 | 3 | <0.0001 |
| *TNF* | 5 | N/A | 3 | 0.0378 | 3 | <0.0001 | 3 | <0.0001 | 3 | 0.0865 | 3 | 0.1239 |

***Figure 3b:*** *Cytokine expression following 1 h co-treatment of THP-1 cells with compounds and TNF-α.* Ordinary one-way ANOVA performed per cytokine and separately for no TNF-α (ctrl) or with TNF-α. Each compound treatment was compared to DMSO treated cells giving four comparisons per family (F = 4.619 for ctrl *il1β*, 26.32 for TNF-α *il1β*, 7.156 for ctrl *il8*, 4.812 for TNF-α *il8*, 16.86 for ctrl *mcp-1*, 82.93 for TNF-α *mcp-1*, 4.548 for ctrl *tnf-α*, 17.38 for TNF-α *tnf-α*). Showing adjusted p-values (Dunnett's multiple comparison test). Replicates of compound treatments were collected from three biologically independent experiments performed on different days. Cytokine expression was normalized to *hrpt* from the same sample, then to all the ctrl+TNF-α treated cells from all experiments.

| **TNF-α treatment** | **DMSO** | | **GNE272** | | **2** | | **5** | | **A485** | |
|---|---|---|---|---|---|---|---|---|---|---|
| | **n** | **p** | **n** | **p** | **n** | **p** | **n** | **p** | **n** | **p** |

| and cytokine | | | | | | | | | | |
|---|---|---|---|---|---|---|---|---|---|---|
| ctrl *il1β* | 5 | N/A | 3 | 0.0222 | 3 | 0.0222 | 3 | 0.4257 | 3 | 0.0301 |
| TNF-α *il1β* | 7 | N/A | 3 | <0.0001 | 4 | <0.0001 | 3 | 0.0168 | 3 | <0.0001 |
| ctrl *il8* | 5 | N/A | 3 | 0.0074 | 3 | 0.0067 | 3 | 0.3578 | 3 | 0.0051 |
| TNF-α *il8* | 7 | N/A | 3 | 0.0490 | 4 | 0.0489 | 3 | 0.6663 | 3 | 0.0068 |
| ctrl *mcp-1* | 5 | N/A | 3 | 0.0002 | 3 | 0.0004 | 3 | 0.0049 | 3 | <0.0001 |
| TNF-α *mcp-1* | 7 | N/A | 3 | <0.0001 | 4 | <0.0001 | 3 | <0.0001 | 3 | <0.0001 |
| ctrl *tnf-α* | 5 | N/A | 3 | 0.0829 | 3 | 0.0860 | 3 | 0.9007 | 3 | 0.0101 |
| TNF-α *tnf-α* | 7 | N/A | 3 | 0.0058 | 4 | 0.0106 | 3 | 0.9953 | 3 | <0.0001 |

***Figure 3c:*** *NFκB-RE luciferase reporter assay.* One-way ANOVA comparing treatment with different concentrations of inhibitors to DMSO, individually for each compound. 7 comparisons per family (F = 243.3 for GNE272, 125.7 for **2**, 38.86 for **5**). P-values adjusted using Dunnett's multiple comparisons test. Data from at least two biologically independent experiments on different days, data normalized to DMSO treated cells on the same plate.

| **Conc. (µM)** | **DMSO** | | **GNE272** | | **2** | | **5** | |
|---|---|---|---|---|---|---|---|---|
| | **n** | **p** | **n** | **p** | **n** | **p** | **n** | **p** |
| 10 | 8 (vs. GNE and **2**) / 12 (vs. **5**) | N/A | 4 | <0.0001 | 4 | <0.0001 | 6 | <0.0001 |
| 5.0 | 8 (vs. GNE and **2**) / 12 (vs. **5**) | N/A | 4 | <0.0001 | 4 | <0.0001 | 6 | <0.0001 |
| 2.5 | 8 (vs. GNE and **2**) / 12 (vs. **5**) | N/A | 4 | <0.0001 | 4 | <0.0001 | 6 | <0.0001 |
| 1.3 | 8 (vs. GNE and **2**) / 12 (vs. **5**) | N/A | 4 | <0.0001 | 4 | <0.0001 | 5 | <0.0001 |
| 0.63 | 8 (vs. GNE and **2**) / 12 (vs. **5**) | N/A | 4 | <0.0001 | 4 | <0.0001 | 6 | 0.0010 |
| 0.31 | 8 (vs. GNE and **2**) / 12 (vs. **5**) | N/A | 4 | <0.0001 | 4 | <0.0001 | 6 | 0.0160 |
| 0.16 | 8 (vs. GNE and **2**) / 12 (vs. **5**) | N/A | 4 | <0.0001 | 4 | <0.0001 | 6 | 0.7743 |

***Figure 3d***: *Cytokine mRNA expression following a therapeutic treatment protocol with BRD inhibitors.* Two-way ANOVA comparing compound treatments to DMSO for each cytokine (4 families, 3 comparisons per family). P-values adjusted using Dunnett's multiple comparisons test. Data from two biologically independent experiments on different days.

| **Cytokine** | **DMSO** | | **GNE272** | | **2** | | **5** | |
|---|---|---|---|---|---|---|---|---|
| | **n** | **p** | **n** | **p** | **n** | **p** | **n** | **p** |
| *il1β* | 4 | N/A | 4 | <0.0001 | 4 | <0.0001 | 4 | <0.0001 |
| *il8* | 4 | N/A | 4 | <0.0001 | 4 | <0.0001 | 4 | <0.0001 |
| *mcp-1* | 4 | N/A | 4 | <0.0001 | 4 | <0.0001 | 4 | <0.0001 |
| *tnf-α* | 4 | N/A | 4 | <0.0001 | 4 | <0.0001 | 4 | <0.0001 |

***Figure 3e:*** *Anti-proliferation in THP-1 cells.* One-way ANOVA comparing treatment with different concentrations of inhibitors to DMSO, individually for each compound. 8 comparisons per family (F = 195.9 for GNE272, 343.2 for **2**, 134.8 for **5**, 594.5 for A485). P-values adjusted using Dunnett's multiple comparisons test. Data from three biologically independent experiments on different days, data normalized to DMSO treated cells on the same plate.

| **Conc. (µM)** | **DMSO** | | **GNE272** | | **2** | | **5** | | **A485** | |
|---|---|---|---|---|---|---|---|---|---|---|
| | **n** | **p** | **n** | **p** | **n** | **p** | **n** | **p** | **n** | **p** |
| 10 | 96 | N/A | 6 | <0.0001 | 6 | <0.0001 | 6 | <0.0001 | 6 | <0.0001 |
| 5.0 | 96 | N/A | 6 | <0.0001 | 6 | <0.0001 | 6 | <0.0001 | 6 | <0.0001 |
| 2.5 | 96 | N/A | 6 | <0.0001 | 6 | <0.0001 | 6 | <0.0001 | 6 | <0.0001 |
| 1.3 | 96 | N/A | 6 | <0.0001 | 6 | <0.0001 | 6 | <0.0001 | 6 | <0.0001 |
| 0.63 | 96 | N/A | 6 | <0.0001 | 6 | <0.0001 | 6 | 0.1475 | 6 | <0.0001 |
| 0.31 | 96 | N/A | 6 | <0.0001 | 6 | 0.0033 | 6 | 0.5067 | 6 | <0.0001 |
| 0.16 | 96 | N/A | 6 | 0.0173 | 6 | 0.0865 | 6 | 0.9248 | 6 | <0.0001 |
| 0.078 | 96 | N/A | 6 | 0.2267 | 6 | 0.9068 | 6 | 0.9898 | 6 | <0.0001 |

***Figure 3f:*** *Anti-proliferation in MRC5 cells.* One-way ANOVA comparing treatment with different concentrations of inhibitors to DMSO, individually for each compound. 8 comparisons per family (F = 4.805 for GNE272, 15.96 for 2, 7.527 for **5**, 273.6 for A485). P-values adjusted using Dunnett's multiple comparisons test. Data from three biologically independent experiments on different days, data normalized to DMSO treated cells on the same plate.

| **Conc. (µM)** | **DMSO** | | **GNE272** | | **2** | | **5** | | **A485** | |
|---|---|---|---|---|---|---|---|---|---|---|
| | **n** | **p** | **n** | **p** | **n** | **p** | **n** | **p** | **n** | **p** |
| 10 | 122 | N/A | 8 | 0.0003 | 8 | <0.0001 | 8 | <0.0001 | 9 | <0.0001 |
| 5.0 | 122 | N/A | 8 | 0.1121 | 8 | <0.0001 | 8 | 0.2727 | 9 | <0.0001 |
| 2.5 | 122 | N/A | 8 | 0.9815 | 8 | 0.0007 | 8 | 0.9993 | 9 | <0.0001 |
| 1.3 | 122 | N/A | 8 | 0.9985 | 8 | 0.9810 | 8 | 0.9856 | 9 | <0.0001 |
| 0.63 | 122 | N/A | 8 | 0.5156 | 8 | 0.9986 | 8 | 0.0434 | 9 | <0.0001 |
| 0.31 | 122 | N/A | 8 | 0.3742 | 8 | 0.8147 | 8 | 0.7536 | 9 | <0.0001 |
| 0.16 | 122 | N/A | 6 | 0.8915 | 6 | 0.9591 | 6 | 0.7334 | 9 | <0.0001 |
| 0.078 | 122 | N/A | 6 | 0.4341 | 6 | 0.9257 | 6 | 0.0286 | 9 | 0.0548 |

***Figure 4b-e****: Cytokine levels in the pLN at 5h post-rmTNF-α administration.* Ordinary one-way ANOVA comparing compound treatments (with PBS or TNF-α) against TNF-α + captisol treatment, performed individually per cytokine, 7 comparisons per family (F = 6.188 for IL-6, 15.50 for MCP-1, 23.58 for IL1α and 4.435 for IL1β). Showing p-values adjusted using Šídák's multiple comparisons test. Data from two biologically independent experiments on different days.

| **Cytokine** | **TNF-α + Cap** | | **PBS + Cap** | | **TNF-α + GNE272** | | **TNF-α + 2** | | **TNF-α + 5** | |
|---|---|---|---|---|---|---|---|---|---|---|
| | **n** | **p** | **n** | **p** | **n** | **p** | **n** | **p** | **n** | **p** |
| IL1β | 8 | N/A | 8 | 0.0011 | 8 | 0.0005 | 8 | 0.0010 | 6 | 0.0122 |
| MCP-1 | 8 | N/A | 8 | <0.0001 | 8 | <0.0001 | 8 | <0.0001 | 6 | <0.0001 |
| IL-1 α | 8 | N/A | 8 | <0.0001 | 8 | <0.0001 | 8 | <0.0001 | 6 | <0.0001 |

| | | | | | | | | | | |
|---|---|---|---|---|---|---|---|---|---|---|
| IL-6 | 8 | N/A | 8 | <0.0001 | 8 | 0.0183 | 8 | 0.0006 | 6 | 0.0004 |

***Figure 4f-i***: *Absolute counts of total lymphocytes (CD45+), T cells (CD3+), dendritic cells (DC), Neutrophils in the pLN at 5h post-rmTNF-α administration.* Ordinary one-way ANOVA performed individually per cell type, 6 comparisons per family [TNF-α + Cap vs PBS + Cap / GNE272 / **2**, TNF-α + Cap vs. TNF-α + GNE272 / **2** and TNF-α + GNE272 vs. TNF-α + **2**]. ($F = 6.236$ for total lymphocytes, 5.279 for CD3+, 5.598 for DC, 18.71 for neutrophils). Showing p-values adjusted using Šídák's multiple comparisons test. Data from two biologically independent experiments on different days.

| **Cells** | **TNF-α + Cap vs.** | | | | | | | | | **TNF-α + GNE272 vs. TNF-α + 2** | | |
|---|---|---|---|---|---|---|---|---|---|---|---|---|
| | **PBS + Cap** | | | **TNF-α + GNE272** | | | **TNF-α + 2** | | | | | |
| | **n 1** | **n 2** | **p** | **n 1** | **n 2** | **p** | **n 1** | **n 2** | **p** | **n 1** | **n 2** | **p** |
| Total lymphocytes | 5 | 5 | 0.0192 | 5 | 4 | 0.0009 | 5 | 5 | 0.0065 | 4 | 5 | 0.8629 |
| CD3+ | 5 | 5 | 0.0399 | 5 | 4 | 0.0007 | 5 | 5 | 0.0226 | 4 | 5 | 0.4724 |
| DC | 5 | 5 | 0.0160 | 5 | 4 | 0.0043 | 5 | 5 | 0.0099 | 4 | 5 | 0.9935 |
| Neutrophils | 5 | 5 | <0.0001 | 5 | 4 | <0.0001 | 5 | 5 | <0.0001 | 4 | 5 | 0.8219 |

***Figure S5:*** *IL-8 protein secretion from THP-1 cells.* Ordinary one-way ANOVA performed individually per timepoint against the TNF-α + DMSO treated group, 5 comparisons per family. ($F = 64.43$ for 2 h, 24.98 for 4 h and 111.2 for 6 h). Showing p-values adjusted using Dunnett's multiple comparisons test. Data from a single experimental day.

| | **2h** | | **4h** | | **6h** | |
|---|---|---|---|---|---|---|
| | **n** | **p** | **n** | **p** | **n** | **p** |
| no TNF-α | 3 | <0.0001 | 2 | <0.0001 | 3 | <0.0001 |
| DMSO | 2 | N/A | 3 | N/A | 3 | N/A |
| GNE | 3 | 0.0066 | 3 | 0.0289 | 3 | 0.0033 |
| **2** | 3 | 0.0219 | 3 | 0.0565 | 3 | 0.0006 |
| **5** | 3 | >0.9999 | 3 | 0.8858 | 3 | 0.1233 |
| A485 | 3 | <0.0001 | 3 | <0.0001 | 3 | <0.0001 |

***Figure S7b***: *Cytokine levels in the pLN post rmTNF-α administration.* Ordinary one-way ANOVA performed individually per each tested concentration of TNF- α against the control PBS group, 4 comparisons per family [PBS vs 20ng/50ng/100ng/300ng of TNF-α]. ($F = 19.06$ for IL-α, 10.26 for MCP-1, 4.435 for IL1β, 5.279 for IL-6, 63.19 for TNF- α and 27.16 for IL-17A). Showing p-values adjusted using Dunnett's multiple comparisons test. Data from two biologically independent experiments on different days.

| **Cytokine** | **PBS** | | **20 ng** | | **50 ng** | | **100 ng** | | **300 ng** | |
|---|---|---|---|---|---|---|---|---|---|---|
| | **n** | **p** | **n** | **p** | **n** | **p** | **n** | **p** | **n** | **p** |

| | | | | | | | | | | |
|---|---|---|---|---|---|---|---|---|---|---|
| IL-1α | 4 | N/A | 4 | 0.9993 | 5 | 0.3119 | 4 | 0.0745 | 4 | <0.0001 |
| IL-1β | 4 | N/A | 4 | 0.8029 | 5 | 0.9378 | 4 | 0.8767 | 4 | 0.0713 |
| MCP-1 | 4 | N/A | 4 | 0.9282 | 5 | 0.0623 | 4 | 0.0148 | 4 | 0.0001 |
| TNF-α | 4 | N/A | 4 | 0.9993 | 5 | 0.1149 | 4 | 0.0040 | 4 | <0.0001 |
| IL-6 | 4 | N/A | 4 | >0.9999 | 5 | 0.9559 | 4 | 0.0629 | 4 | 0.0102 |
| IL17a | 4 | N/A | 4 | 0.9982 | 5 | 0.1239 | 4 | 0.0009 | 4 | <0.0001 |

***Figure S7c-e****: Absolute counts of B cells (B220+), CD11b+ DC and CD11B-DC in the pLN at 5h post-rmTNF-α administration.* Ordinary one-way ANOVA performed individually per cell type, 6 comparisons per family [PBS + Cap vs. TNF-α + Cap / GNE272 / **2**, TNF-α + Cap vs. TNF-α + GNE272 / **2** and TNF-α + GNE272 vs. TNF-α + **2**] (F = 5.266 for B220, 4.930 for CD11B+ DC, 6.938 for CD11B- DC). Showing p-values adjusted using Šídák's multiple comparisons test. Data from two biologically independent experiments on different days.

| **Cells** | **TNF-α + Cap vs.** | | | | | | | | | **TNF-α + GNE272 vs. TNF-α + 2** | | |
|---|---|---|---|---|---|---|---|---|---|---|---|---|
| | **PBS + Cap** | | | **TNF-α + GNE272** | | | **TNF-α + 2** | | | | | |
| | **n1** | **n2** | **p** | **n1** | **n2** | **p** | **n1** | **n2** | **p** | **n1** | **n2** | **p** |
| B220+ | 5 | 5 | 0.0170 | 5 | 4 | 0.0110 | 5 | 5 | 0.0181 | 4 | 5 | 0.9990 |
| CD11b+ DC | 5 | 5 | 0.0494 | 5 | 4 | 0.0071 | 5 | 5 | 0.0130 | 4 | 5 | 0.9980 |
| CD11b- DC | 5 | 5 | 0.0035 | 5 | 5 | 0.0005 | 5 | 5 | 0.0100 | 5 | 5 | 0.7540 |

# Synthetic Experimental Procedures, Schemes and Compound Characterisation

## General Experimental Procedures

### Characterization of compounds

NMR spectra were recorded on AV 300, AV2 400 or AV2 500 MHz Bruker spectrometers. The spectra are calibrated to the residual $^{1}$H and $^{13}$C signals of the solvents. Chemical shifts are reported in ppm and the spectra are calibrated using the residual chloroform signals (7.26 ppm for $^{1}$H NMR and 77.16 ppm for $^{13}$C NMR), the residual DMSO signals (2.50 ppm for $^{1}$H NMR and 39.52 ppm for $^{13}$C NMR), the residual methanol signals (3.31 ppm for $^{1}$H NMR and 49.00 ppm for $^{13}$C NMR), the residual acetone signals (2.05 ppm for $^{1}$H NMR and 29.84 ppm for $^{13}$C NMR) and the residual dichloromethane signals (5.32 ppm for $^{1}$H NMR and 53.84 ppm for $^{13}$C NMR). Multiplicities are abbreviated as follows: singlet (s), doublet (d), triplet (t), quartet (q), doublet-doublet (dd), quintet (quint), multiplet (m), and broad (br). High resolution electrospray ionization mass spectrometry (HRMS (ESI)): Dionex Ultimate 3000 UHPLC system (ThermoFischer Scientifics, Germering, Germany) connected to a QExactive MS with a heated ESI source (ThermoFisher Scientific, Bremen, Germany); mass calibration to <2 ppm accuracy with Pierce® ESI calibration solns. (ThermoFisher Scientific, Rockford, USA). The enantiomeric ratios were determined by chiral HPLC analysis performed on JASCO HPLC system equipped with a PU-980 pump, a UV-970 detector, measured at 254 nm and a chiral column OD-H.

### Synthetic and purification methods

Reactions were carried out under ambient atmosphere unless anhydrous conditions are explicitly mentioned, in this case, reactions were carried out under nitrogen atmosphere using standard Schlenk-techniques. All reagents were used as received unless otherwise noted. Solvents were purchased in the best quality available. For anhydrous solvents, the solvents were purged with argon and passed through alumina columns in a solvent purification system (Innovative Technology). Reactions were monitored by thin layer chromatography (TLC) using Merck TLC silica gel 60 $F_{254}$ and using UV light (254 nm) as a visualizing agent and acidic ceric ammonium molybdate/ phosphomolybdic acid, potassium permanganate or vanillin solutions with heat as developing agents. Column chromatography was performed over silica gel (230-400 mesh) under nitrogen or air pressure.

### Synthesis of compound 1

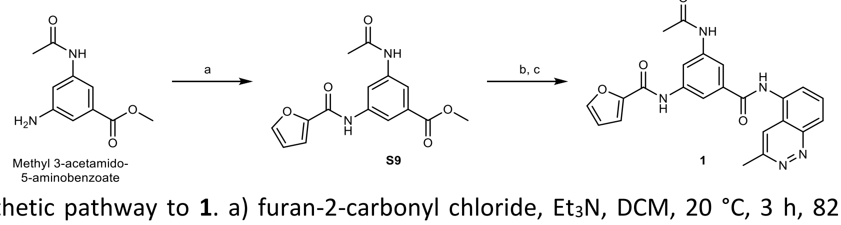


**Scheme S1:** Synthetic pathway to **1**. a) furan-2-carbonyl chloride, $Et_3N$, DCM, 20 °C, 3 h, 82 %; b) $LiOH{\cdot}H_2O$, THF/MeOH/$H_2O$ (2:1:1), 20 °C, 26 h; c) HATU, DIPEA, DMF, 20 °C, 2 h, then 3-methylcinnolin-5-amine, 70 °C, 3 days, 32 % over 2 steps.

**Compound S9**

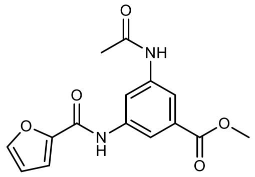


Under anhydrous conditions, methyl 3-acetamido-5-aminobenzoate[21] (900 mg, 4.32 mmol) was suspended in dry DCM (50 mL). $Et_3N$ (783 µL, 5.62 mmol) and furan-2-carbonyl chloride (426 µL, 4.32 mmol) were added. The reaction was allowed to stir at 20 °C for 3 h, upon which the reaction mixture was concentrated and purified by flash column chromatography (EtOAc:MeOH 100:0 to 80:20) to obtain compound **S9** as pale yellow solid (1.07 g, 3.54 mmol, 82 % yield). $^1$H NMR (400 MHz, DMSO-*d6*) δ = 10.39 (s, 1H), 10.19 (s, 1H), 8.35 (t, *J* = 2.0 Hz, 1H), 8.06 (t, *J* = 1.8 Hz, 1H), 8.01 (t, *J* = 1.8 Hz, 1H), 7.95 (dd, *J* = 1.7, 0.8 Hz, 1H), 7.40 (dd, *J* = 3.5, 0.8 Hz, 1H), 6.70 (dd, *J* = 3.5, 1.7 Hz, 1H), 3.86 (s, 3H), 2.06 (s, 3H); $^{13}$C NMR (101 MHz, DMSO-*d6*) δ = 168.6, 166.0, 156.4, 147.2, 146.0, 139.9, 139.2, 130.2, 115.7, 115.0 (2C), 114.9, 112.1, 52.2, 24.0 ppm; HRMS (ESI) m/z calcd for $C_{15}H_{15}O_5N_2^+$ $[M+H]^+$ = 303.0976, found = 303.0976.

**Compound 1**

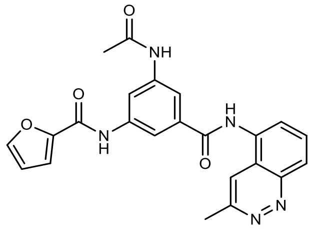


**S9** (517 mg, 1.71 mmol) was suspended in THF:MeOH:$H_2O$ (2:1:1, 6.8 mL), lithium hydroxide monohydrate (215 mg, 5.13 mmol) was added, and the mixture was stirred at 20 °C for 26 h. The organic solvents were removed under reduced pressure, and the aqueous residue was acidified with HCl (1 M) to pH 1. The precipitate was filtered and washed with $H_2O$ to obtain the carboxylic acid as a pale brown solid (375 mg), which was used without further purification in the next step. To a stirred solution of the carboxylic acid (28.8 mg, 0.1 mmol) in dry DMF (0.5 mL), HATU (45.6 mg, 0.12 mmol) and dry DIPEA (29 µL, 0.3 mmol) were added. The solution was allowed to stir at 20 °C for 2 h, upon which 3-methylcinnolin-5-amine (31.8 mg, 0.2 mmol) was added. The reaction was heated to 70 °C and stirred for 3 days. The reaction mixture is concentrated under reduced pressure. The residue was dissolved in EtOAc and washed with $NaHCO_3$ solution (sat.). The organic layer was dried over $MgSO_4$, filtered and concentrated under reduced pressure. The crude material was purified by flash column chromatography (neutral aluminium oxide, DCM:MeOH = 98:2) to yield compound **1** as a brown solid (18 mg, 0.042 mmol, 32 % yield over 2 steps). $^1$H NMR (500 MHz, DMSO-$d_6$) δ = 10.64 (s, 1H), 10.40 (s, 1H), 10.21 (s, 1H), 8.36 – 8.30 (m, 2H), 8.09 – 8.05 (m, 1H), 8.02 – 7.87 (m, 5H), 7.42 (d, *J* = 3.5 Hz, 1H), 6.72 (dd, *J* = 3.5, 1.8 Hz, 1H), 2.89 (s, 3H), 2.10 (s, 3H); $^{13}$C NMR (126 MHz, DMSO-$d_6$) δ = 168.6, 166.7, 156.4, 153.2, 149.1, 147.3, 146.0, 139.7, 139.0, 135.3, 133.2, 129.7, 126.7, 126.6, 122.2, 117.5, 115.0 (2C), 114.3, 114.3, 112.2, 24.1, 21.8; HRMS (ESI), m/z: $[M+Na]^+$ calcd for $C_{23}H_{19}O_4N_5Na^+$: 452.1329 found: 452.1331.

## Synthesis of compounds 2 and 3

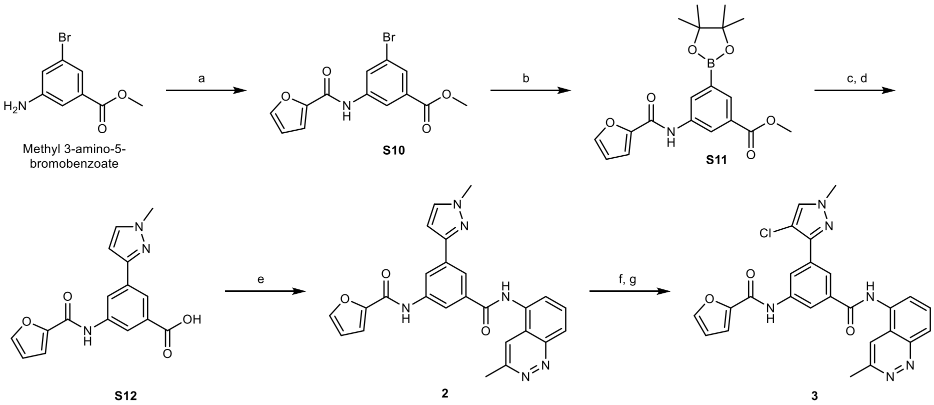


**Scheme S2:** Synthetic pathway to **2** and **3**. a) 2-furoic acid, EDC·HCl, HOBt·$H_2O$, DCM, 25 °C, 15 h, 67 %; b) $B_2Pin_2$, Pd(dppf)$Cl_2$, KOAc, 1,4-dioxane, 100 °C, 16 h, 77 %; c) 3-iodo-1-methyl-1*H*-pyrazole, Pd(dppf)$Cl_2$, $K_2CO_3$, 1,4-dioxane/$H_2O$ (20:1), 90 °C, 16 h; d) LiOH·$H_2O$, THF/MeOH/$H_2O$ (2:1:2), 20 °C, 2 h, 59 % over 2 steps; e) HATU, DIPEA, DMF, 25 °C, 90 min, then 3-methylcinnolin-5-amine, 70 °C, 4 days, 35 %; f) HCl, toluene/$Et_2O$ (1:3), 25 °C, 5 min; g) 4,5-dichlorophthaloyl peroxide, HFIP, 70 °C, 48 h, 33% over 2 steps.

## Compound S10:

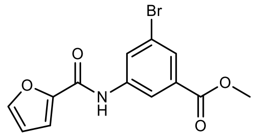

To a stirred solution of methyl 3-amino-5-bromobenzoate (5 g, 21.7 mmol) and 2-furoic acid (2.4 g, 21.7 mmol) in DCM (72 mL), EDC·HCl (8.3 g, 43.4 mmol) and HOBt·$H_2O$ (1.47 g, 9.6 mmol) were added. The reaction mixture was stirred at 25 °C for 15 h and concentrated under reduced pressure. The obtained residue was triturated in water and the resulting suspension was filtered, washed with water, with $NaHCO_3$ (sat.), with HCl (1 M) and dried under vacuum to afford the desired product **S10** as a beige solid (4.7 g, 67 % yield). $^1H$ NMR (400 MHz, DMSO-*d6*) δ = 10.55 (s, 1H), 8.41 (dd, *J* = 2.0, 1.5 Hz, 1H), 8.34 (t, *J* = 2.0 Hz, 1H), 7.99 (dd, *J* = 1.7, 0.8 Hz, 1H), 7.77 (dd, *J* = 1.9, 1.4 Hz, 1H), 7.40 (dd, *J* = 3.5, 0.8 Hz, 1H), 6.73 (dd, *J* = 3.5, 1.7 Hz, 1H), 3.88 (s, 3H); $^{13}C$ NMR (101 MHz, DMSO-*d6*) δ = 164.8, 156.4, 146.8, 146.3, 140.6, 131.9, 126.5, 126.3, 121.6, 119.5, 115.6, 112.3, 52.6; HRMS (ESI), m/z: $[M+Na]^+$ calcd for $C_{13}H_{10}BrNNaO_4^+$: 345.9691 found: 345.9688.

## Compound S11:

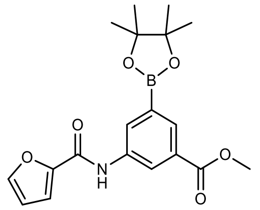

To a stirred solution of **S10** (4.7 g, 14.5 mmol) in dry 1,4-dioxane (48 mL) under a nitrogen atmosphere, $B_2Pin_2$ (4 g, 16 mmol), KOAc (5.7 g, 58 mmol) and Pd(dppf)$Cl_2$ (1.06 mg, 1.45 mmol) were added. The reaction mixture was heated at 100 °C for 16 h and concentrated under reduced pressure. The obtained residue was filtered through a short pad of celite, concentrated under reduced pressure and purified by flash column chromatography (EtOAc/hexane = 3:7 to 5:5) The fractions containing the product were combined, concentrated under reduced pressure, triturated in hexane/$Et_2O$ = 7:3 and the resulting

suspension was filtered, washed with hexane and dried under vacuum to afford the desired product **S11** as a white solid (4.15 g, 77 % yield). $^{1}$H NMR (400 MHz, $CDCl_3$) δ = 8.53 (dd, *J* = 2.3, 1.6 Hz, 1H), 8.25 (dd, *J* = 1.6, 1.0 Hz, 1H), 8.16 – 8.12 (m, 2H), 7.52 (dd, *J* = 1.8, 0.8 Hz, 1H), 7.26 – 7.25 (m, 1H), 6.57 (dd, *J* = 3.5, 1.8 Hz, 1H), 3.92 (s, 3H), 1.36 (s, 12H); $^{13}$C NMR (126 MHz, $CDCl_3$) δ = 166.8, 156.2, 147.7, 144.5 (2C), 137.3, 131.9, 130.8, 130.3, 123.7, 115.7, 112.8, 84.4 (2C), 52.3, 25.0 (4C); HRMS (ESI), m/z: $[M+H]^+$ calcd for $C_{19}H_{23}BNO_6^+$: 372.1613 found: 372.1618.

**Compound S12:**

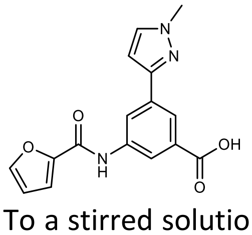

To a stirred solution of **S11** (2 g, 5.4 mmol) in 1,4-dioxane (20 mL) and water (1 mL) under a nitrogen atmosphere, 3-iodo-1-methyl-1*H*-pyrazole (520 µL, 4.8 mmol), $K_2CO_3$ (2.2 g, 16.2 mmol) and Pd(dppf)$Cl_2$ (395 mg, 0.54 mmol) were added. Nitrogen gas was bubbled through the reaction for five minutes and the reaction mixture was stirred at 90 °C for 16 h. It was then concentrated under reduced pressure and purified by flash column chromatography (EtOAc/toluene = 2:8 to 3:7) to afford the impure desired product which was engaged in the next step without further purification. To a stirred solution of the above crude in two batches (790 / 882 mg, 2.43 / 2.71 mmol) in a mixture of tetrahydrofuran (2.4 / 2.7 mL), methanol (1.2 / 1.35 mL) and water (2.4 / 2.7 mL), lithium hydroxide hydrate (306 / 341 mg, 7.29 / 8.13 mmol) was added. The mixtures were stirred at 20 °C for 2 h. HCl (1 M) was added until the formation of a precipitate. The resulting suspensions were combined and filtered, washed with water, and dried under vacuum to afford the desired product **S12** as a white solid (670 mg, 59 % yield over two steps). $^{1}$H NMR (300 MHz, DMSO-$d_6$) δ = 13.09 (s, 1H), 10.40 (s, 1H), 8.43 (s, 1H), 8.35 (s, 1H), 8.05 (s, 1H), 7.97 (s, 1H), 7.78 (d, *J* = 2.2 Hz, 1H), 7.41 (d, *J* = 3.6 Hz, 1H), 6.76 – 6.68 (m, 1H), 6.69 (d, *J* = 2.3 Hz, 1H), 3.91 (s, 3H); $^{13}$C NMR (101 MHz, DMSO-$d_6$) δ = 167.1, 156.4, 149.0, 147.2, 146.0, 139.2, 134.1, 132.7, 131.7, 121.0, 120.6, 119.8, 114.9, 112.2, 102.6, 38.7; HRMS (ESI), m/z: $[M+H]^+$ calcd for $C_{16}H_{14}N_3O_4^+$: 312.0979 found: 312.0984.

**Compound 2:**

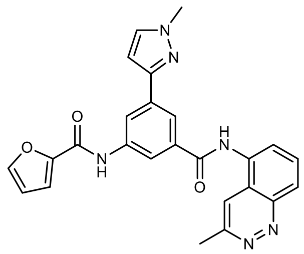

To a stirred solution of **S12** (31 mg, 0.1 mmol) in dry DMF (1 mL), HATU (45.6 mg, 0.12 mmol) and dry DIPEA (52 µL, 0.54 mmol) were added. The reaction was stirred at 25 °C for 90 min. Then 3-methylcinnolin-5-amine (31.8 mg, 0.2 mmol) was added. The reaction was heated to 70 °C and stirred for 4 days. The reaction was quenched with water and then concentrated under reduced pressure. The residue was dissolved in EtOAc and washed with $NaHCO_3$ (sat.). The organic phase was dried with $MgSO_4$, filtered and dried under reduced pressure. The crude material was triturated with ice-cold MeOH and filtered to afford **2** as an off-white solid

(16 mg, 0.035 mmol, 35 % yield). $^{1}H$ NMR (400 MHz, DMSO-$d_6$) δ = 10.72 (s, 1H), 10.42 (s, 1H), 8.49 (t, *J* = 1.8 Hz, 1H), 8.39 – 8.32 (m, 2H), 8.23 (t, *J* = 1.6 Hz, 1H), 8.07 – 8.04 (m, 1H), 7.99 – 7.90 (m, 3H), 7.81 (d, *J* = 2.3 Hz, 1H), 7.43 (dd, *J* = 3.5, 0.9 Hz, 1H), 6.78 (d, *J* = 2.3 Hz, 1H), 6.73 (dd, *J* = 3.5, 1.8 Hz, 1H), 3.93 (s, 3H), 2.89 (s, 3H); $^{13}C$ NMR (126 MHz, DMSO-$d_6$) δ = 166.4, 156.4, 153.3, 149.3, 149.1, 147.3, 146.0, 139.2, 135.1, 134.1, 133.3, 132.6, 129.7, 127.1, 126.7, 122.5, 119.8, 119.8, 119.0, 117.7, 115.0, 112.2, 102.9, 38.8, 21.7; HRMS (ESI), m/z: $[M+H]^+$ calcd for $C_{25}H_{21}N_6O_3^+$: 453.1670 found: 453.1671.

**Compound 3:**

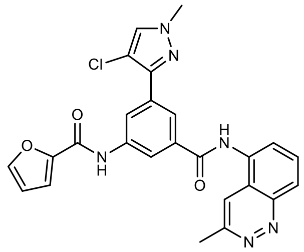


Under anhydrous conditions, HCl (2 M in anhydrous $Et_2O$) was diluted to 0.5 M with toluene. **2** (3.1 mg, 0.01 mmol) was added, and the bright yellow solution was stirred at 25 °C for 5 min. The volatiles were thoroughly removed. To the residue 4,5-dichlorophthaloyl peroxide (5.8 mg, 0.025 mmol) was added, the atmosphere was flushed with $N_2$, sealed and the solids were suspended in dry HFIP (0.2 mL). The suspension was stirred at 70 °C for 48 h. The volatiles were removed with a flow of $N_2$ and to the remaining residue, under anhydrous conditions, a mixture of deoxygenated MeOH/$NaHCO_3$ (sat.) (9:1, 0.1 mL) was added and the mixture was stirred at 50 °C for 1 h. The volatiles were again removed, and the residue was partitioned between EtOAc and aqueous phosphate buffer (pH = 7). The aqueous phase was extracted with EtOAc, and the combined organic layers were dried over $MgSO_4$, filtered, and concentrated under reduced pressure. The crude material was passed through a short silica plug and then purified by flash chromatography (EtOAc/methanol = 95:5) to obtain **3** as a white solid (1.6 mg, 0.0032 mmol, 33 %). $^{1}H$ NMR (500 MHz, DMSO-*d6*) δ = 10.75 (s, 1H), 10.52 (s, 1H), 8.59 (t, *J* = 1.8 Hz, 1H), 8.42 (t, *J* = 1.9 Hz, 1H), 8.35 (dt, *J* = 8.3, 1.2 Hz, 1H), 8.25 (t, *J* = 1.6 Hz, 1H), 8.12 (s, 1H), 8.03 (s, 1H), 7.98 – 7.97 (m, 1H), 7.97 – 7.89 (m, 2H), 7.43 (dd, *J* = 3.5, 0.8 Hz, 1H), 6.73 (dd, *J* = 3.5, 1.7 Hz, 1H), 3.92 (s, 3H), 2.89 (s, 3H); $^{13}C$ NMR (126 MHz, DMSO-*d6*) δ = 166.4, 156.5, 153.3, 149.1, 147.2, 146.1, 144.5, 139.1, 135.2, 133.2, 132.1, 131.3, 129.7, 126.9, 126.7, 122.3, 121.5, 121.3, 119.5, 117.6, 115.1, 112.2, 105.9, 21.7 One signal overlapping with the solvent; HRMS (ESI), m/z: $[M+H]^+$ calcd for $C_{25}H_{20}N_6O_3Cl^+$: 487.1280 found: 487.1282.

## Synthesis of compound 4

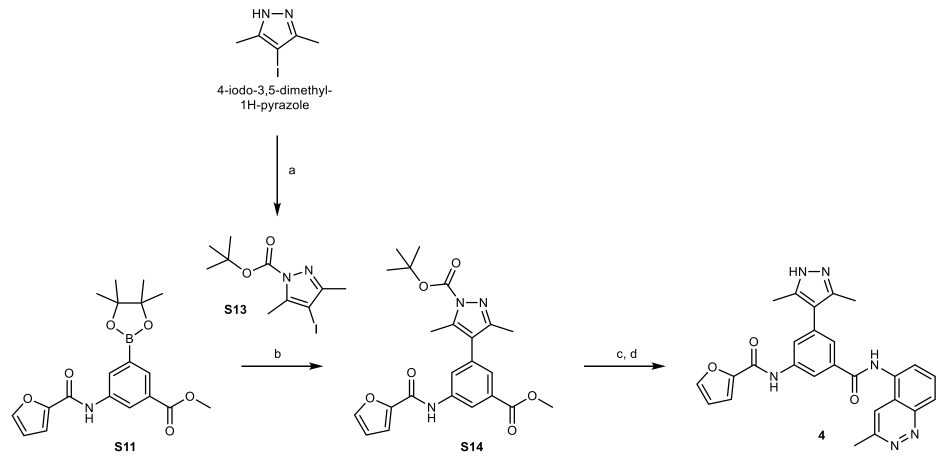


**Scheme S3**: Synthetic pathway to **4**. a) $Boc_2O$, DMAP, DCM, 20 °C, 1h, 98 %; b) $Pd_2(dba)_3$, Xphos, $Cs_2CO_3$, 1,4-dioxane/$H_2O$ (9:1), 20 °C, 18 h, 8 %; c) LiOH·$H_2O$, THF/$H_2O$ (1:1), 25 °C, 16 h; d) HATU, DIPEA, 3-methylcinnolin-5-amine, DMF, 70 °C, 18 h, 3 % over 2 steps.

### Compound S13:

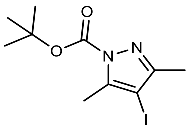

To a solution of 4-iodo-3,5-dimethyl-1H-pyrazole[22] (10.58 g, 47.6 mmol) in DCM (159 mL) was added di-*tert*-butyl dicarbonate (10.4 g, 47.6 mmol) and DMAP (1.16 g, 9.52 mmol). The solution was stirred at 20 °C for 1h, washed with HCl (1 M) and brine, dried over MgSO4, and concentrated under reduced pressure. The crude material was purified by flash column chromatography (EtOAc/hexane = 1:9) to afford **S13** as a colourless solid (14.96 g, 46.4 mmol, 98 %). $^1$H NMR (400 MHz, $CDCl_3$) δ = 2.55 (s, 3H), 2.27 (s, 3H), 1.64 (s, 9H); $^{13}$C NMR (101 MHz, $CDCl_3$) δ = 153.5, 148.1, 145.0, 85.5, 71.1, 28.2 (3C), 15.7, 14.7. HRMS (ESI), m/z: $[M+Na]^+$ calcd for $C_{10}H_{15}N_2INaO_2^+$: 345.0076 found: 345.0074.

### Compound S14:

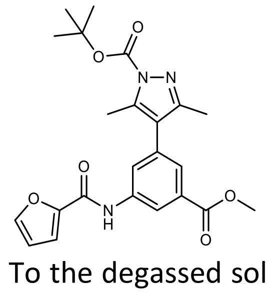

To the degassed solids of **S11** (87 mg, 0.234 mmol), **S13** (113 mg, 0.352 mmol), $Cs_2CO_3$ (153 mg, 0.469 mmol), $Pd_2(dba)_3$ (8.6 mg, 0.0094 mmol) and Xphos (13.4 mg, 0.0281 mmol) was added degassed 1,4-dioxane/$H_2O$ (9:1, 12 mL) and the reaction mixture was stirred at 20 °C for 18 h. The reaction mixture was concentrated under reduced pressure and purified by flash column chromatography (EtOAc/hexane = 1:9 to 1:1 and DCM/MeOH 100:0 to 97.5:2.5) to afford the still impure desired product, which was then further purified by preparative TLC (EtOAc:hexane = 1:1 and DCM:MeOH = 97:3) to afford **S14** as a colourless solid (8.0 mg, 0.234 mmol, 8 %). $^1$H NMR (500 MHz, Acetone-$d_6$) δ = 9.74 (s, 1H), 8.52 (t, $J$ = 1.8 Hz, 1H), 8.08 (t, $J$ = 1.9 Hz, 1H), 7.79 (dd, $J$ = 1.8, 0.8 Hz, 1H), 7.67 (t, $J$ = 1.6 Hz, 1H), 7.28 (dd, $J$ = 3.5, 0.8 Hz, 1H),

6.68 (dd, $J$ = 3.5, 1.8 Hz, 1H), 3.92 (s, 3H), 2.50 (s, 3H), 2.21 (s, 3H), 1.64 (s, 9H); $^{13}$C NMR (126 MHz, Acetone-$d_6$) δ 166.9, 157.3, 150.2, 149.6, 148.9, 146.2, 141.2, 140.3, 134.3, 132.1, 126.5, 126.4, 123.2, 120.5, 115.9, 113.2, 84.8, 52.6, 28.1 (3C), 13.4, 12.9; HRMS (ESI), m/z: [M+H]$^+$ calcd for $C_{23}H_{26}N_3O_6^+$: 440.1816 found: 440.1820.

**Compound 4:**

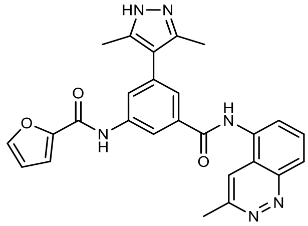

To **S14** (120 mg, 0.27 mmol) was added THF (0.5 mL), $H_2O$ (0.5 mL) and lithium hydroxide monohydrate (34 mg, 0.81 mmol). The reaction was stirred at 25 °C until completion. THF was removed under reduced pressure, the aqueous solution was acidified with HCl (1 M) to pH 1, and the formed precipitate was filtered and dried thoroughly to afford the crude carboxylic acid. The material was purified by flash column chromatography (EtOAc:MeOH:AcOH 100:0:1 to 100:2:1) to obtain a still impure carboxylic acid (~0.27 mmol) which was used in the next step without further purification. To a stirred solution of the carboxylic acid (25 mg, ~ 0.075 mmol) in dry DMF (2 mL), HATU (31 mg, 0.0825 mmol) and dry DIPEA (39 µL, 0.225 mmol) and 3-methylcinnolin-5-amine (30 mg, 0.188 mmol) were added. The reaction was heated to 70 °C and stirred for 18 h. The reaction mixture was concentrated under reduced pressure and purified by flash column chromatography (EtOAc:MeOH = 95:5) to afford **4** as a yellow powder (1.1 mg, 0.0024 mmol, 3 % yield over 2 steps). $^1$H NMR (400 MHz, DMSO) δ = 12.40 (s, 1H), 10.59 (s, 1H), 10.45 (s, 1H), 8.35 (dt, $J$ = 8.3, 1.2 Hz, 1H), 8.32 (t, $J$ = 1.8 Hz, 1H), 8.04 (s, 1H), 7.99 (t, $J$ = 1.8 Hz, 1H), 7.98 – 7.89 (m, 3H), 7.76 (t, $J$ = 1.6 Hz, 1H), 7.40 (dd, $J$ = 3.5, 0.8 Hz, 1H), 6.73 (dd, $J$ = 3.5, 1.7 Hz, 1H), 2.88 (s, 3H), 2.30 (br s, 6H); $^{13}$C NMR (126 MHz, DMSO-$d_6$) δ = 166.4, 156.4, 153.3, 149.1, 147.4, 145.9, 139.0, 134.9, 134.7, 133.2, 129.6, 127.0, 126.6, 123.8, 123.0, 122.4, 117.6 (2C), 116.2, 115.1, 112.3, 21.7, 12.2 (br, 2C); 3 quaternary C peaks missing due to slow rotation; HRMS (ESI): m/z: [M+H]$^+$ calcd for $C_{26}H_{23}N_6O_3^+$: 467.1826 found: 467.1827.

**Synthesis of intermediate S18**

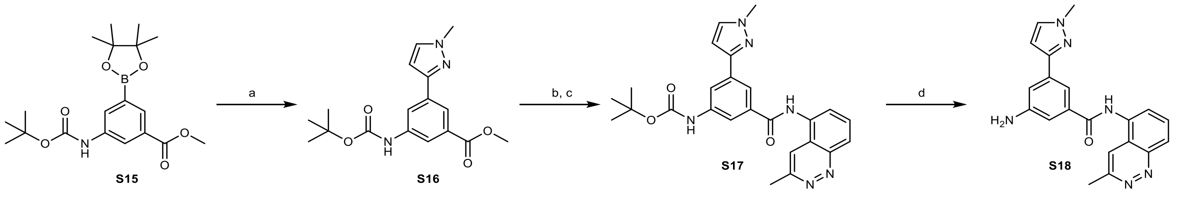


**Scheme S4:** Synthetic pathway to key intermediate **S18**. a) 3-iodo-1-methyl-1$H$-pyrazole, $Pd_2(dba)_3$, XPhos, $Cs_2CO_3$, $H_2O$, 1,4-dioxane, 80 24 h, 67 %; b) LiOH·$H_2O$, THF/$H_2O$ (1:1), 25 °C, 6 h; c) HATU, DIPEA, NMP, 25 °C, 1 h, then 3-methylcinnolin-5-amine, 80 °C, 22 h, 48 % over 2 steps; d) $SiO_2$, 180 °C, 15 min

**Compound S16:**

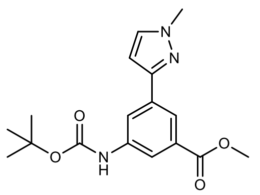

To the degassed solids of methyl 3-((tert-butoxycarbonyl)amino)-5-(4,4,5,5-tetramethyl-1,3,2-dioxaborolan-2-yl)benzoate **S15**[23] (453 mg, 1.20 mmol), $Cs_2CO_3$ (782 mg, 2.40 mmol), $Pd_2(dba)_3$ (54.9 mg, 0.060 mmol) and XPhos (68.6 mg, 0.144 mmol) was added 1,4-dioxane (12 mL). The suspension was purged with $N_2$ for 10 min, upon which 3-iodo-1-methyl-1*H*-pyrazole (157 µL, 1.56 mmol) and $H_2O$ (1 drop) are added. The reaction mixture was heated to 80 °C and stirred for 24 h. The reaction mixture was filtered over a plug of celite and concentrated under reduced pressure. The crude material was purified by flash column chromatography (EtOAc:hexane = 1:7 to 1:1) to yield **S16** as a white solid (268 mg, 1.20 mmol, 67 % yield). $^{1}$H NMR (400 MHz, DMSO-$d_6$) δ = 9.61 (s, 1H), 8.14 (t, *J* = 1.8 Hz, 1H), 8.06 (t, *J* = 1.8 Hz, 1H), 7.94 (t, *J* = 1.6 Hz, 1H), 7.75 (d, *J* = 2.2 Hz, 1H), 6.62 (d, *J* = 2.2 Hz, 1H), 3.90 (s, 3H), 3.87 (s, 3H), 1.49 (s, 9H); $^{13}$C NMR (101 MHz, DMSO-$d_6$) δ = 166.1, 152.8, 149.0, 140.3, 134.3, 132.6, 130.5, 119.3, 118.8, 117.4, 102.5, 79.4, 52.2, 38.7, 28.1 (3C). HRMS (ESI), m/z: $[M+Na]^+$ calcd for $C_{17}H_{21}N_3NaO_4^+$: 354.14243 found: 354.14257.

**Compound S17:**

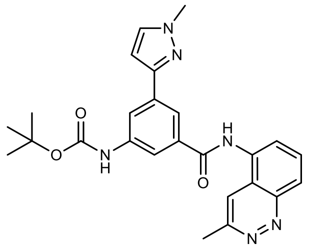

**S16** (1.69 g, 5.10 mmol) was dissolved in THF:$H_2O$ (1:1, 66 mL), lithium hydroxide monohydrate (642 mg, 15.3 mmol) was added and the mixture was stirred at 25 °C for 4.5 h. THF was removed under reduced pressure and the aqueous residue was cooled to 0 °C and acidified with HCl (1 M) to pH 4. The precipitate was filtered dried under vacuum to obtain the carboxylic acid as a white solid (1.0 g, ~3.15 mmol) which was used without further purification in the next step. To a stirred solution of the carboxylic acid (984 mg, ~3.10 mmol) in dry NMP (15.5 mL), HATU (1.414, 3.72 mmol) and dry DIPEA (1.59 mL, 9.3 mmol) were added. The reaction was stirred at 25 °C for 1 h. Then 3-methylcinnolin-5-amine (987 mg, 6.2 mmol) was added. The reaction was heated to 80 °C and stirred for 22 h. The reaction was quenched with ice water and the aqueous phase was extracted using EtOAc. The organic layers were combined, washed with brine, dried over $MgSO_4$, filtered, concentrated under reduced pressure and dried under vacuum at 40 °C for 2 h. The crude material was purified by flash chromatography (EtOAc:MeOH = 98:2) to yield **S17** as a brown solid (1.1 g, 2.40 mmol, 48 % yield over 2 steps). $^{1}$H NMR (400 MHz, $CDCl_3$) δ = 8.48 (s, 1H), 8.40 (dt, *J* = 8.7, 1.0 Hz, 1H), 8.07 (t, *J* = 1.4 Hz, 1H), 8.05 (t, *J* = 1.3 Hz, 1H), 8.01 (d, *J* = 7.4 Hz, 1H), 7.98 (t, *J* = 1.6 Hz, 1H), 7.81 – 7.74 (m, 2H), 7.40 (d, *J* = 2.3 Hz, 1H), 6.80 (s, 1H), 6.62 (d, *J* = 2.3 Hz, 1H), 3.95 (s, 3H), 2.97 – 2.93 (m, 3H), 1.54 (s, 9H); $^{13}$C NMR (101 MHz, $CDCl_3$) δ = 166.5, 154.0, 152.9, 150.3, 149.6, 139.6, 135.3, 135.3, 131.9, 131.7, 129.5, 128.0, 126.1, 122.4, 119.0, 118.9, 116.7,

116.2, 103.6, 81.3, 39.3, 28.5 (3C), 22.4; HRMS (ESI), m/z: $[M+H]^+$ calcd for $C_{25}H_{27}N_6O_3^+$: 459.21392 found: 459.21395.

**Compound S18:**

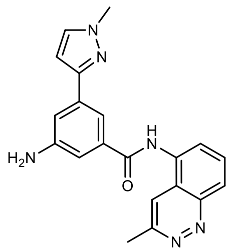

**S17** (66 mg, 0.144 mmol) was dissolved in a minimum amount of DCM. Silica (100 equiv, 865 mg, 14.4 mmol) was added and the mixture was concentrated in vacuo. The dry silica was heated at 180 °C for 15 min, then allowed to cool and suspended in solvent (DCM:MeOH = 9:1), filtered and washed thoroughly with more of the same eluent. The filtrate was dried in vacuo to yield the crude aniline **S18** as a pale yellow-brown solid (48 mg, ~0.134 mmol) which was used in the next steps without further purification.

**Synthesis of compounds 5, 6, 7 and 8**

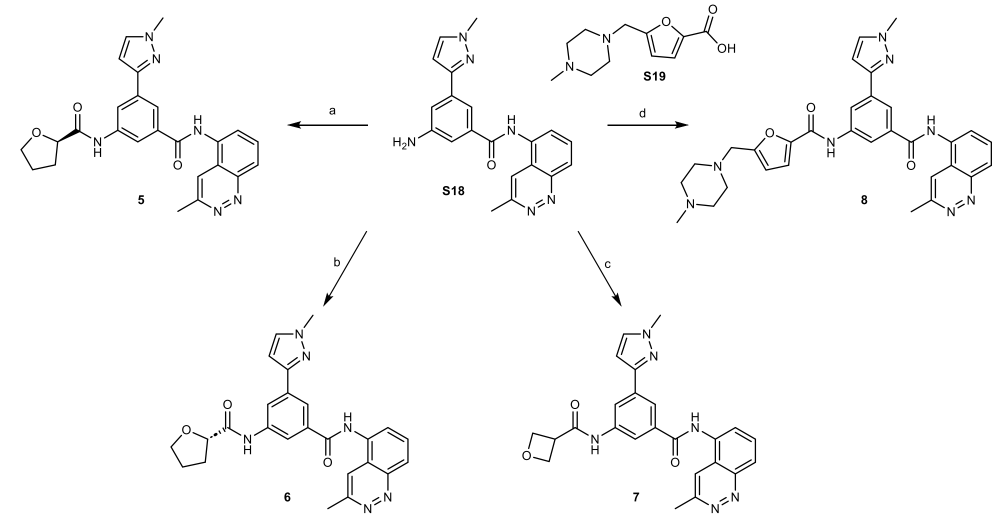

**Scheme S5**: Synthetic pathway to **5**, **6**, **7** and **8**. a) (*R*)-tetrahydrofuran-2-carboxylic acid, EDC·HCl, HOBt·$H_2O$, DMF, 20 °C, 2.5 h, 82 % over 2 steps; b) (*S*)-tetrahydrofuran-2-carboxylic acid, EDC·HCl, HOBt·$H_2O$, DMF, 20 °C, 2.5 h, 73 % over 2 steps; c) oxetane-3-carboxylic acid, EDC·HCl, HOBt·$H_2O$, DMF, 25 °C, 25 h, 33 % over 2 steps; d) carboxylic acid **S19**, EDC·HCl, HOBt·$H_2O$, DMF, 25 °C, 25 h, 42 % over 2 steps;

**Compound 5:**

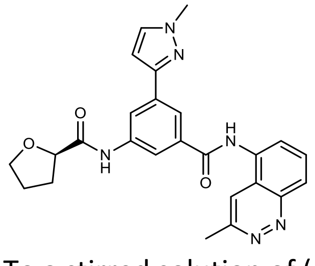

To a stirred solution of (*R*)-tetrahydrofuran-2-carboxylic acid (3.6 mg, 0.031 mmol) in 0.22 mL of DMF, HOBt·$H_2O$ (2.1 mg, 0.014 mmol), EDC·HCl (12 mg, 0.062 mmol) and aniline **S18** (8 mg, ~0.022 mmol) were added. The reaction was stirred at 20 °C for 2.5 h. The reaction mixture

was concentrated under reduced pressure and purified by preparative TLC (DCM:MeOH = 93:7), followed by a trituration with pentane to provide **5** as a pale yellow solid (9.0 mg, 0.020 mmol, 82 % yield over 2 steps from **S18**). $^{1}$H NMR (500 MHz, DMSO-$d_6$) δ = 10.69 (s, 1H), 9.93 (s, 1H), 8.42 (t, *J* = 1.6 Hz, 1H), 8.35 (ddd, *J* = 6.9, 2.7, 1.0 Hz, 1H), 8.27 (t, *J* = 1.6 Hz, 1H), 8.19 (t, *J* = 1.6 Hz, 1H), 8.03 (s, 1H), 7.95 – 7.89 (m, 2H), 7.80 (d, *J* = 2.2 Hz, 1H), 6.75 (d, *J* = 2.2 Hz, 1H), 4.43 (dd, *J* = 8.3, 5.6 Hz, 1H), 4.07 – 3.99 (m, 1H), 3.92 (s, 3H), 3.85 (dt, *J* = 8.1, 6.7 Hz, 1H), 2.89 (s, 3H), 2.29 – 2.18 (m, 1H), 2.07 – 1.97 (m, 1H), 1.96 – 1.82 (m, 2H); $^{13}$C NMR (126 MHz, DMSO-$d_6$) δ = 171.9, 166.4, 153.3, 149.3, 149.1, 139.0, 135.1, 134.1, 133.3, 132.6, 129.6, 127.1, 126.7, 122.5, 119.7, 119.6, 118.7, 117.7, 102.9, 77.9, 68.9, 38.7, 30.1, 25.1, 21.7; HRMS (ESI), m/z: $[M+H]^+$ calcd for $C_{25}H_{25}N_6O_3^+$: 457.19827 found: 457.19824. HPLC conditions: OD-H column, *n*-hexane:iPrOH = 75:25, flow rate = 1.0 mL·min$^{-1}$ , tR = 60.2 min (major), 74.0 min (minor).

**Compound 6:**

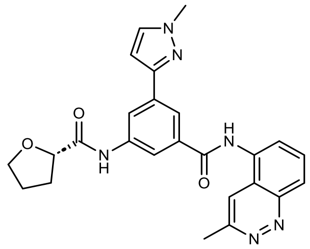

To a stirred solution of (S)-Tetrahydrofuran-2-carboxylic acid (3.6 mg, 0.031 mmol) in 0.22 mL of DMF, HOBt·$H_2O$ (2.1 mg, 0.014 mmol), EDC·HCl (12 mg, 0.062 mmol) and aniline **S18** (8 mg, ~0.022 mmol). The reaction was stirred at 20 °C for 2.5 h. The reaction mixture was concentrated under reduced pressure and purified by preparative TLC (DCM:MeOH = 93:7), followed by a trituration with pentane to provide **6** as a pale yellow solid (8.0 mg, 0.018 mmol, 73 % yield over 2 steps from **S18**). $^{1}$H NMR (500 MHz, DMSO-$d_6$) δ = 10.69 (s, 1H), 9.93 (s, 1H), 8.42 (t, *J* = 1.6 Hz, 1H), 8.35 (ddd, *J* = 6.8, 2.7, 1.0 Hz, 1H), 8.27 (t, *J* = 1.6 Hz, 1H), 8.19 (t, *J* = 1.6 Hz, 1H), 8.03 (s, 1H), 7.94 – 7.89 (m, 2H), 7.80 (d, *J* = 2.2 Hz, 1H), 6.75 (d, *J* = 2.2 Hz, 1H), 4.43 (dd, *J* = 8.3, 5.5 Hz, 1H), 4.07 – 3.99 (m, 1H), 3.92 (s, 3H), 3.85 (dt, *J* = 8.1, 6.7 Hz, 1H), 2.89 (s, 3H), 2.28 – 2.18 (m, 1H), 2.06 – 1.99 (m, 1H), 1.95 – 1.84 (m, 2H); $^{13}$C NMR (126 MHz, DMSO-$d_6$) δ = 171.9, 166.4, 153.3, 149.3, 149.1, 139.0, 135.1, 134.1, 133.3, 132.6, 129.6, 127.1, 126.7, 122.5, 119.7, 119.6, 118.7, 117.7, 102.9, 77.9, 68.9, 38.7, 30.1, 25.1, 21.7; HRMS (ESI), m/z: $[M+H]^+$ calcd for $C_{25}H_{25}N_6O_3^+$: 457.19827 found: 457.19818. HPLC conditions: OD-H column, *n*-hexane:*i*-PrOH = 75:25, flow rate = 1.0 mL·min$^{-1}$ , $t_R$ = 65.2 min (minor), 75.8 min (major).

**Compound 7:**

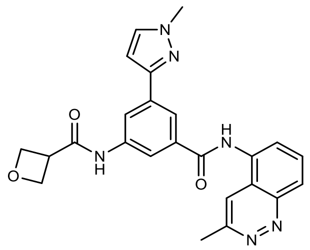

EDC·HCl (23 mg, 0.12 mmol) and HOBt·$H_2O$ (2.0 mg, 0.013 mmol) were added to a stirred solution of aniline **S18** (10.8 mg, ~0.03 mmol) and oxetane-3-carboxylic acid (6.1 mg, 0.06

mmol) in DMF (1 mL). The reaction mixture was stirred at 25 °C for 25 h and concentrated under reduced pressure. The crude was separated by preparative TLC (eluent: DCM:MeOH 94:6). The obtained colourless oil was dissolved in 0.2 mL of DCM/MeOH mixture (1:1) and the desired product was crashed out by the addition of diethyl ether (1.3 mL). Decantation of the solvent afforded **7** as a white solid (4.7 mg, 0.011 mmol, 33 % yield over 2 steps from **S18**). $^{1}$H NMR (500 MHz, MeOD-*d4*) δ = 8.38 (dt, *J* = 8.8, 1.2 Hz, 1H), 8.28 (t, *J* = 1.8 Hz, 1H), 8.24 (t, *J* = 1.5 Hz, 1H), 8.24 – 8.21 (m, 1H), 8.13 (s, 1H), 8.01 (dd, *J* = 7.4, 1.0 Hz, 1H), 7.94 (dd, *J* = 8.5, 7.5 Hz, 1H), 7.67 (d, *J* = 2.3 Hz, 1H), 6.74 (d, *J* = 2.3 Hz, 1H), 4.97 – 4.88 (m, 4H), 4.07 (tt, *J* = 8.4, 6.6 Hz, 1H), 3.97 (s, 3H) , 2.95 (s, 3H); $^{13}$C NMR (126 MHz, MeOD-*d4*) δ = 172.7, 169.5 155.5, 151.7, 150.7, 140.7, 136.5, 136.2, 134.1, 133.8, 131.3, 129.1, 128.2, 125.1, 121.6, 121.6, 120.3, 119.8, 104.3, 74.5 (2C), 41.7, 39.1, 21.9; HRMS (ESI), m/z: $[M+H]^+$ calcd for $C_{24}H_{23}O_3N_6^+$: 443.18262 found: 443.18277.

**Compound 8:**

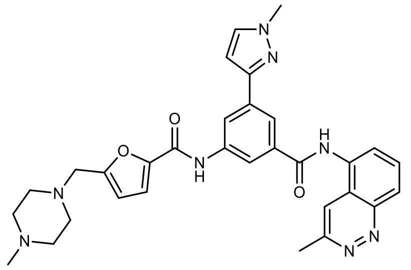

EDC·HCl (23 mg, 0.12 mmol) and HOBt·$H_2O$ (2.0 mg, 0.013 mmol) were added to a stirred solution of aniline **S18** (10.8 mg, ~0.03 mmol) and carboxylic acid **S19**[24] (21.5 mg, 0.06 mmol) in DMF (1 mL). The reaction mixture was stirred at 25 °C for 25 h and concentrated under reduced pressure. The residue was dissolved in MeOH (1 mL) and diluted with DCM (25 mL). The organic layer was washed with saturated $Na_2CO_3$ (20 mL), brine (20 mL), and concentrated under reduced pressure. The crude product was purified by preparative TLC (eluent: acetone:MeOH 2:1). The obtained colourless oil was dissolved in 0.2 mL of DCM/MeOH mixture (1:1) and the desired product was crashed out by the addition of diethyl ether (1.3 mL). Decantation of the solvent afforded **8** as a white solid (7.6 mg, 0.013 mmol, 42 % yield over 2 steps from **S18**). $^{1}$H NMR (500 MHz, $CD_2Cl_2$) δ = 8.59 (s, 1H), 8.49 (s, 1H), 8.41 – 8.35 (m, 2H), 8.29 (s, 1H), 8.14 (s, 1H), 8.06 (d, *J* = 7.3 Hz, 1H), 7.85 – 7.78 (m, 2H), 7.47 (d, *J* = 2.2 Hz, 1H), 7.18 (d, *J* = 3.4 Hz, 1H), 6.69 (d, *J* = 2.2 Hz, 1H), 6.41 (d, *J* = 3.4 Hz, 1H), 3.96 (s, 3H), 3.60 (s, 2H), 2.94 (s, 3H), 2.75 – 2.27 (m, 8H), 2.22 (s, 3H); $^{13}$C NMR (126 MHz, $CD_2Cl_2$) δ = 166.4, 156.7, 155.6, 154.4, 150.1, 149.9, 147.3, 139.3, 135.8, 135.8, 132.3 (2C), 129.6, 128.1, 126.2, 122.6, 120.2, 120.1, 117.7, 116.5, 116.5, 112.0, 103.5, 55.3 (2C), 55.2, 2C hidden under solvent, 46.1, 39.5, 22.5; HRMS (ESI), m/z: $[M+H]^+$ calcd for $C_{31}H_{33}O_3N_8^+$: 565.26702 found: 565.26711.

# $^{1}H$ and $^{13}C$ NMR Spectra and Chiral HPLC Traces

**Compound 1** $^{1}H$ NMR (500 MHz, DMSO-*d6*) and $^{13}C$ NMR (126 MHz, DMSO-$d_6$)

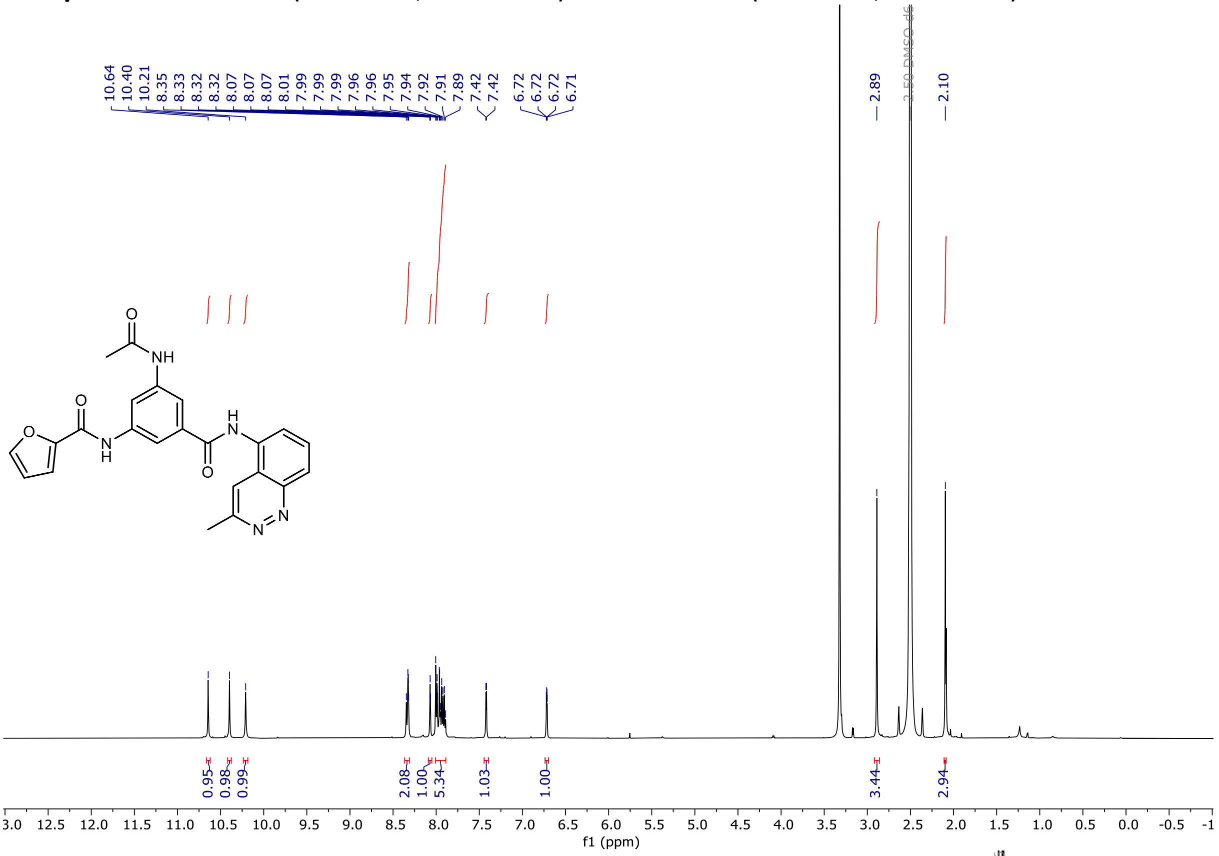


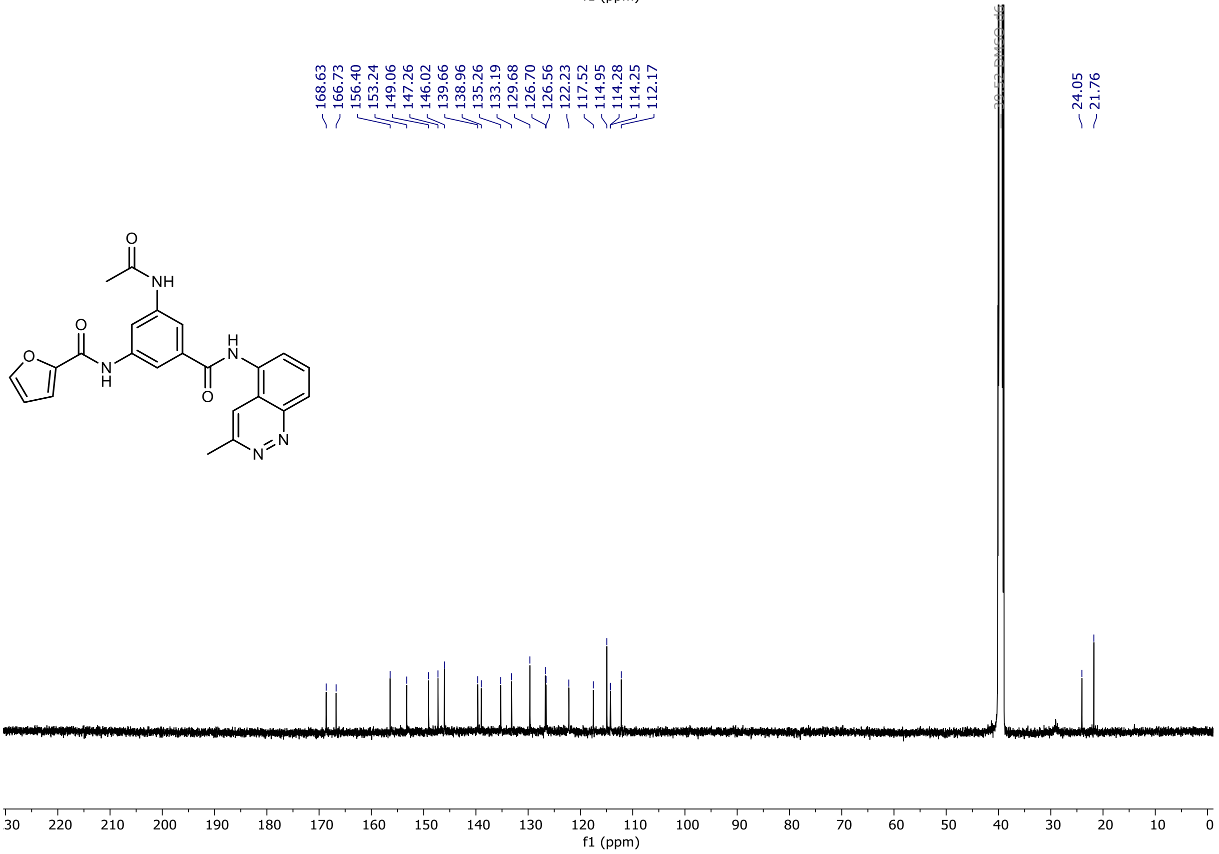

**Compound 2** $^{1}$H NMR (400 MHz, DMSO-*d6*) and $^{13}$C NMR (126 MHz, DMSO-*d*$_6$)

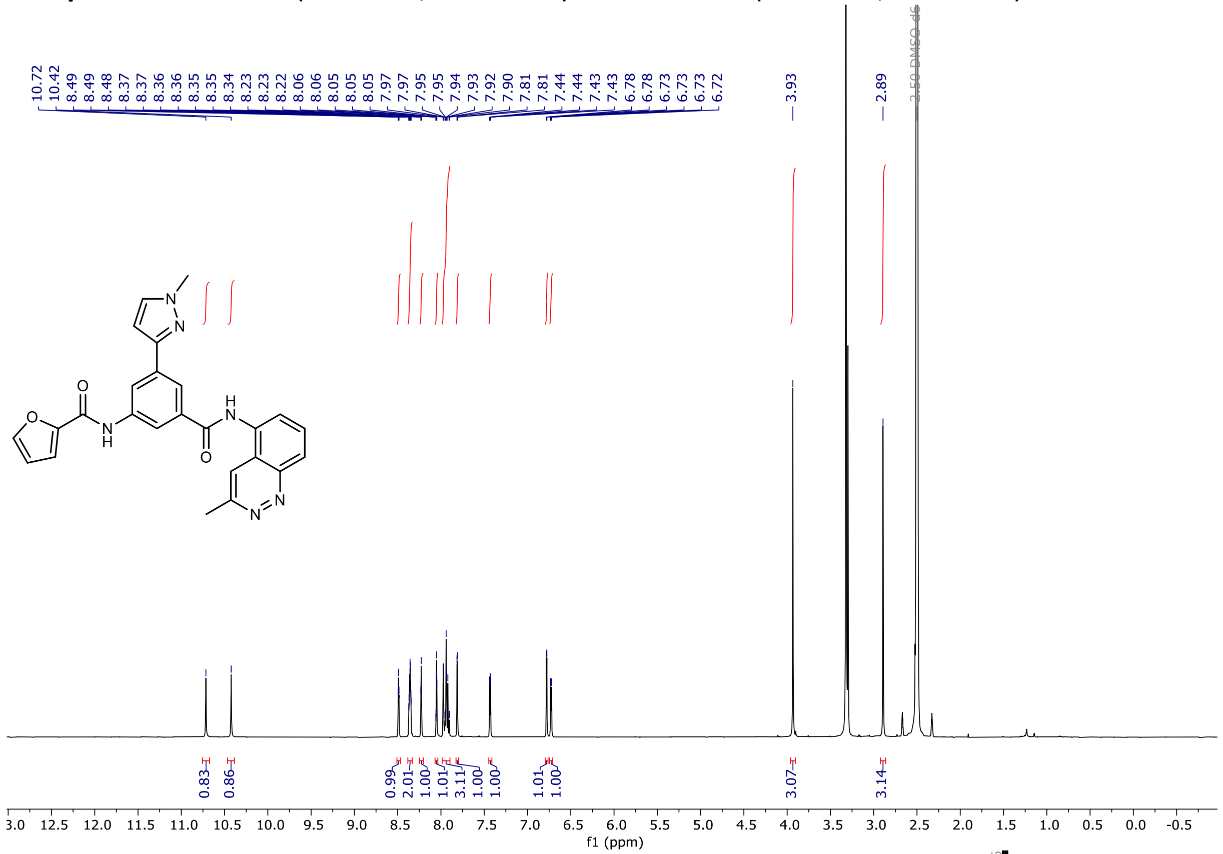


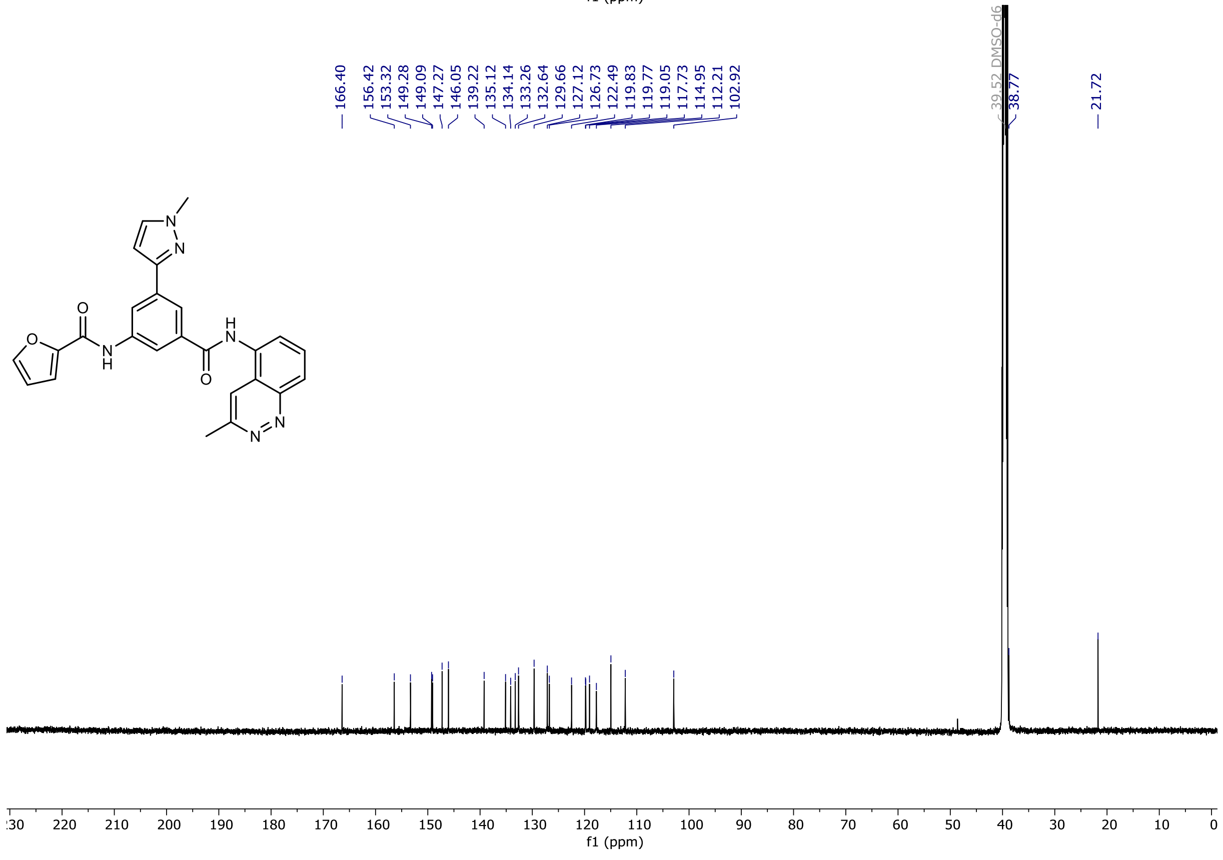

**Compound 3** $^1$H NMR (400 MHz, DMSO-*d6*) and $^{13}$C NMR (126 MHz, DMSO-*d6*)

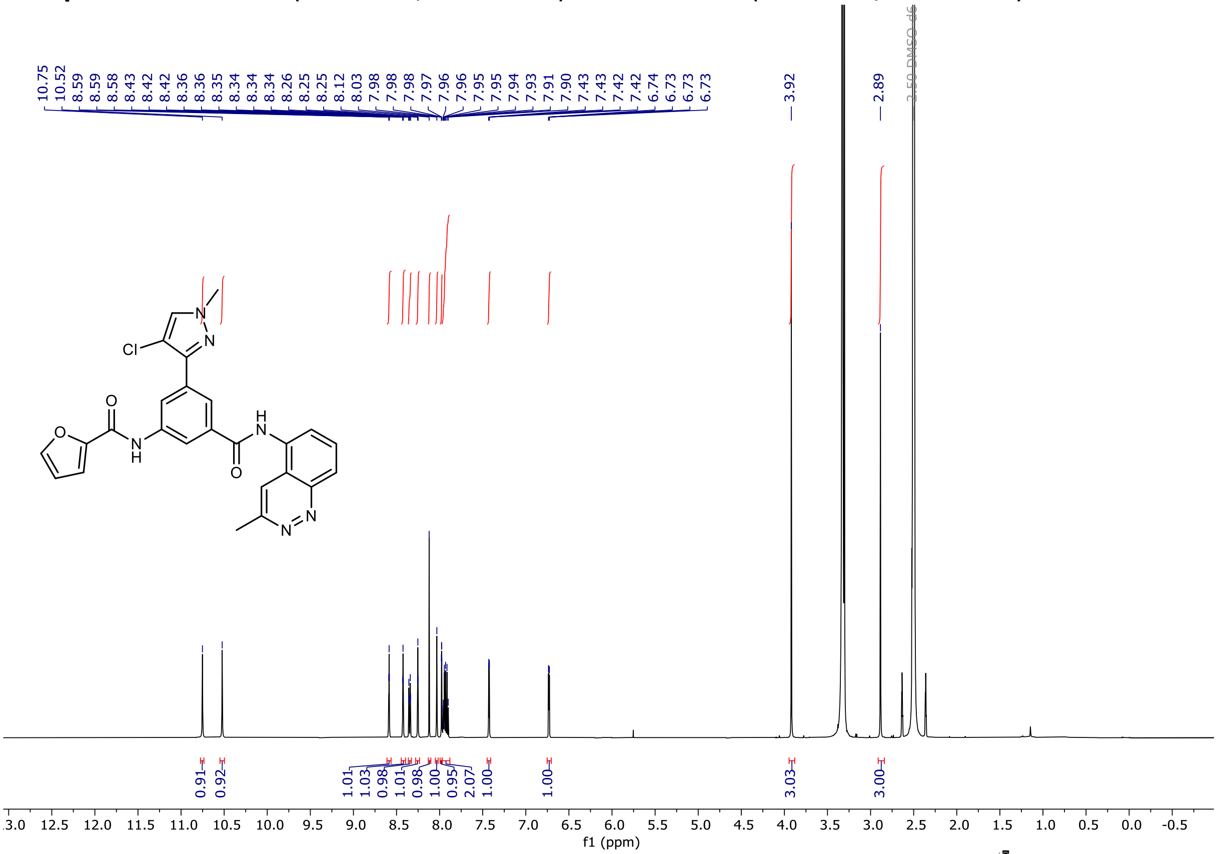


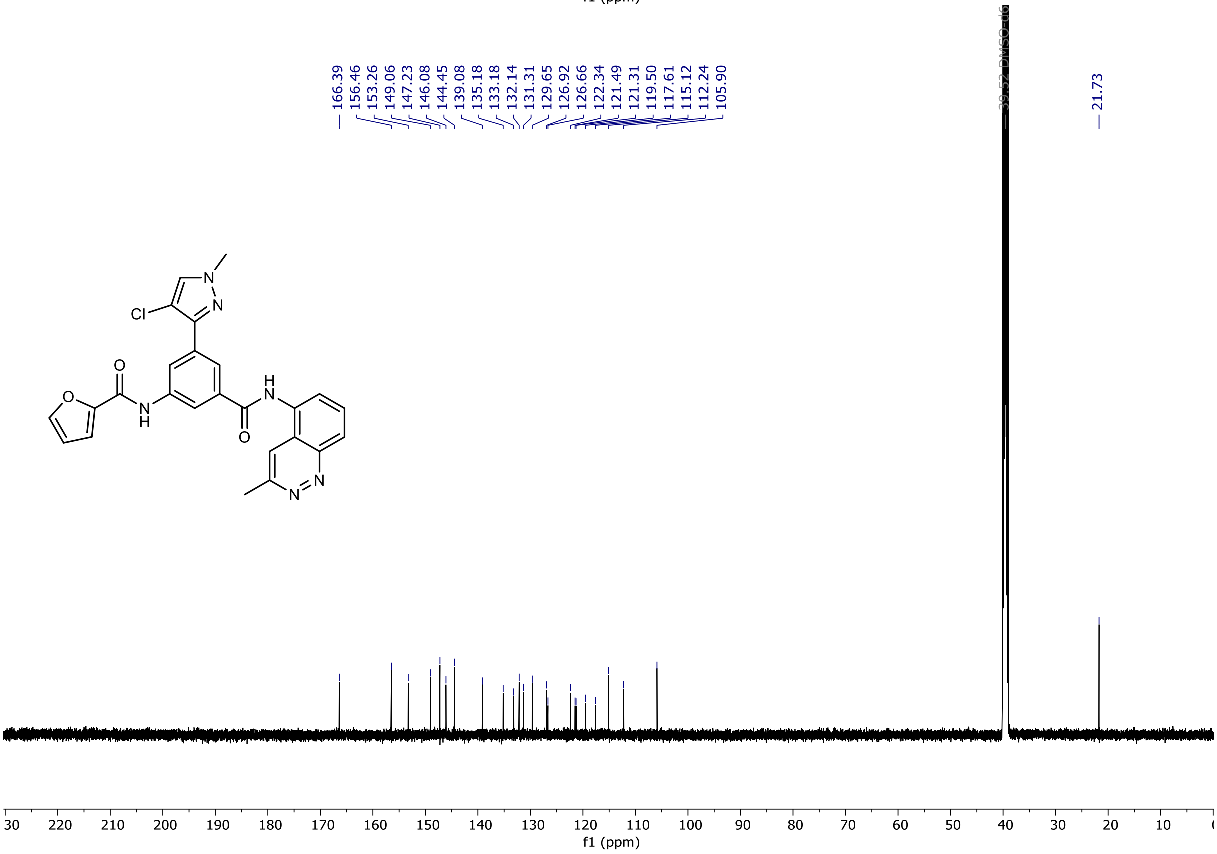

**Compound 4** $^{1}$H NMR (400 MHz, DMSO-*d6*) and $^{13}$C NMR (126 MHz, DMSO-*d*$_6$)

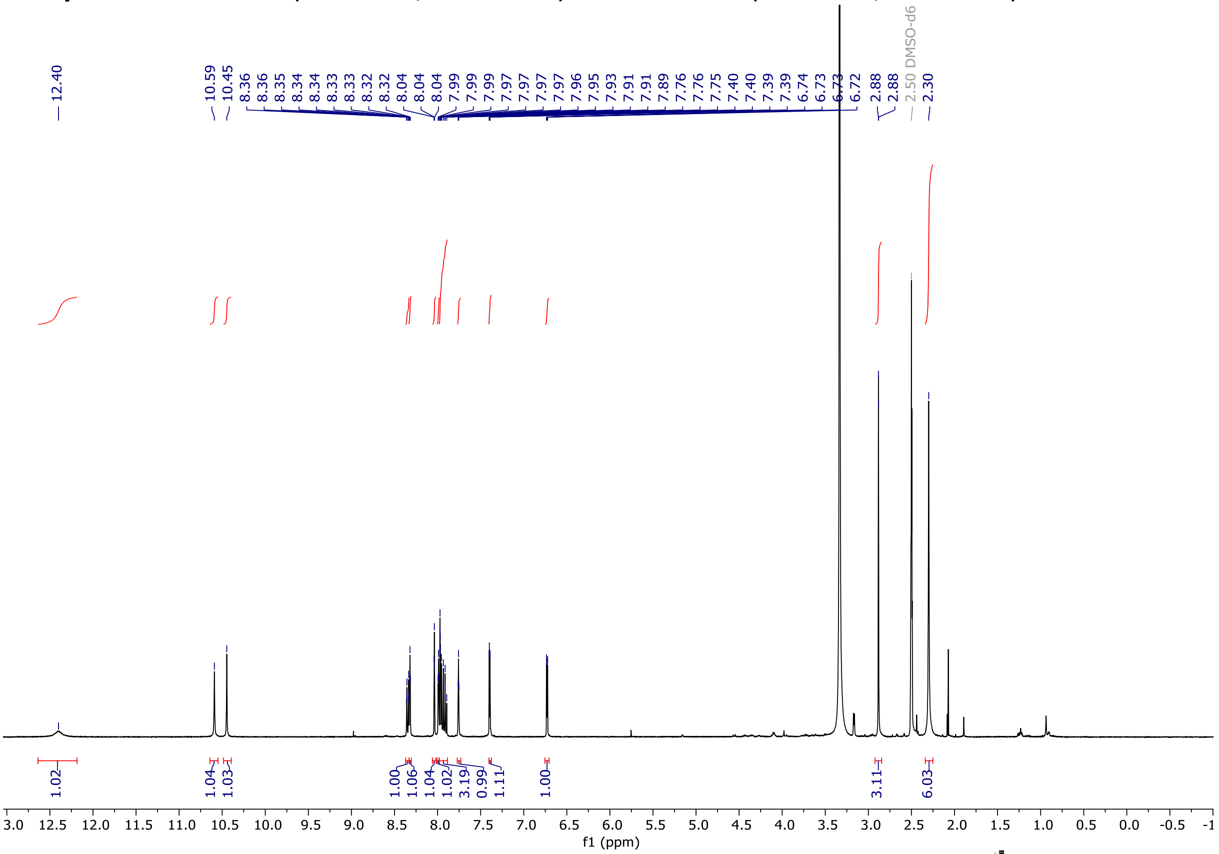

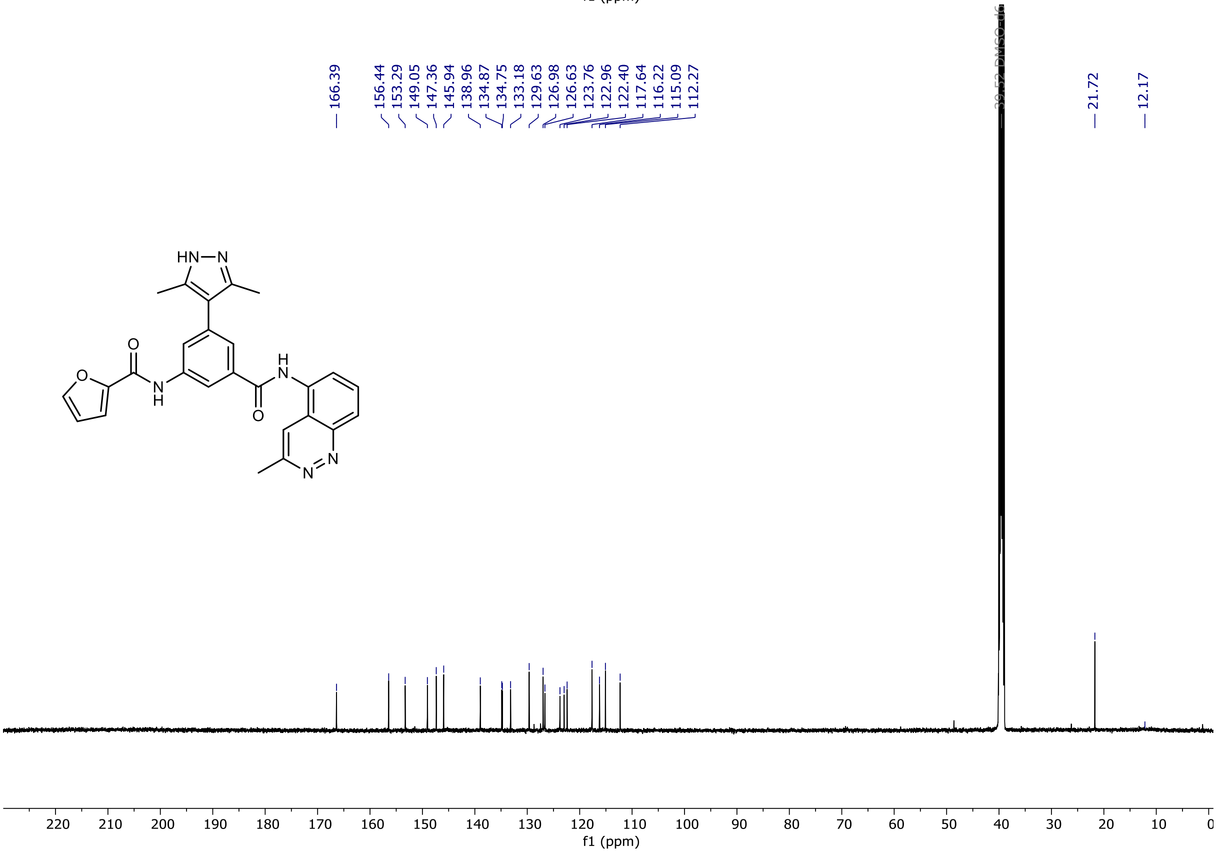

**Compound 5** $^{1}H$ NMR (500 MHz, DMSO-$d_6$), $^{13}C$ NMR (126 MHz, DMSO-$d_6$) and HPLC

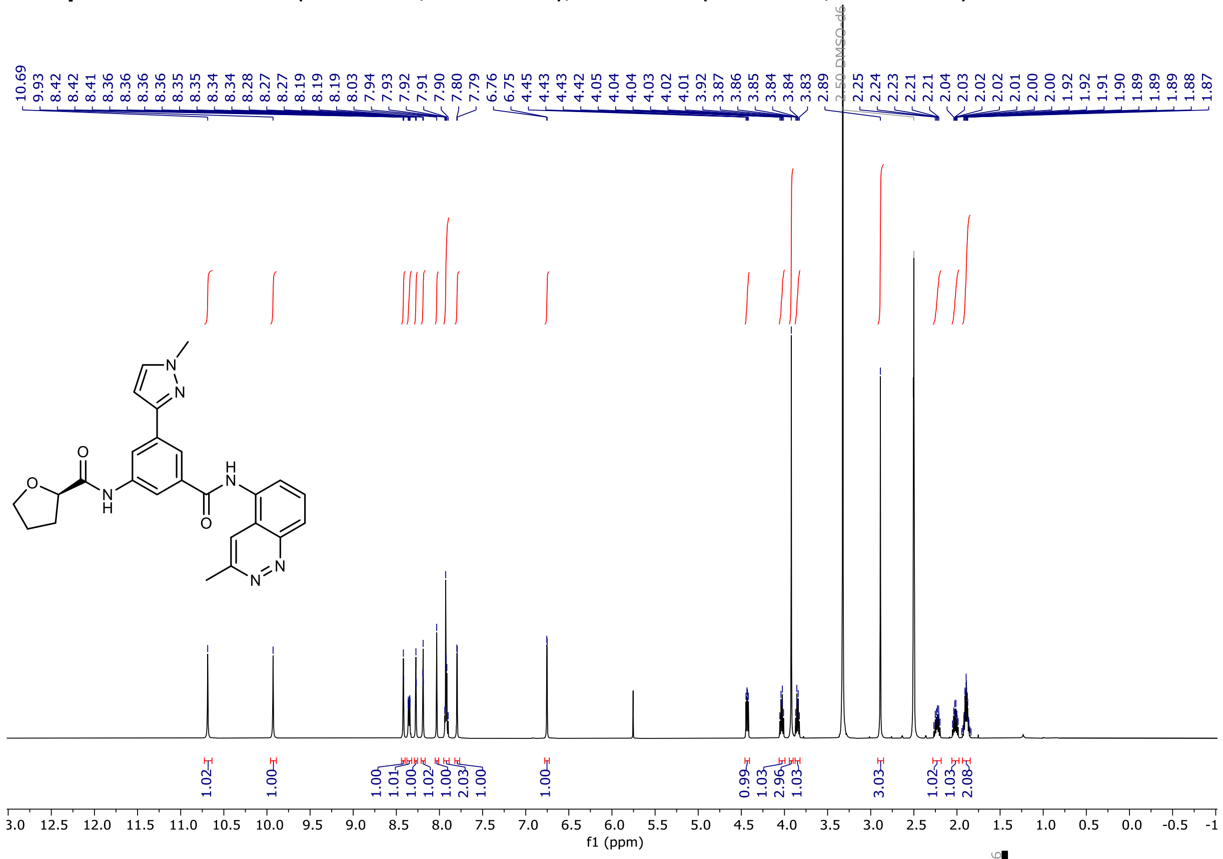

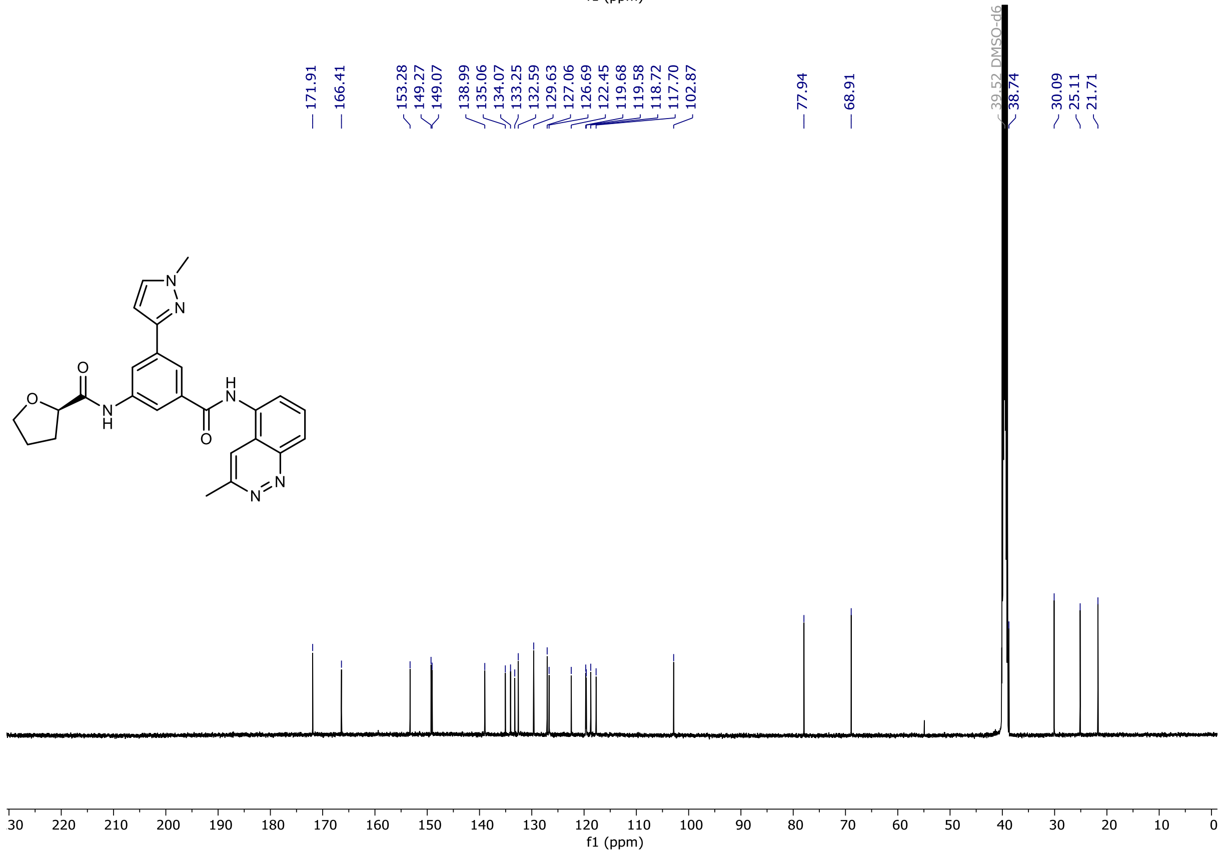

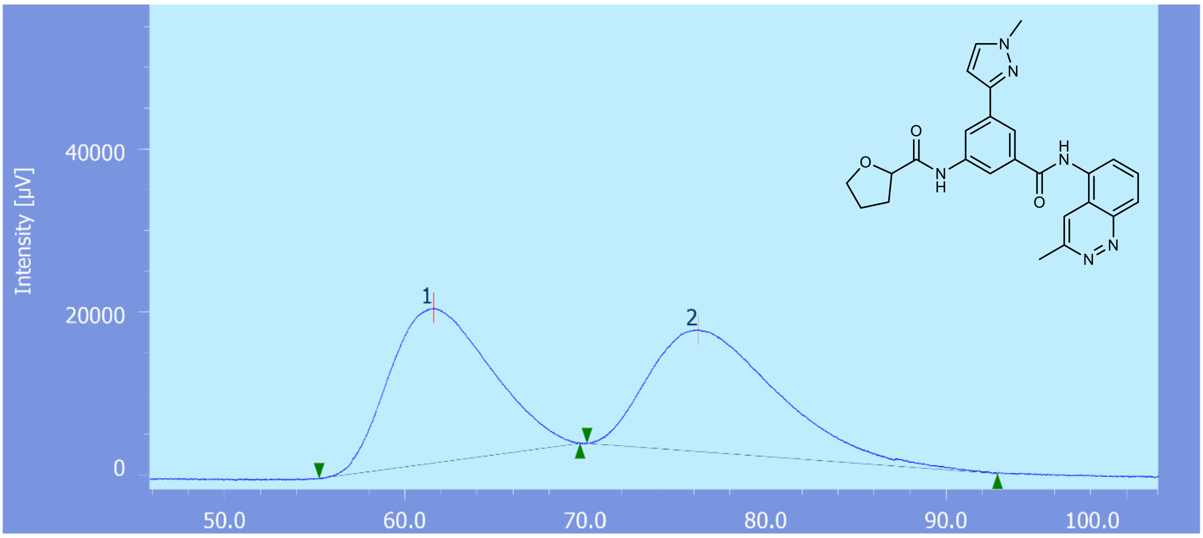


| # | Peak Name | CH | tR [min] | Area [µV·sec] | Height [µV] | Area% | Height% | Quantity | NTP | Resolution | Symmetry Factor | Warning |
|---|---|---|---|---|---|---|---|---|---|---|---|---|
| 1 | Unknown | 5 | 61.597 | 7428935 | 19011 | 49.896 | 56.019 | N/A | 523 | 1.223 | 1.255 | |
| 2 | Unknown | 5 | 76.253 | 7459765 | 14926 | 50.104 | 43.981 | N/A | 528 | N/A | 1.583 | |

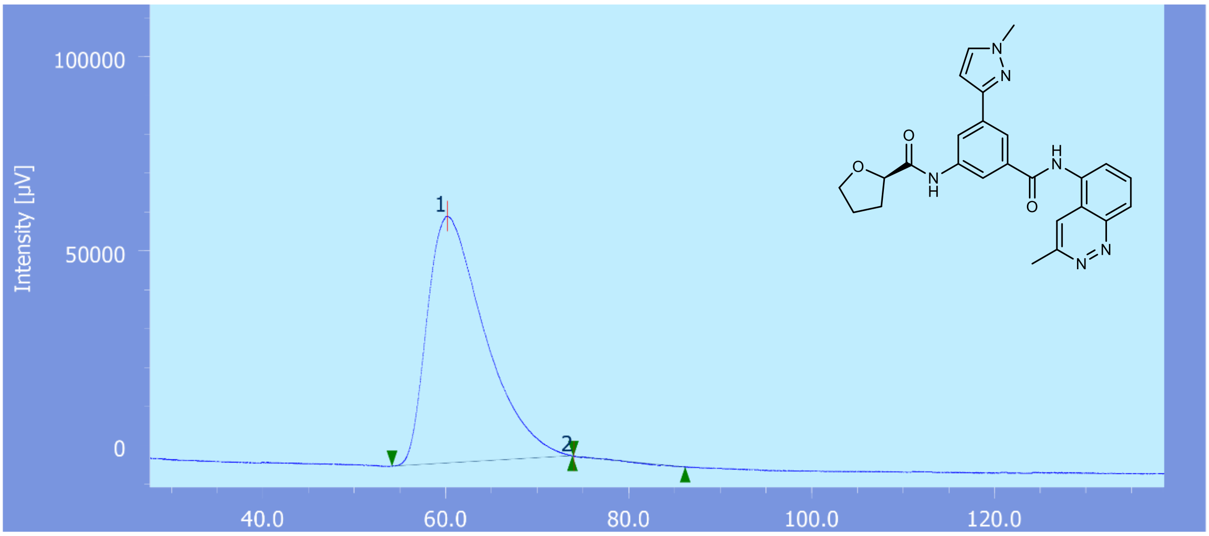


| # | Peak Name | CH | tR [min] | Area [µV·sec] | Height [µV] | Area% | Height% | Quantity | NTP | Resolution | Symmetry Factor | Warning |
|---|---|---|---|---|---|---|---|---|---|---|---|---|
| 1 | Unknown | 5 | 60.173 | 27848432 | 63429 | 99.962 | 99.865 | N/A | 444 | 0.867 | 1.750 | |
| 2 | Unknown | 5 | 74.010 | 10574 | 86 | 0.038 | 0.135 | N/A | 207 | N/A | 211.404 | |

**Compound 6** $^{1}$H NMR (500 MHz, DMSO-$d_6$), $^{13}$C NMR (126 MHz, DMSO-$d_6$) and HPLC

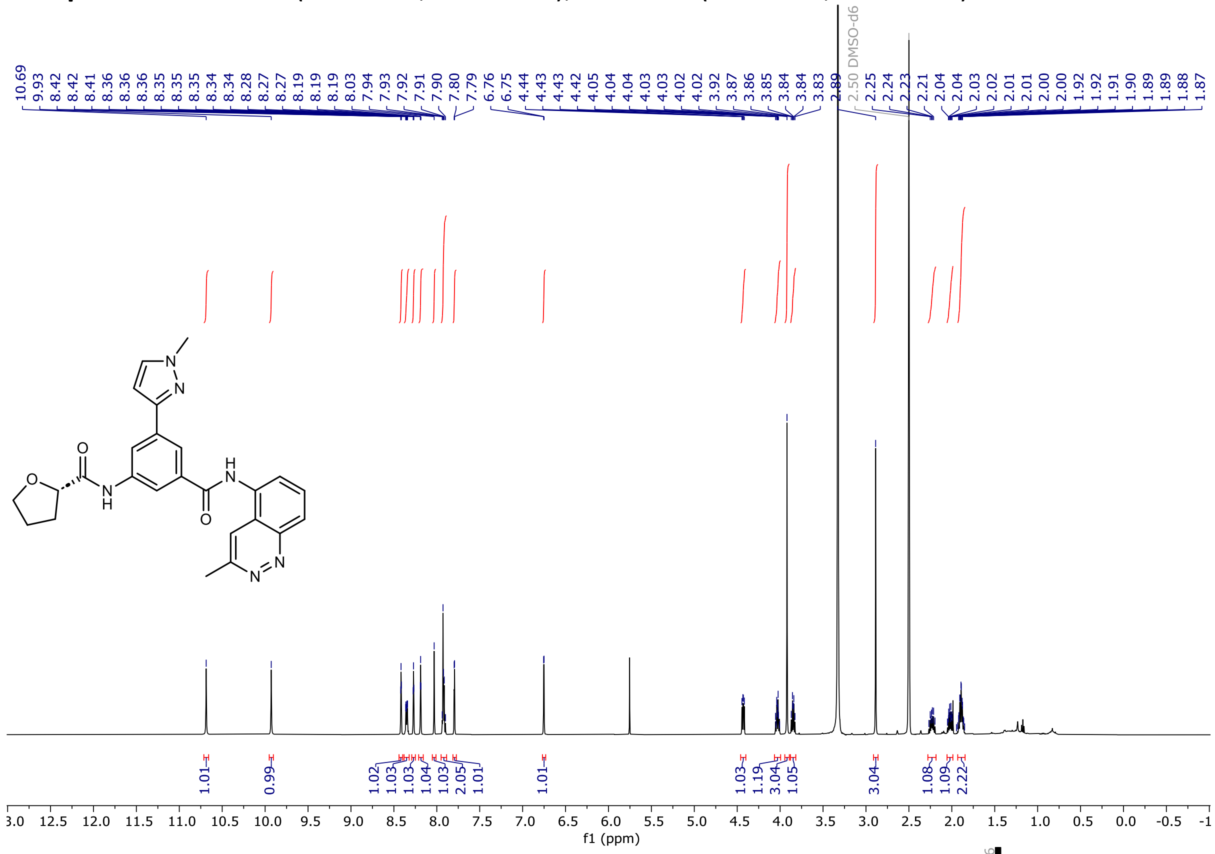


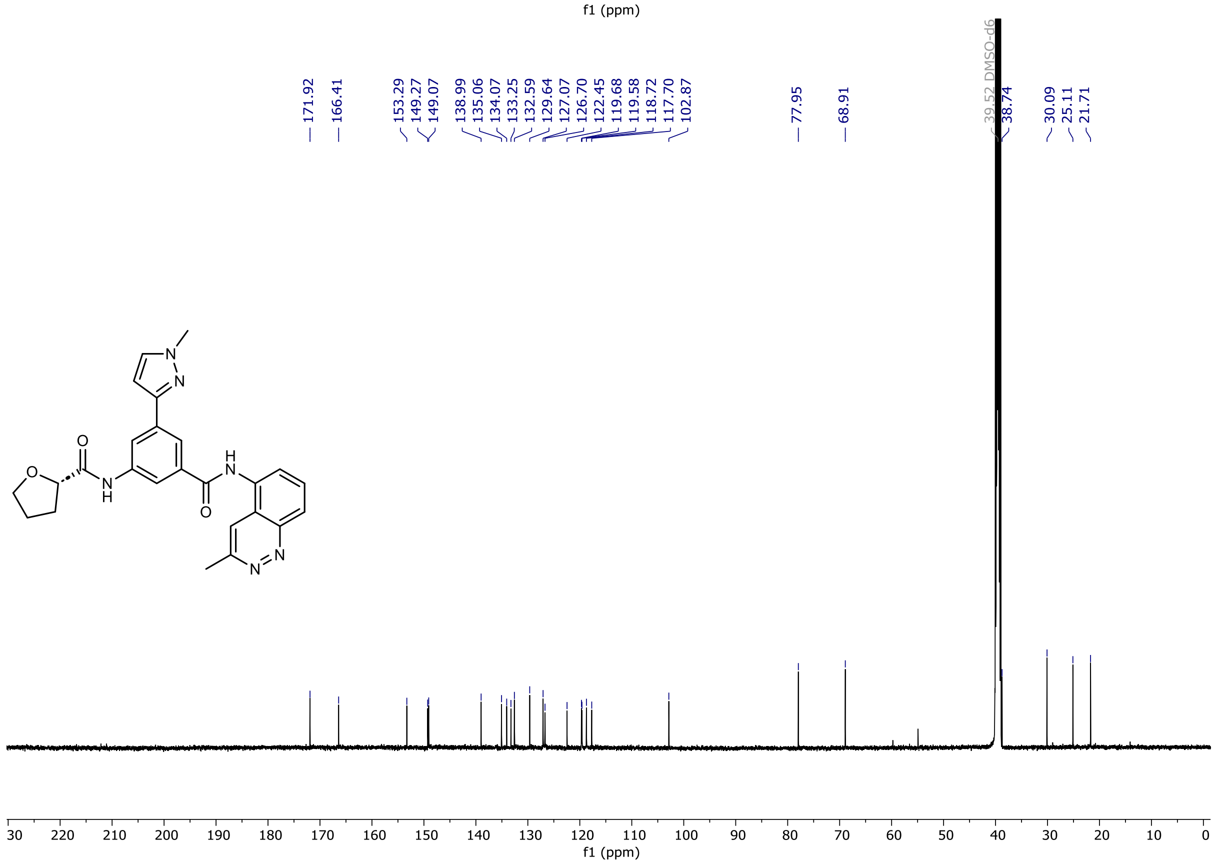

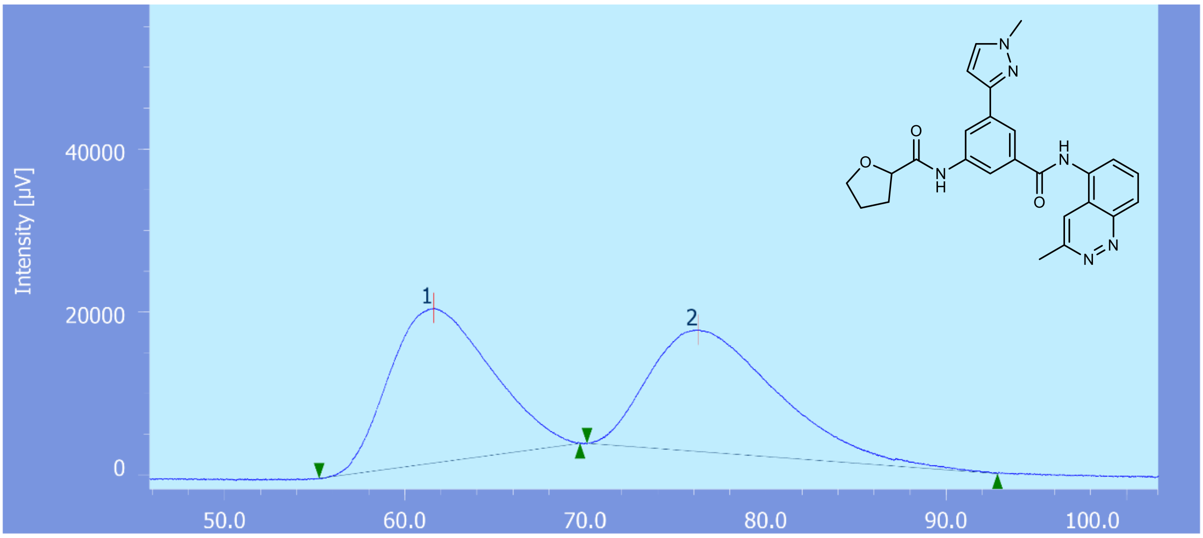


| # | Peak Name | CH | tR [min] | Area [μV·sec] | Height [μV] | Area% | Height% | Quantity | NTP | Resolution | Symmetry Factor | Warning |
|---|---|---|---|---|---|---|---|---|---|---|---|---|
| 1 | Unknown | 5 | 61.597 | 7428935 | 19011 | 49.896 | 56.019 | N/A | 523 | 1.223 | 1.255 | |
| 2 | Unknown | 5 | 76.253 | 7459765 | 14926 | 50.104 | 43.981 | N/A | 528 | N/A | 1.583 | |

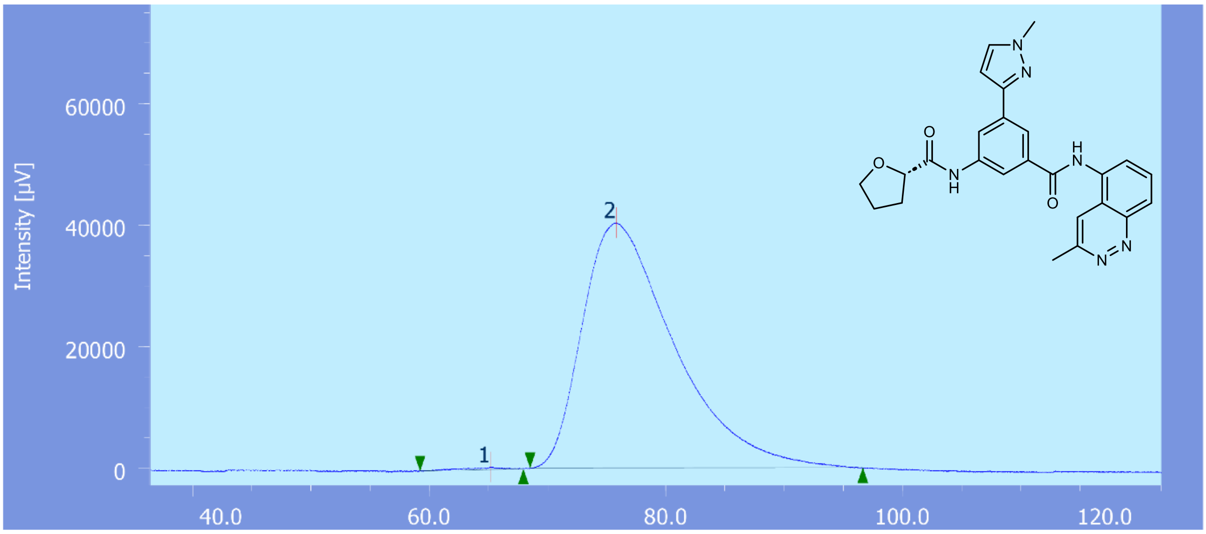


| # | Peak Name | CH | tR [min] | Area [μV·sec] | Height [μV] | Area% | Height% | Quantity | NTP | Resolution | Symmetry Factor | Warning |
|---|---|---|---|---|---|---|---|---|---|---|---|---|
| 1 | Unknown | 5 | 65.200 | 83309 | 444 | 0.381 | 1.090 | N/A | 1357 | 1.020 | 0.729 | |
| 2 | Unknown | 5 | 75.823 | 21766156 | 40310 | 99.619 | 98.910 | N/A | 483 | N/A | 1.705 | |

**Compound 7** $^{1}$H NMR (500 MHz, MeOD-*d4*) and $^{13}$C NMR (126 MHz, MeOD-*d4*)

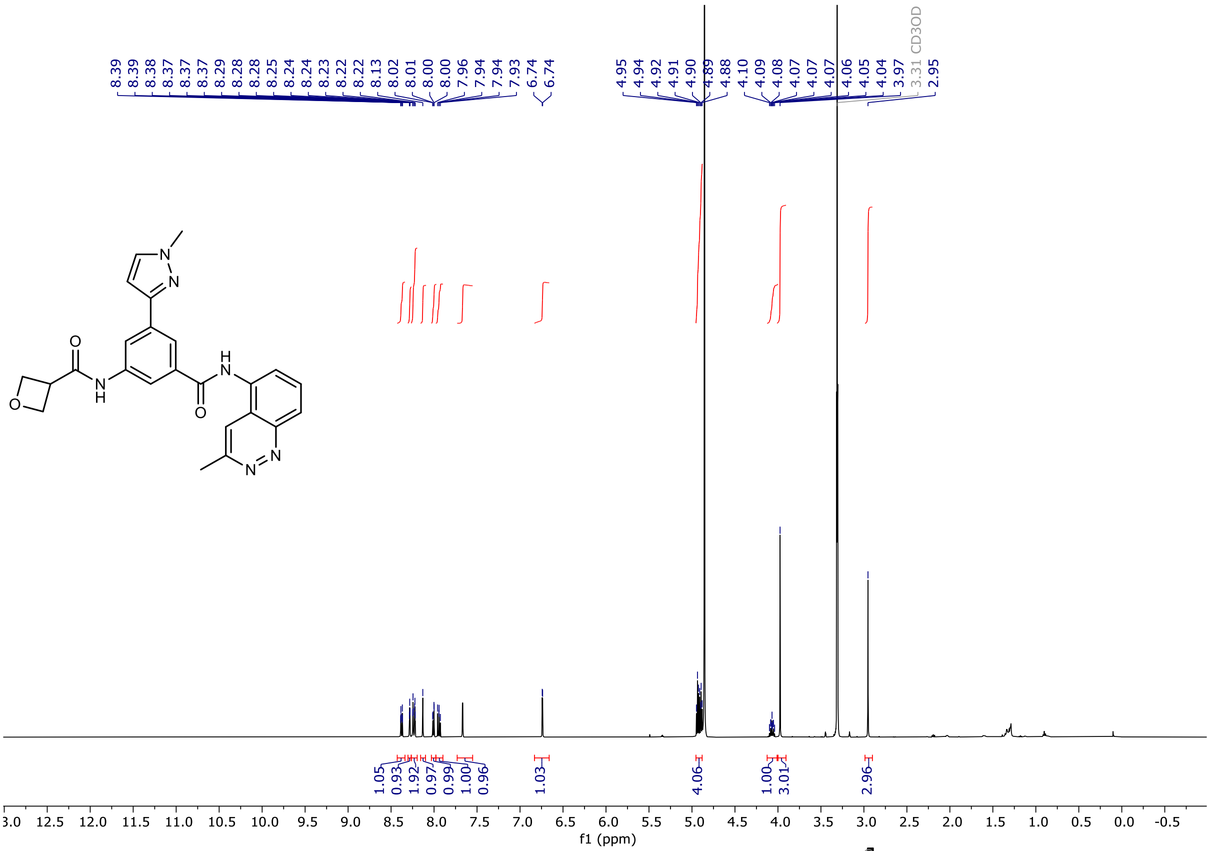


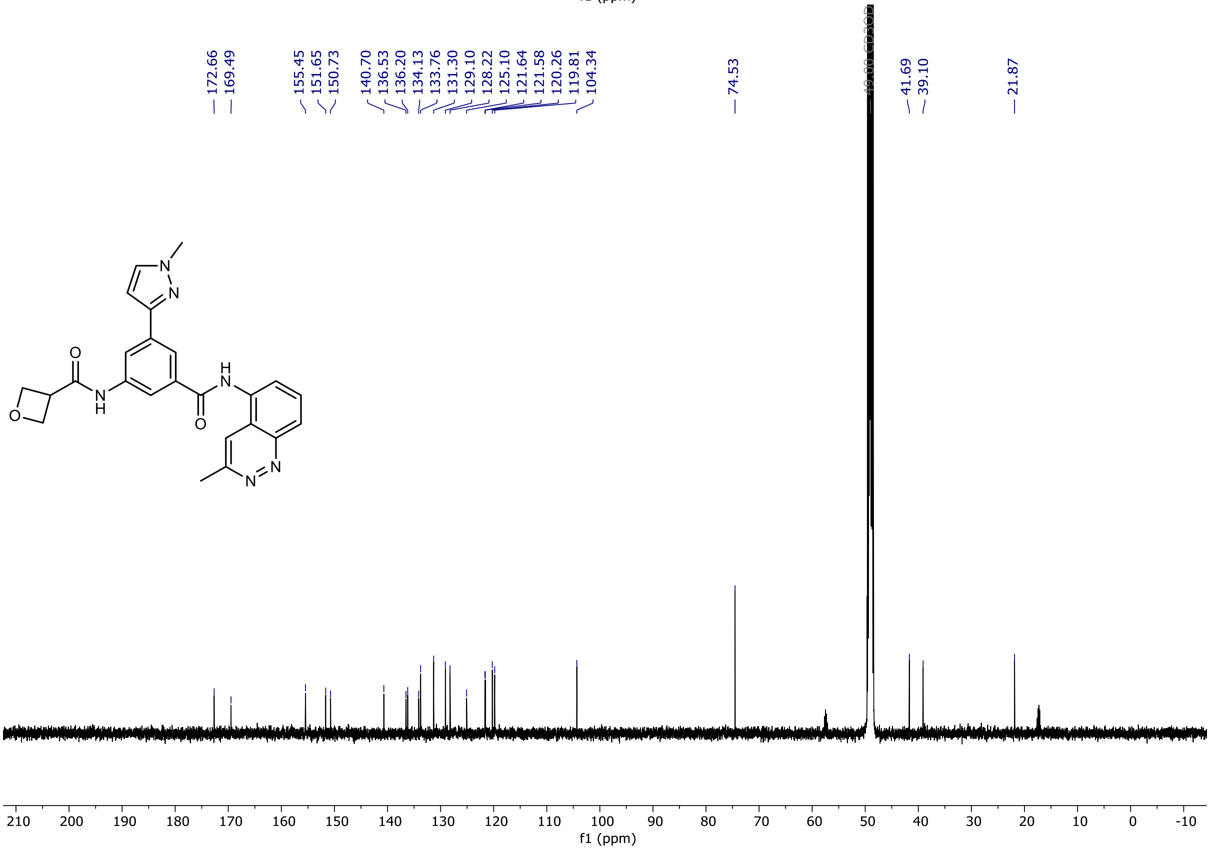

**Compound 8** $^1H$ NMR (500 MHz, $CD_2Cl_2$) and $^{13}C$ NMR (126 MHz, $CD_2Cl_2$)

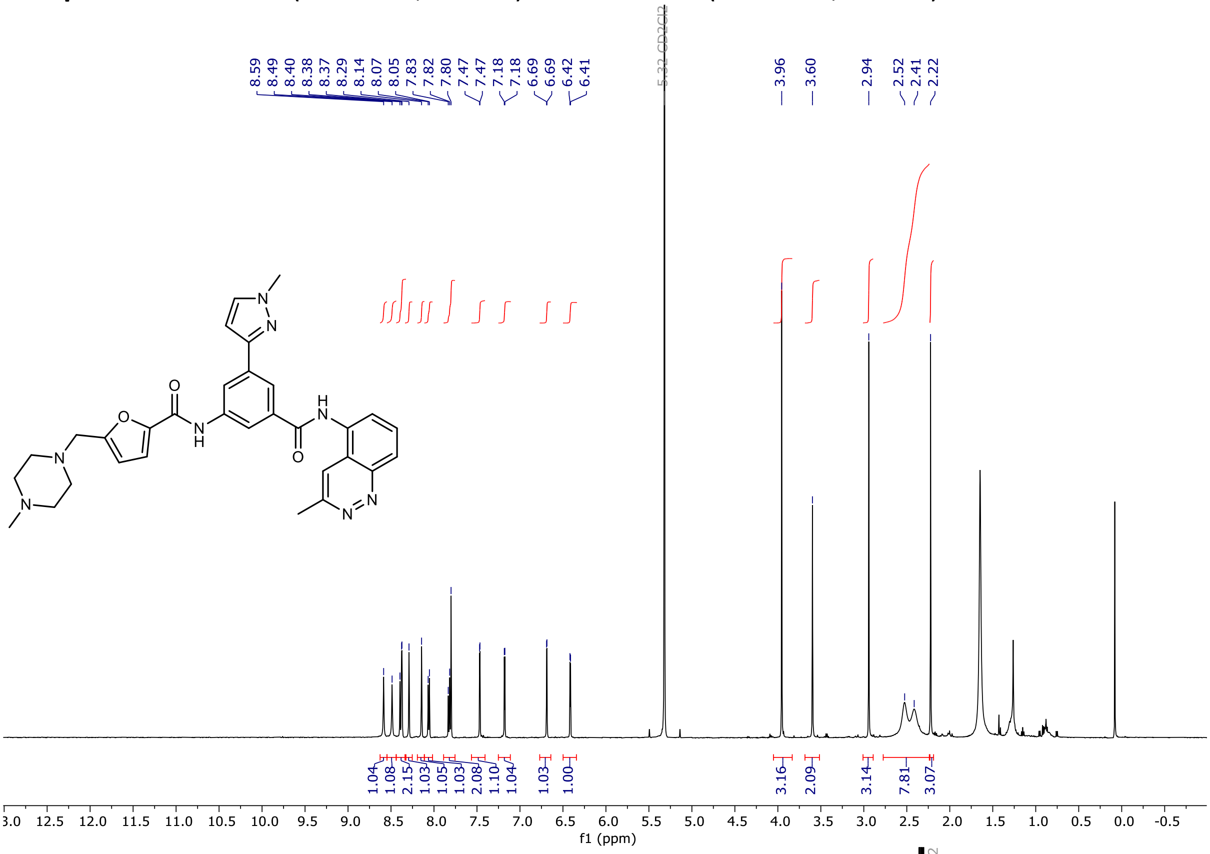

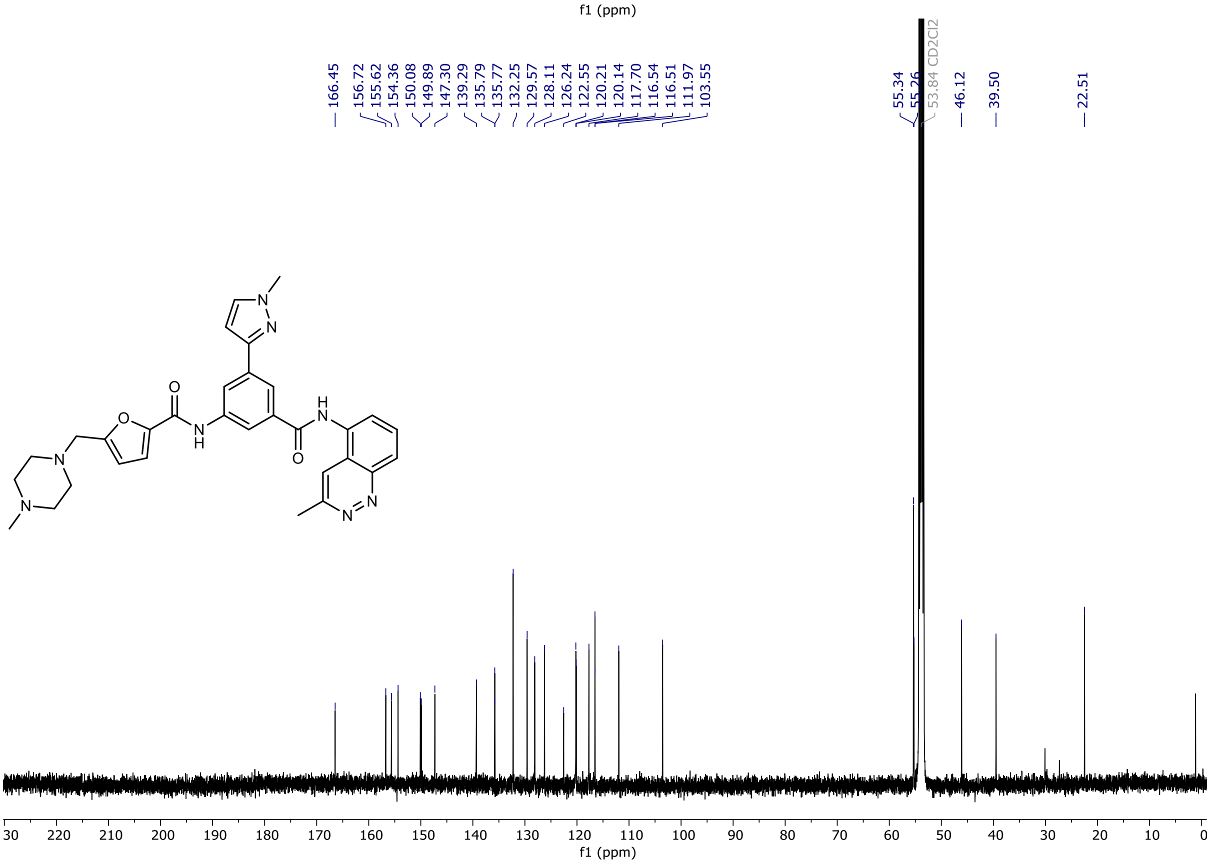

**Compound S9** $^{1}$H NMR (400 MHz, DMSO-*d6*) and $^{13}$C NMR (101 MHz, DMSO-*d6*)

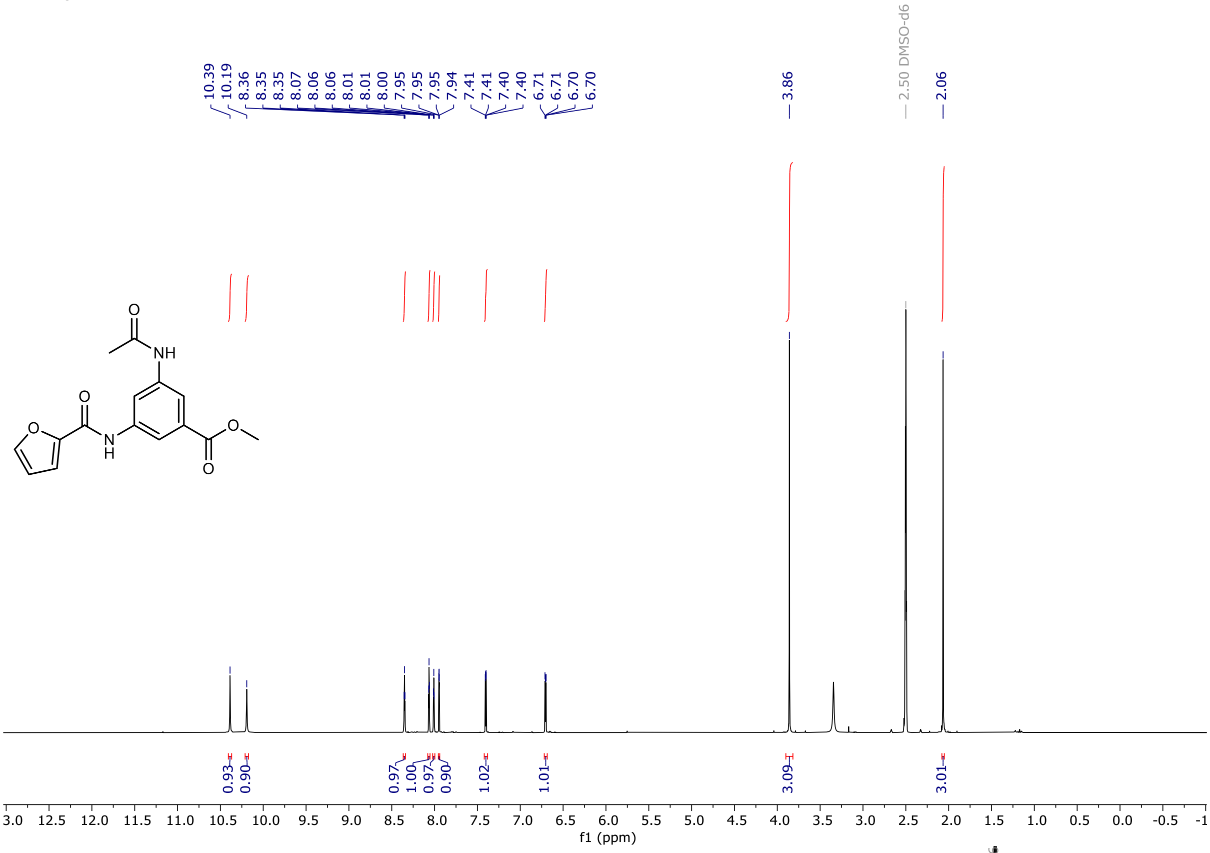


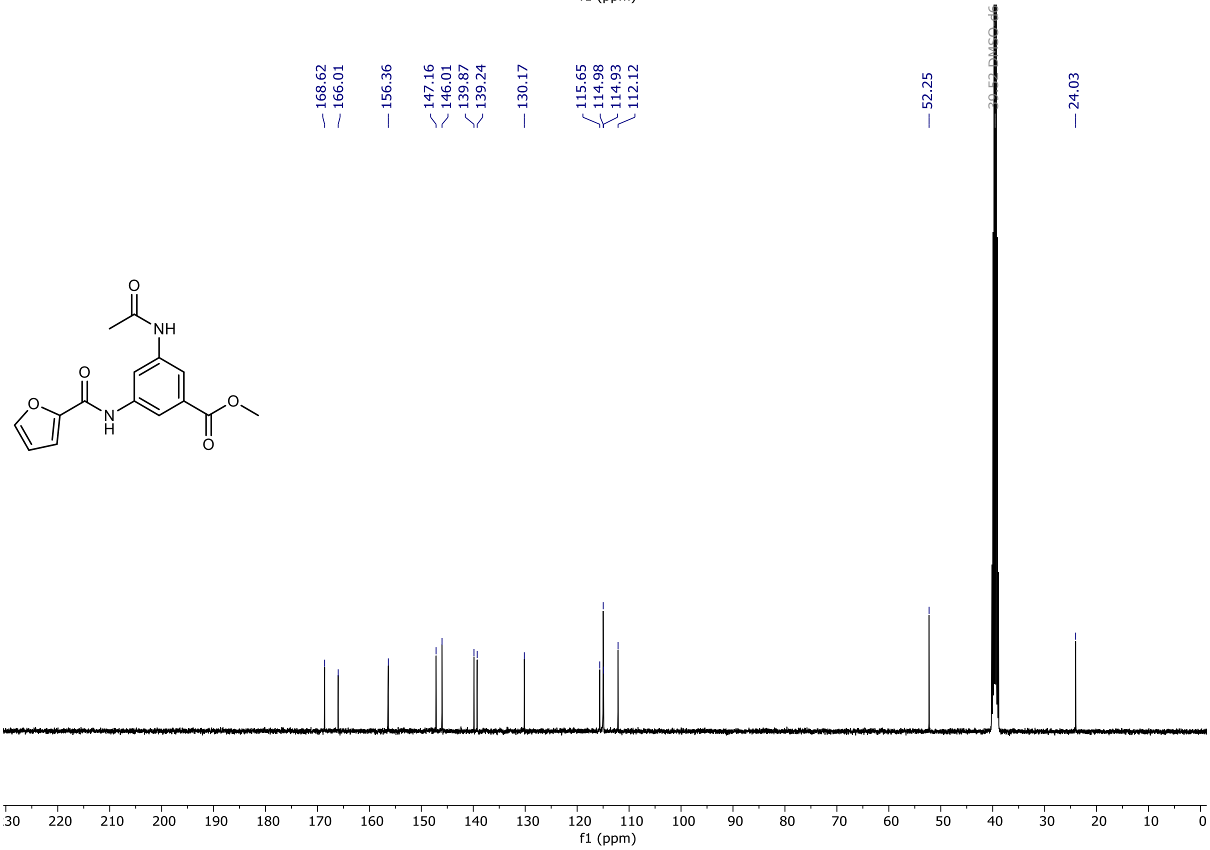

**Compound S10** $^{1}$H NMR (400 MHz, DMSO-*d6*) and $^{13}$C NMR (101 MHz, DMSO-*d6*)

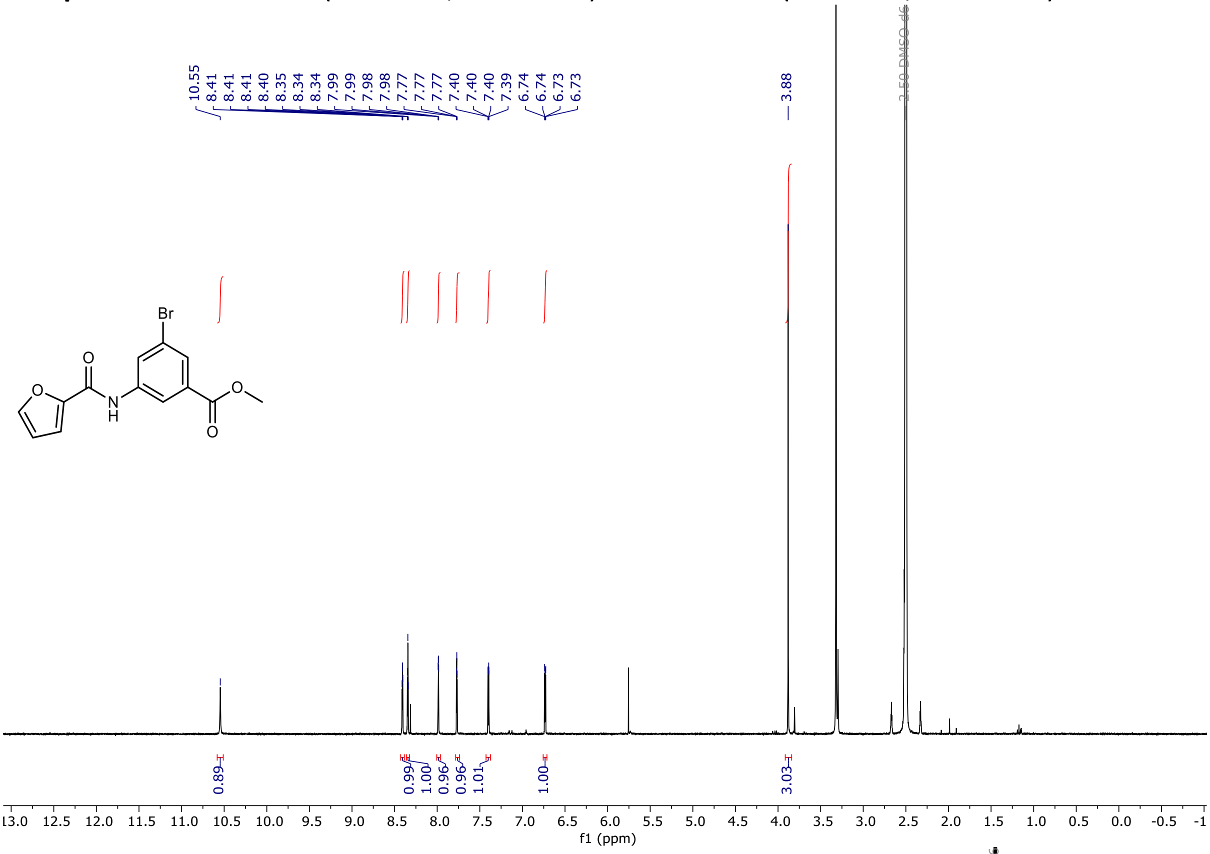


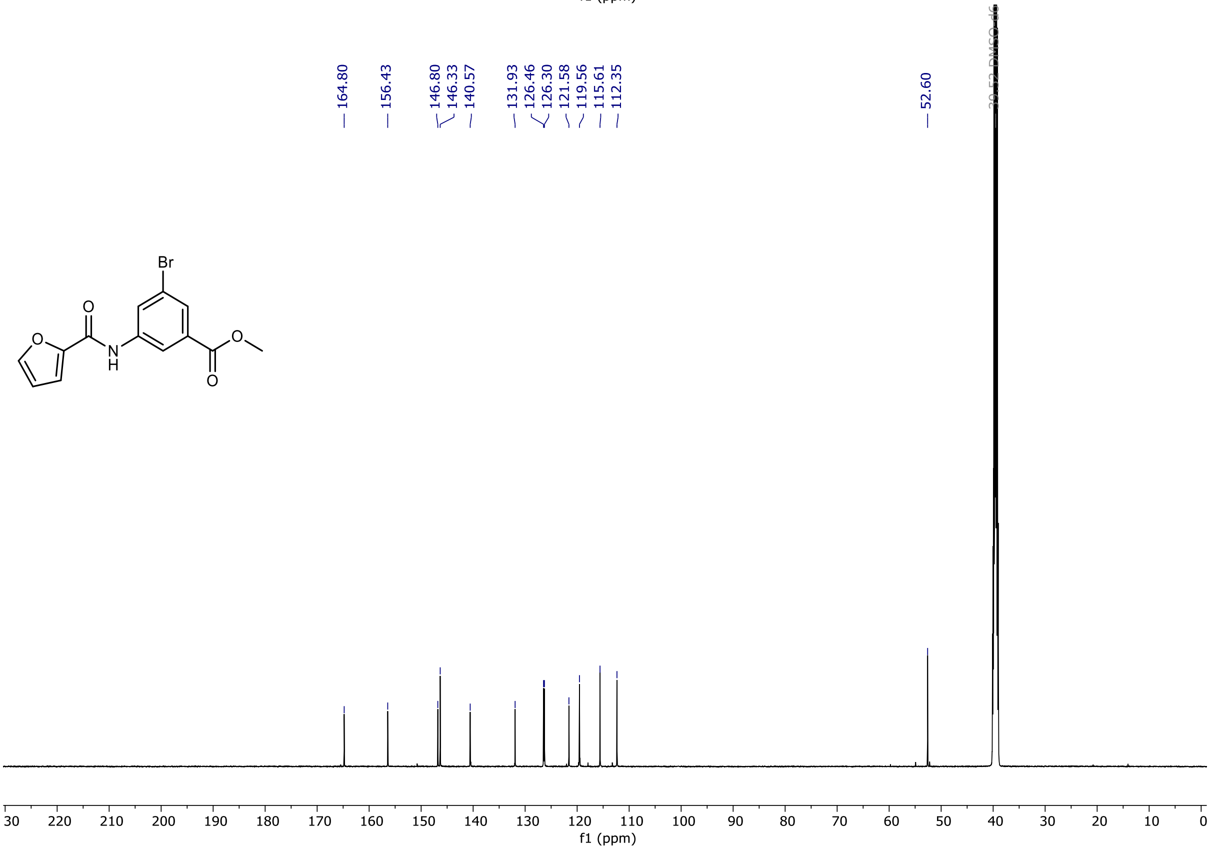

**Compound S11** $^{1}$H NMR (400 MHz, $CDCl_3$) and $^{13}$C NMR (126 MHz, $CDCl_3$)

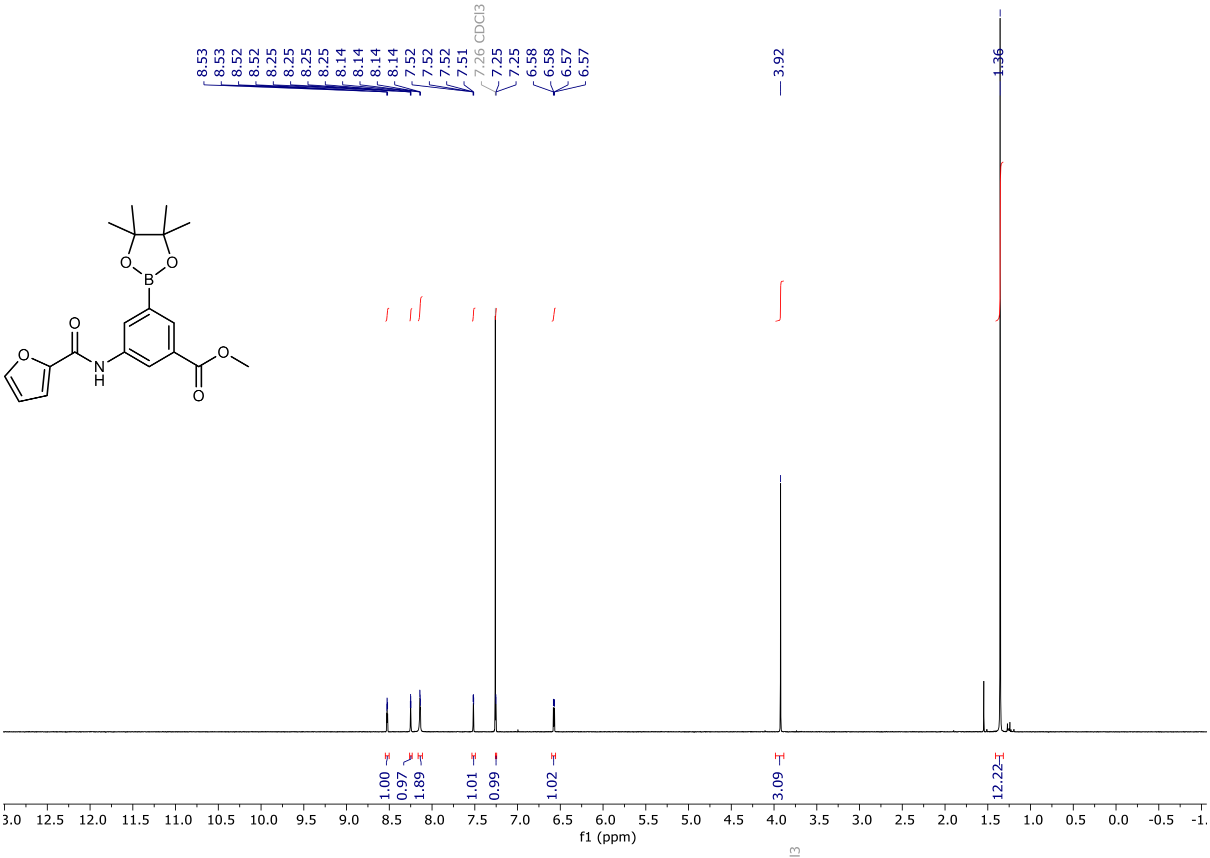


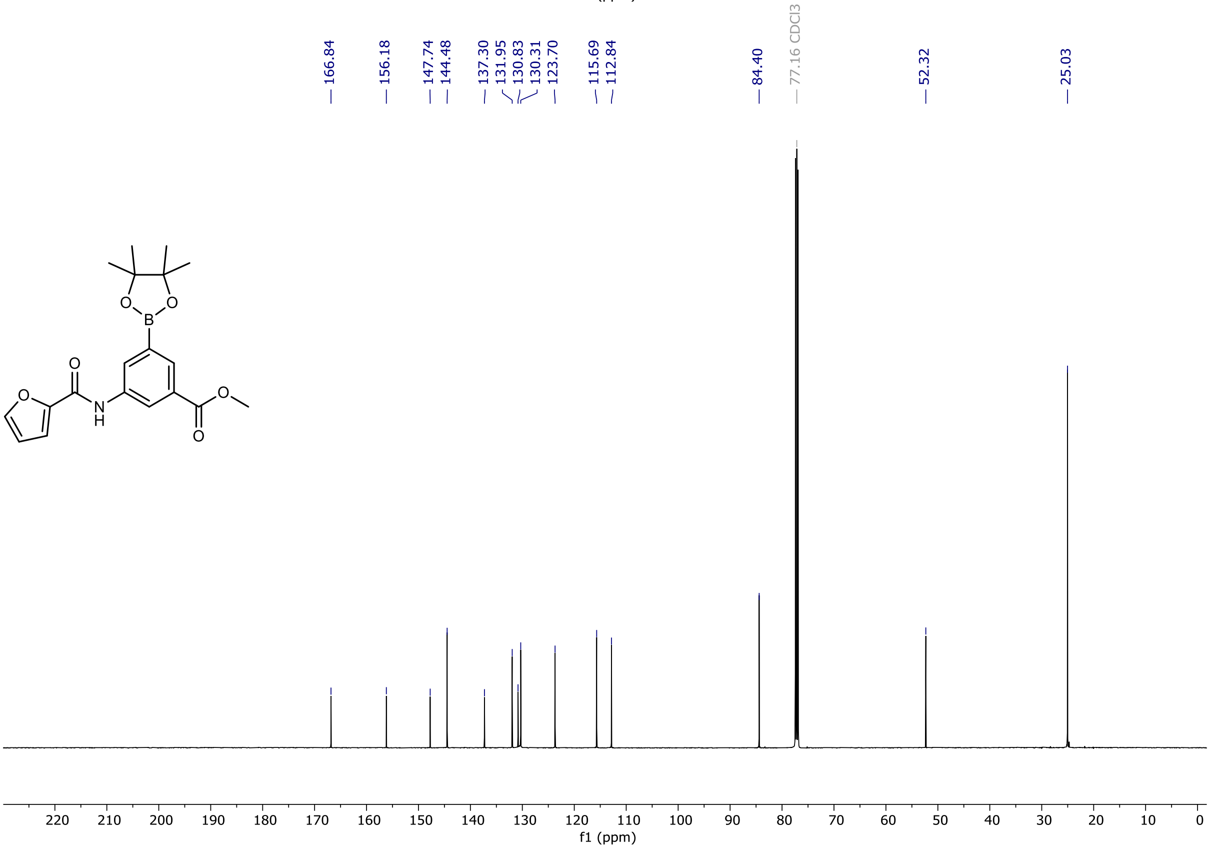

**Compound S12** $^{1}$H NMR (300 MHz, DMSO-$d_6$) and $^{13}$C NMR (101 MHz, DMSO-$d_6$)

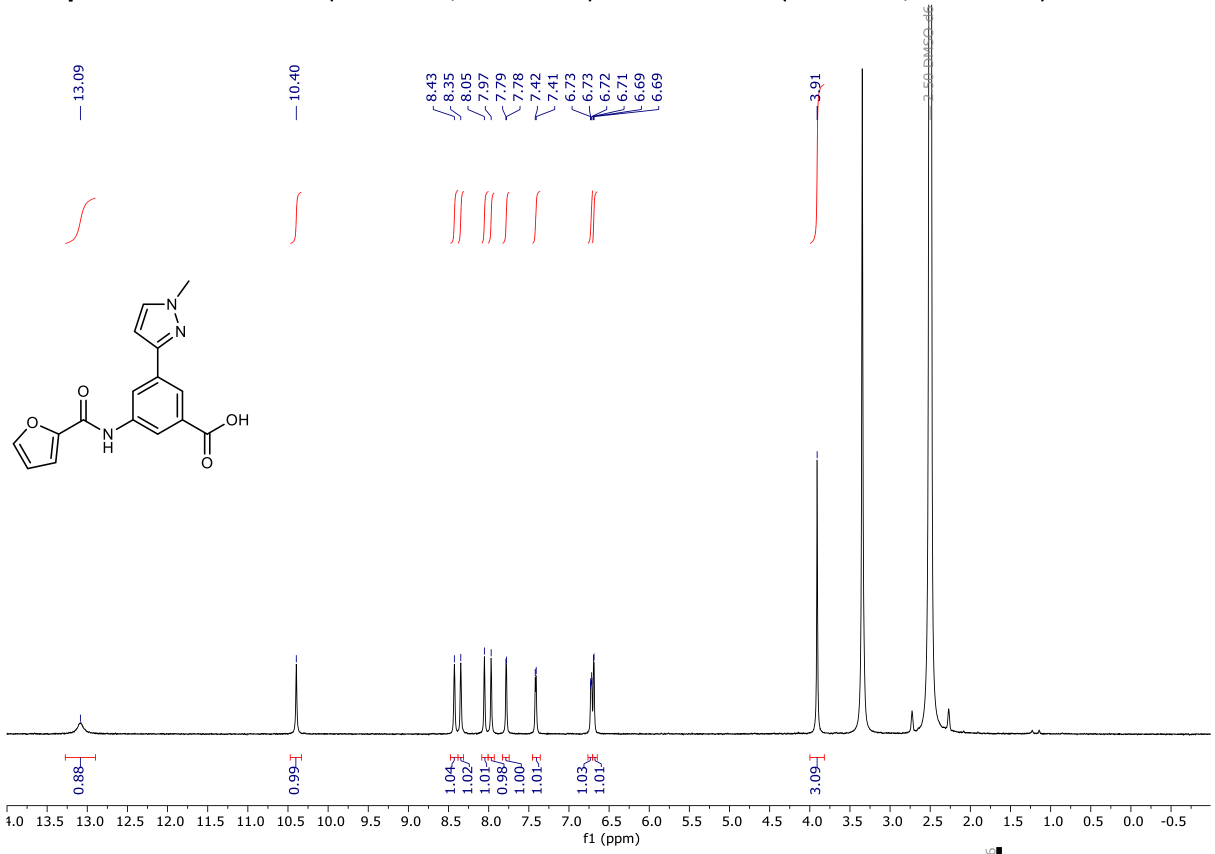


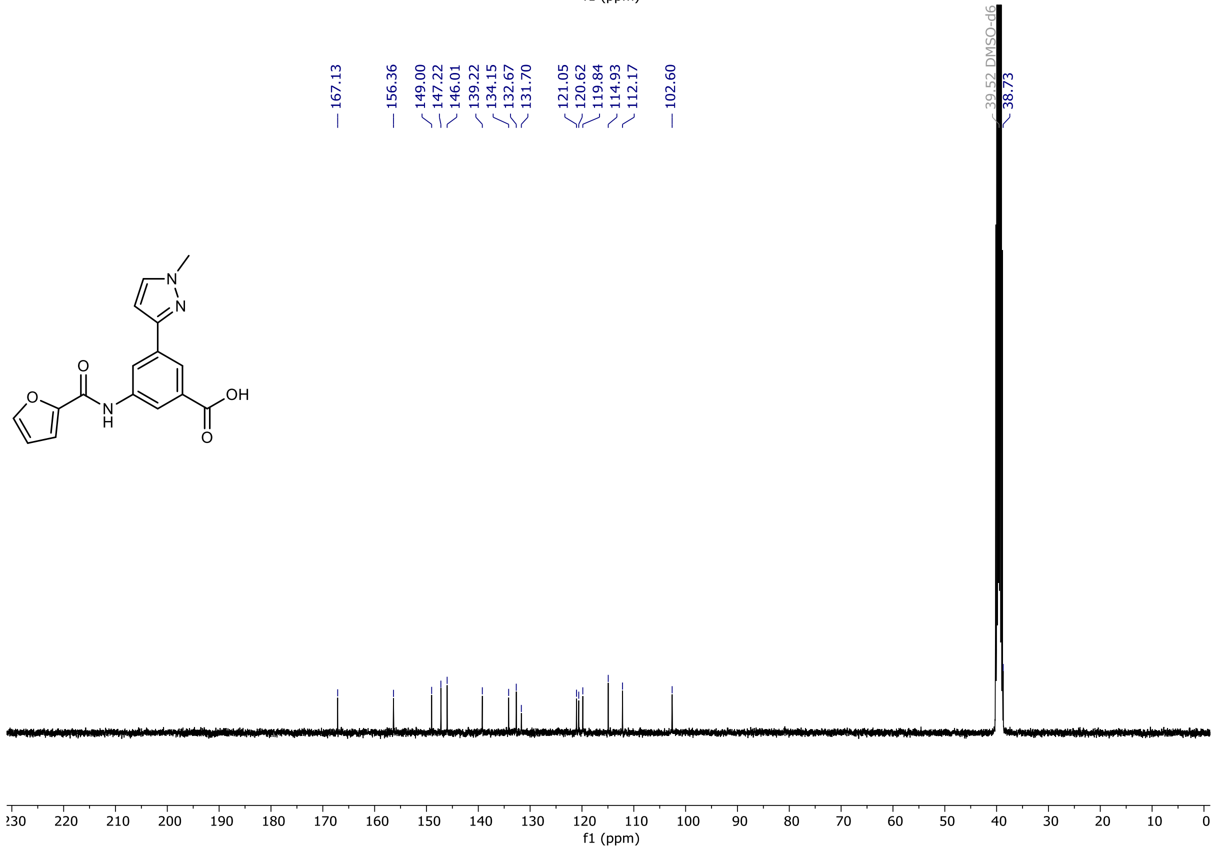

**Compound S13** $^{1}$H NMR (400 MHz, $CDCl_3$) and $^{13}$C NMR (101 MHz, CDCl3)

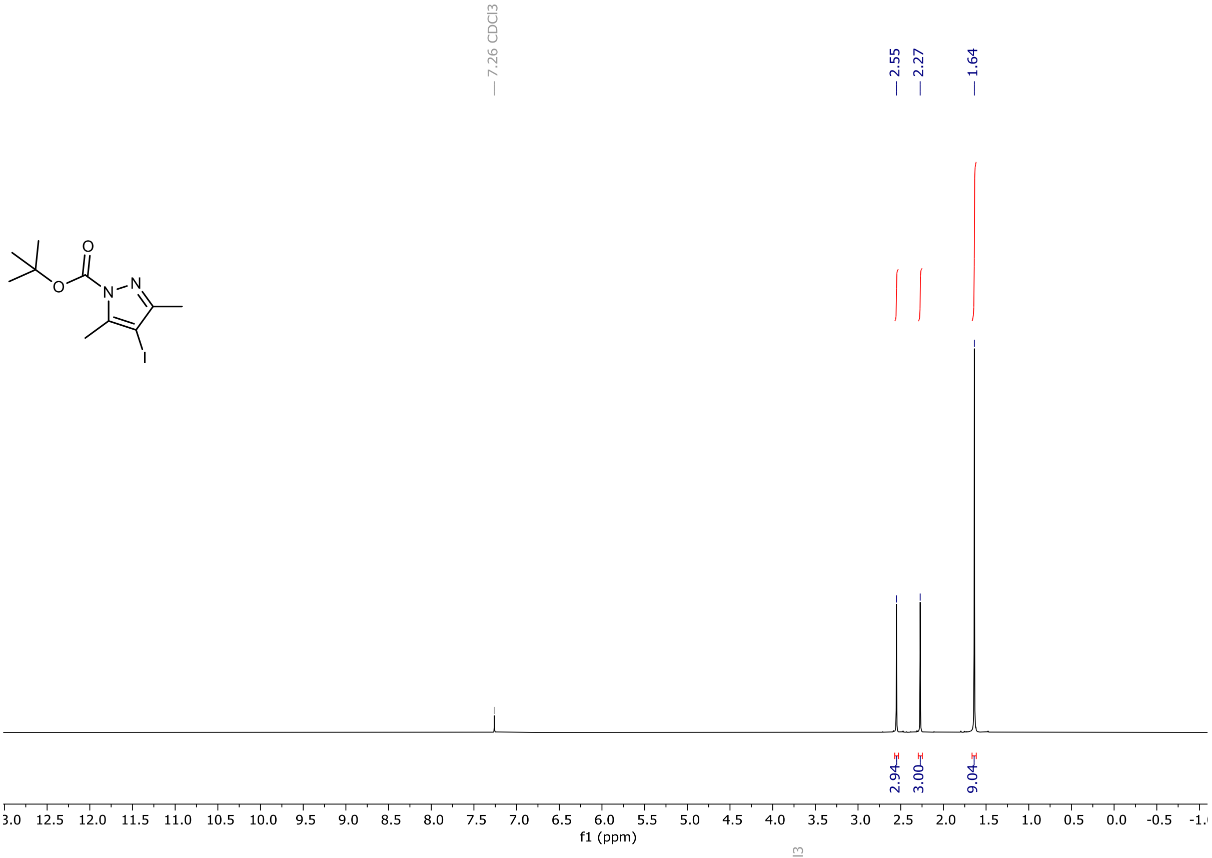


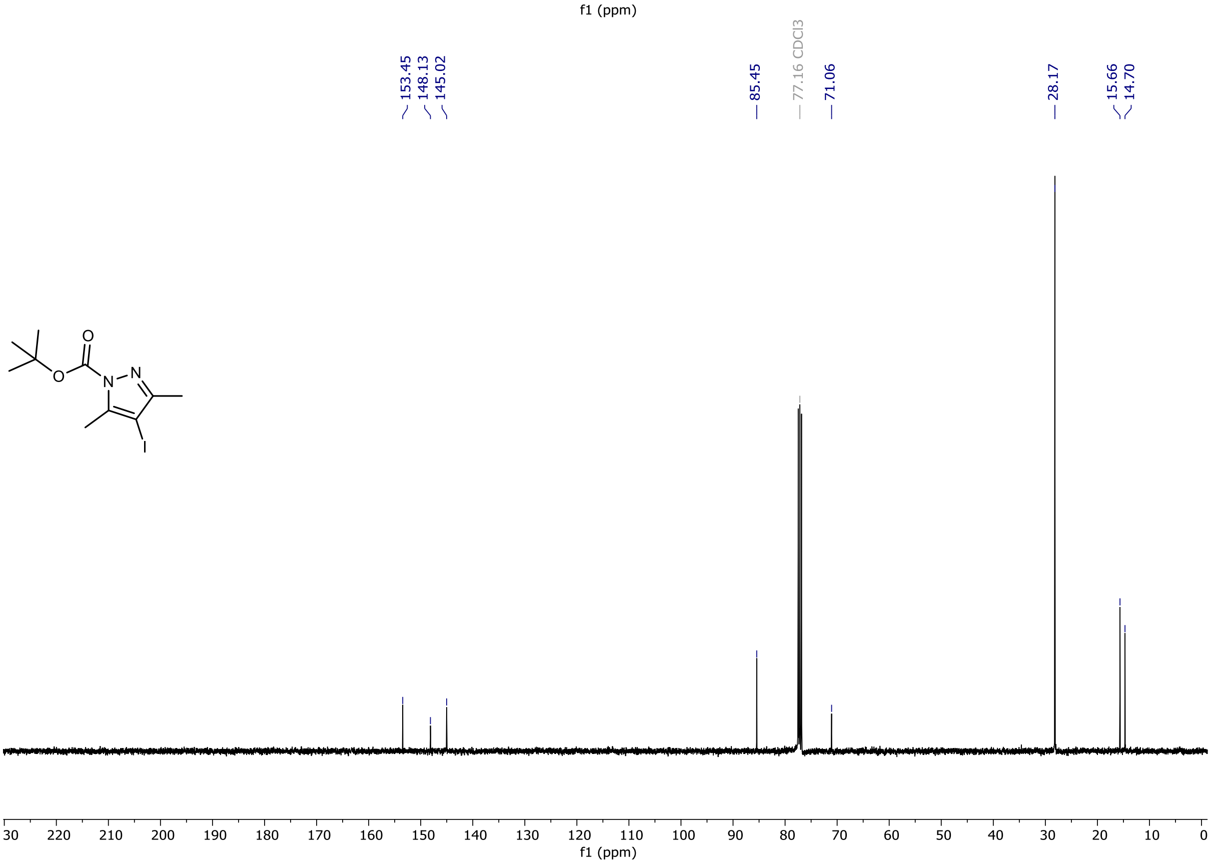

**Compound S14** $^{1}$H NMR (500 MHz, Acetone-$d_6$) and $^{13}$C NMR (126 MHz, Acetone-$d_6$)

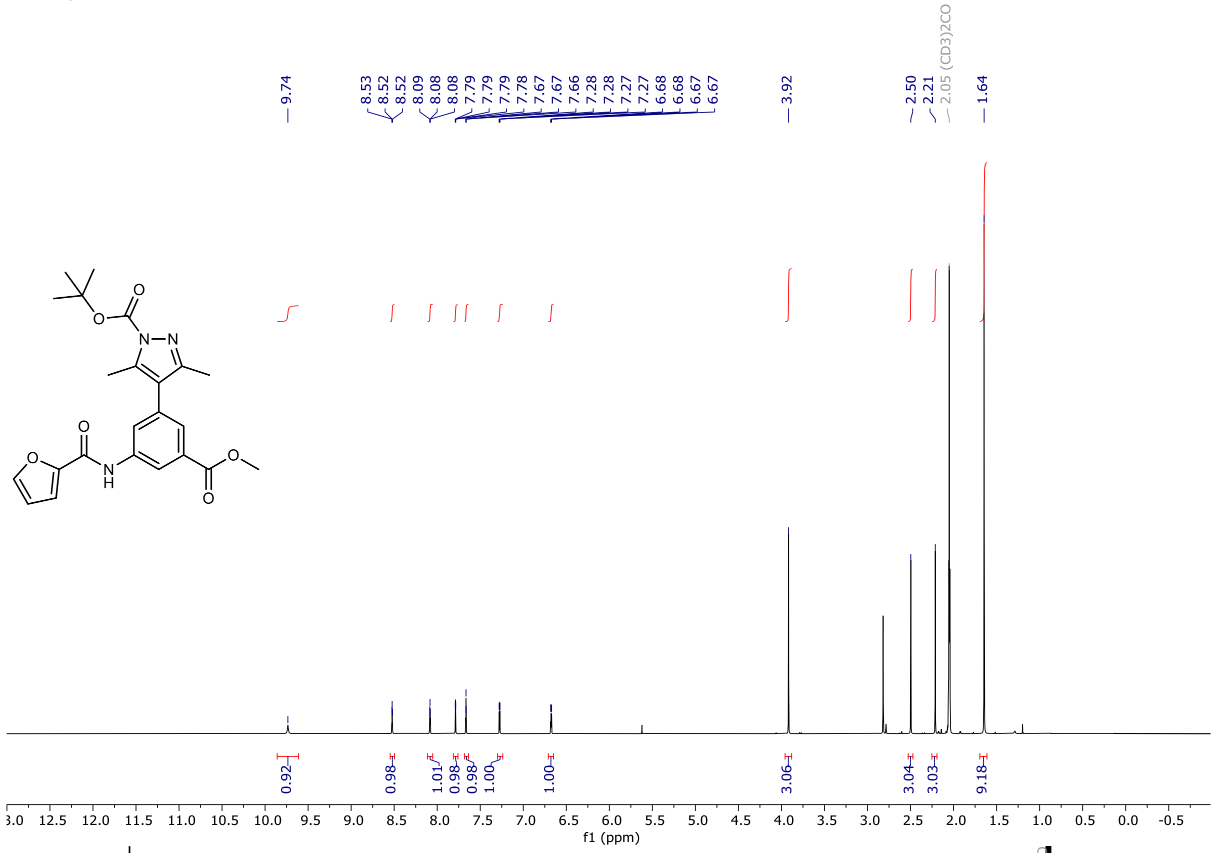


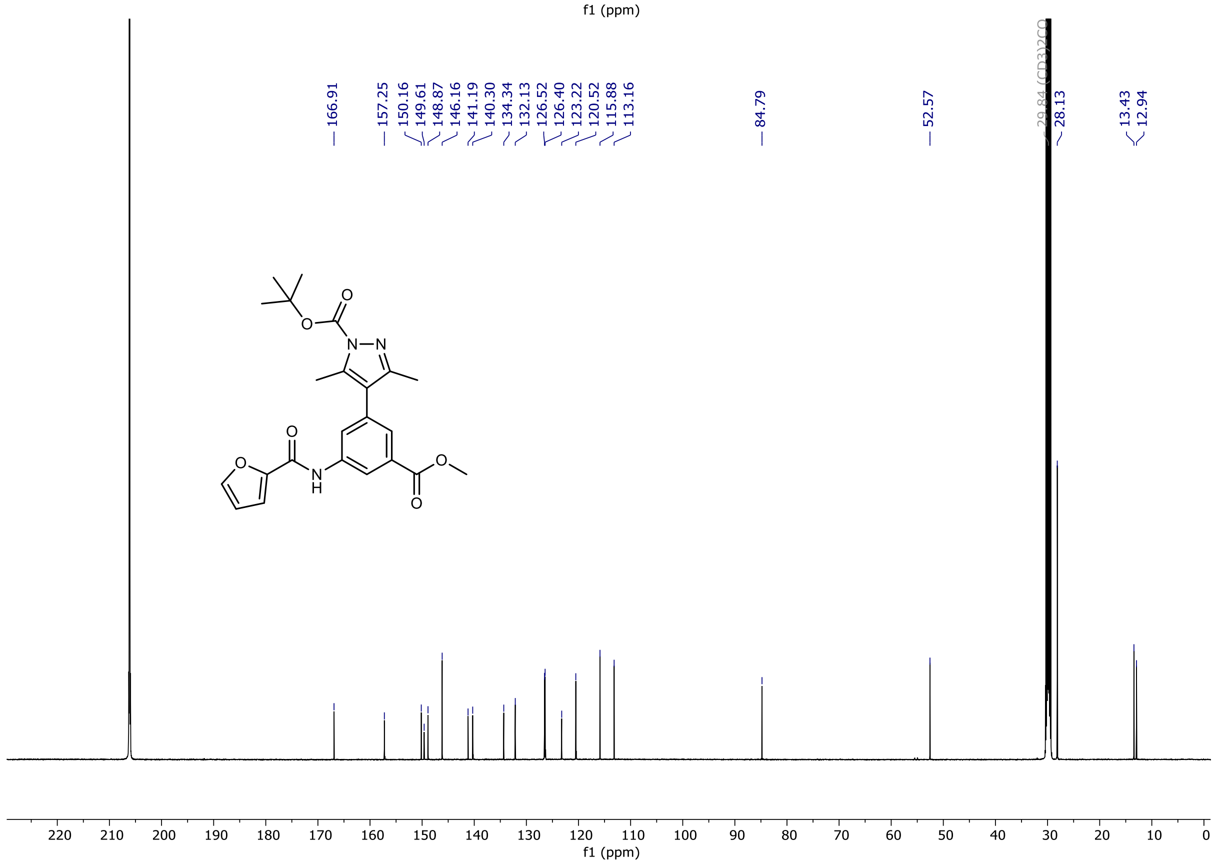

**Compound S16** $^{1}$H NMR (400 MHz, DMSO-*d6*) and $^{13}$C NMR (101 MHz, DMSO-$d_6$)

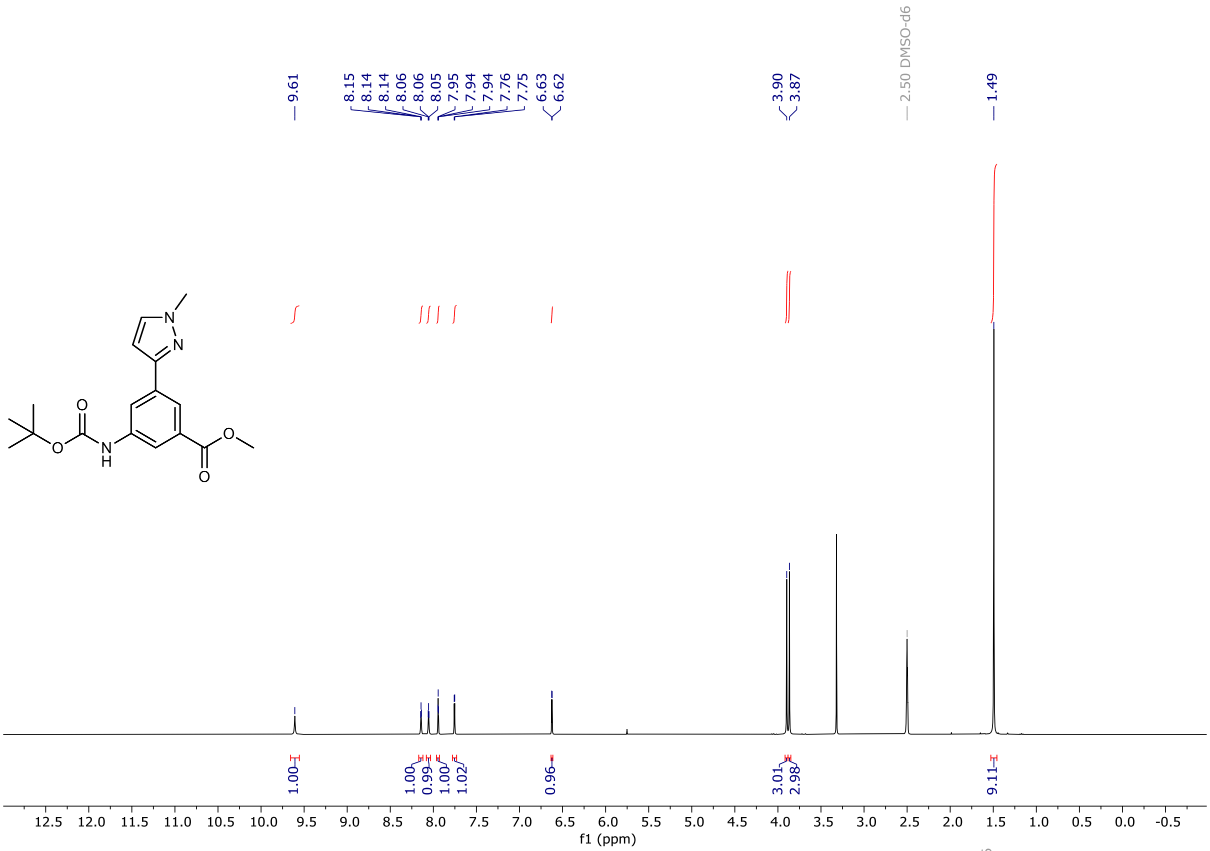


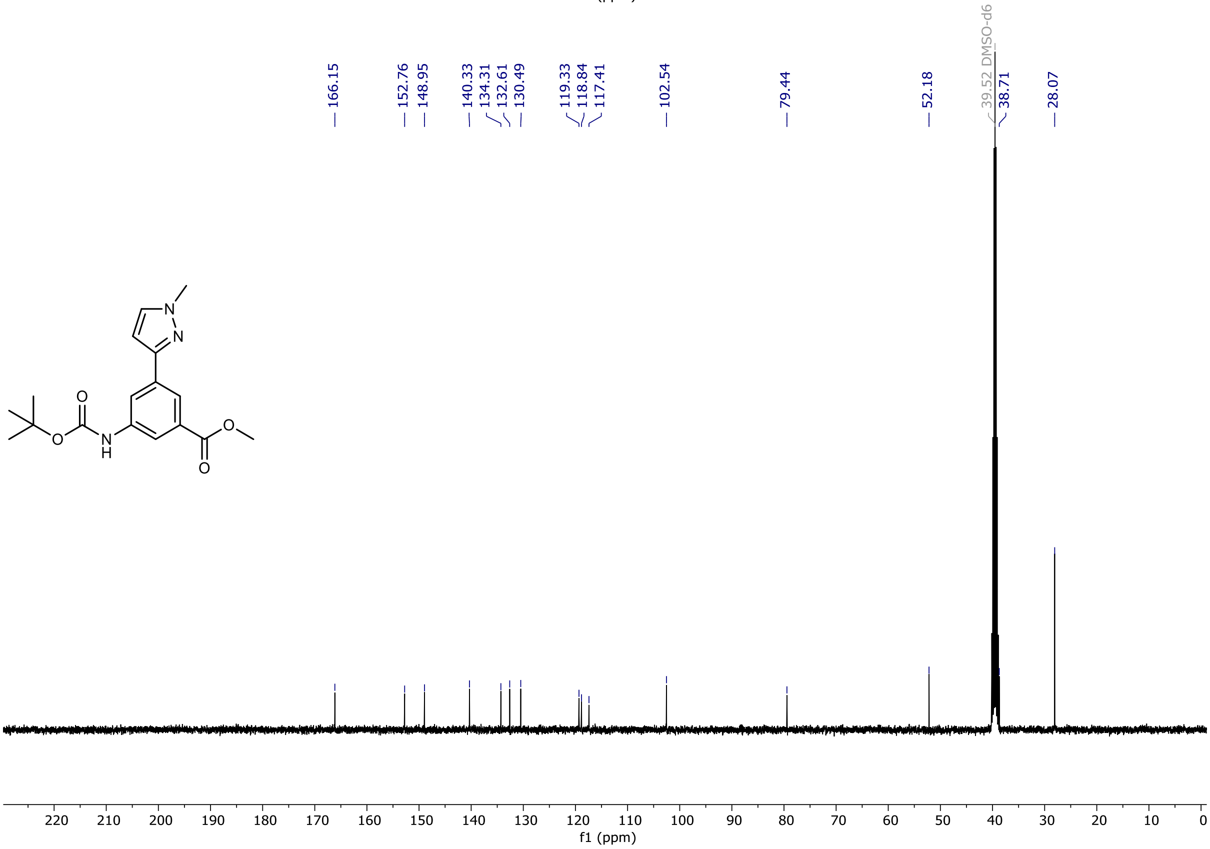

**Compound S17** $^{1}H$ NMR (400 MHz, $CDCl_3$) and $^{13}C$ NMR (101 MHz, $CDCl_3$)

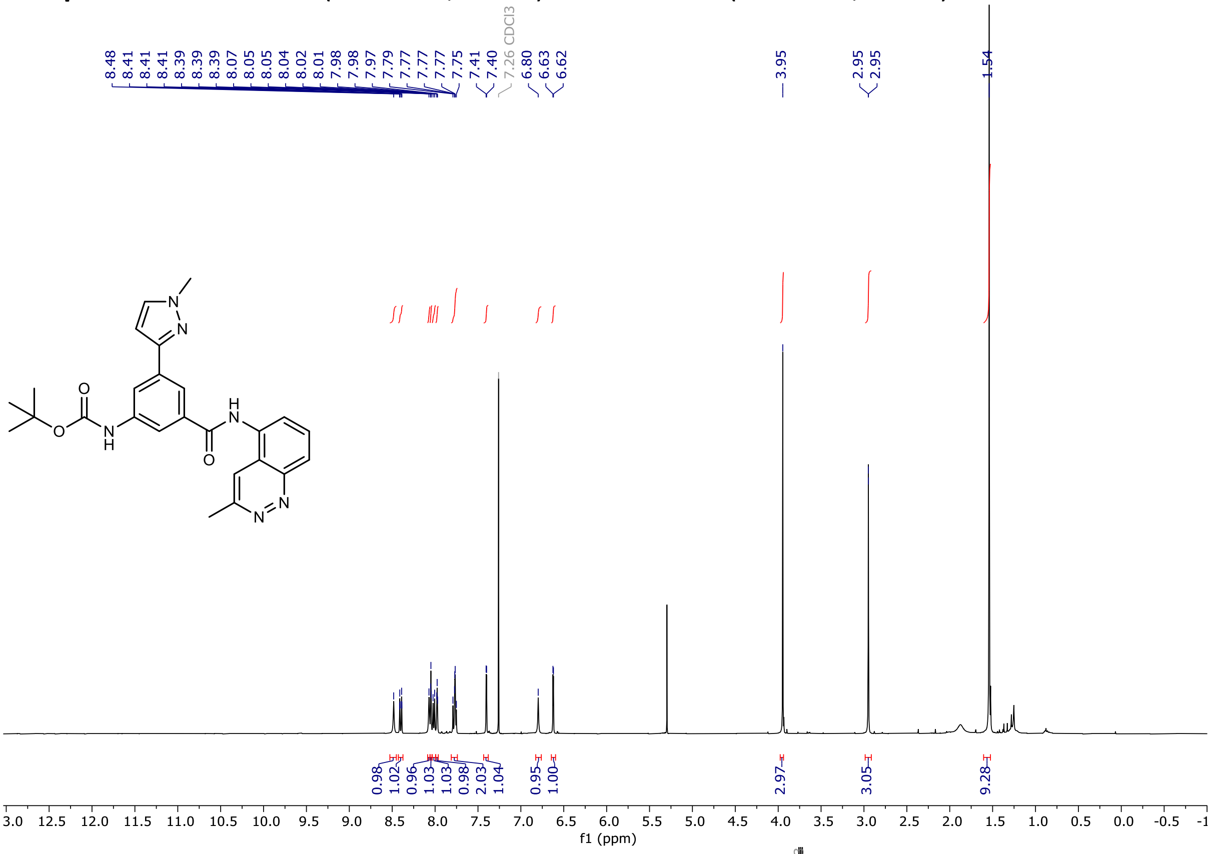


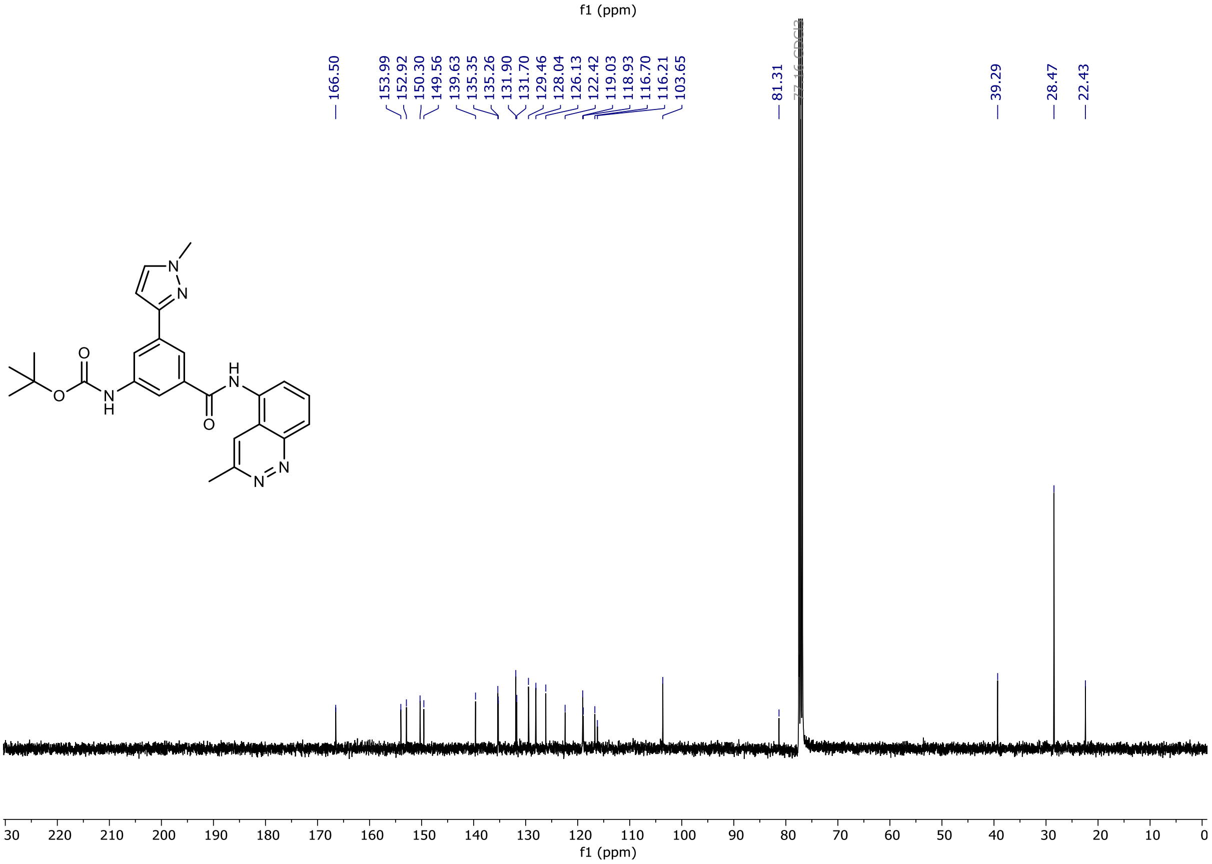

# References

(1) Quinn, E.; Wodicka, L.; Ciceri, P.; Pallares, G.; Pickle, E.; Torrey, A.; Floyd, M.; Hunt, J.; Treiber, D. Abstract 4238: BROMO Scan - a High Throughput, Quantitative Ligand Binding Platform Identifies Best-in-Class Bromodomain Inhibitors from a Screen of Mature Compounds Targeting Other Protein Classes. *Cancer Res* **2013**, *73* (8_Supplement), 4238–4238. https://doi.org/10.1158/1538-7445.AM2013-4238.

(2) Fabian, M. A.; Biggs, W. H.; Treiber, D. K.; Atteridge, C. E.; Azimioara, M. D.; Benedetti, M. G.; Carter, T. A.; Ciceri, P.; Edeen, P. T.; Floyd, M.; Ford, J. M.; Galvin, M.; Gerlach, J. L.; Grotzfeld, R. M.; Herrgard, S.; Insko, D. E.; Insko, M. A.; Lai, A. G.; Lélias, J.-M.; Mehta, S. A.; Milanov, Z. V.; Velasco, A. M.; Wodicka, L. M.; Patel, H. K.; Zarrinkar, P. P.; Lockhart, D. J. A Small Molecule–Kinase Interaction Map for Clinical Kinase Inhibitors. *Nat Biotechnol* **2005**, *23* (3), 329–336. https://doi.org/10.1038/nbt1068.

(3) Hay, D. A.; Fedorov, O.; Martin, S.; Singleton, D. C.; Tallant, C.; Wells, C.; Picaud, S.; Philpott, M.; Monteiro, O. P.; Rogers, C. M.; Conway, S. J.; Rooney, T. P. C.; Tumber, A.; Yapp, C.; Filippakopoulos, P.; Bunnage, M. E.; Müller, S.; Knapp, S.; Schofield, C. J.; Brennan, P. E. Discovery and Optimization of Small-Molecule Ligands for the CBP/P300 Bromodomains. *J Am Chem Soc* **2014**, *136* (26), 9308–9319. https://doi.org/10.1021/ja412434f.

(4) Picaud, S.; Fedorov, O.; Thanasopoulou, A.; Leonards, K.; Jones, K.; Meier, J.; Olzscha, H.; Monteiro, O.; Martin, S.; Philpott, M.; Tumber, A.; Filippakopoulos, P.; Yapp, C.; Wells, C.; Che, K. H.; Bannister, A.; Robson, S.; Kumar, U.; Parr, N.; Lee, K.; Lugo, D.; Jeffrey, P.; Taylor, S.; Vecellio, M. L.; Bountra, C.; Brennan, P. E.; O'Mahony, A.; Velichko, S.; Müller, S.; Hay, D.; Daniels, D. L.; Urh, M.; La Thangue, N. B.; Kouzarides, T.; Prinjha, R.; Schwaller, J.; Knapp, S. Generation of a Selective Small Molecule Inhibitor of the CBP/P300 Bromodomain for Leukemia Therapy. *Cancer Res* **2015**, *75* (23), 5106–5119. https://doi.org/10.1158/0008-5472.CAN-15-0236.

(5) Popp, T. A.; Tallant, C.; Rogers, C.; Fedorov, O.; Brennan, P. E.; Müller, S.; Knapp, S.; Bracher, F. Development of Selective CBP/P300 Benzoxazepine Bromodomain Inhibitors. *J Med Chem* **2016**, *59* (19), 8889–8912. https://doi.org/10.1021/acs.jmedchem.6b00774.

(6) Taylor, A. M.; Côté, A.; Hewitt, M. C.; Pastor, R.; Leblanc, Y.; Nasveschuk, C. G.; Romero, F. A.; Crawford, T. D.; Cantone, N.; Jayaram, H.; Setser, J.; Murray, J.; Beresini, M. H.; de Leon Boenig, G.; Chen, Z.; Conery, A. R.; Cummings, R. T.; Dakin, L. A.; Flynn, E. M.; Huang, O. W.; Kaufman, S.; Keller, P. J.; Kiefer, J. R.; Lai, T.; Li, Y.; Liao, J.; Liu, W.; Lu, H.; Pardo, E.; Tsui, V.; Wang, J.; Wang, Y.; Xu, Z.; Yan, F.; Yu, D.; Zawadzke, L.; Zhu, X.; Zhu, X.; Sims, R. J.; Cochran, A. G.; Bellon, S.; Audia, J. E.; Magnuson, S.; Albrecht, B. K. Fragment-Based Discovery of a Selective and Cell-Active Benzodiazepinone CBP/EP300 Bromodomain Inhibitor (CPI-637). *ACS Med Chem Lett* **2016**, *7* (5), 531–536. https://doi.org/10.1021/acsmedchemlett.6b00075.

(7) Crawford, T. D.; Romero, F. A.; Lai, K. W.; Tsui, V.; Taylor, A. M.; de Leon Boenig, G.; Noland, C. L.; Murray, J.; Ly, J.; Choo, E. F.; Hunsaker, T. L.; Chan, E. W.; Merchant, M.; Kharbanda, S.; Gascoigne, K. E.; Kaufman, S.; Beresini, M. H.; Liao, J.; Liu, W.; Chen, K. X.; Chen, Z.; Conery, A. R.; Côté, A.; Jayaram, H.; Jiang, Y.; Kiefer, J. R.; Kleinheinz, T.; Li, Y.; Maher, J.; Pardo, E.; Poy, F.; Spillane, K. L.; Wang, F.; Wang, J.; Wei, X.; Xu, Z.; Xu, Z.; Yen, I.; Zawadzke, L.; Zhu, X.; Bellon, S.; Cummings, R.; Cochran, A. G.; Albrecht, B. K.; Magnuson, S. Discovery of a Potent and Selective in Vivo Probe (GNE-272) for the Bromodomains of CBP/EP300. *J Med Chem* **2016**, *59* (23), 10549–10563. https://doi.org/10.1021/acs.jmedchem.6b01022.

(8) Romero, F. A.; Murray, J.; Lai, K. W.; Tsui, V.; Albrecht, B. K.; An, L.; Beresini, M. H.; De Leon Boenig, G.; Bronner, S. M.; Chan, E. W.; Chen, K. X.; Chen, Z.; Choo, E. F.; Clagg, K.; Clark, K.; Crawford, T. D.; Cyr, P.; De Almeida Nagata, D.; Gascoigne, K. E.; Grogan, J. L.; Hatzivassiliou, G.; Huang, W.; Hunsaker, T. L.; Kaufman, S.; Koenig, S. G.; Li, R.; Li, Y.; Liang, X.; Liao, J.; Liu, W.; Ly, J.; Maher, J.; Masui, C.; Merchant, M.; Ran, Y.; Taylor, A. M.; Wai, J.; Wang, F.; Wei, X.; Yu, D.; Zhu, B. Y.; Zhu, X.; Magnuson, S. GNE-781, A Highly Advanced Potent and Selective Bromodomain Inhibitor of Cyclic Adenosine Monophosphate Response Element Binding Protein, Binding Protein (CBP). *J Med Chem* **2017**, *60* (22), 9162–9183. https://doi.org/10.1021/acs.jmedchem.7b00796.

(9) Pegg, N.; Brooks, N.; Worthington, J.; Young, B.; Prosser, A.; Lane, J.; Taddei, D.; Brown, R.; Harbottle, G.; Shannon, J.; Paoletta, S.; Knudsen, K. E. Characterisation of CCS1477: A Novel Small Molecule Inhibitor of P300/CBP for the Treatment of Castration Resistant Prostate Cancer. *Journal of Clinical Oncology* **2017**, *35* (15_suppl), 11590–11590. https://doi.org/10.1200/JCO.2017.35.15_suppl.11590.

(10) Brooks, N.; Pegg, N.; Worthington, J.; Young, B.; Prosser, A.; Lane, J.; Taddei, D.; Schiewer, M. J.; Gordon, N.; Knudsen, K. E. A Novel Small Molecule Inhibitor of P300/CBP for the Treatment of Castration-Resistant Prostate Cancer: Preclinical Evaluation. *Journal of Clinical Oncology* **2017**, *35* (6_suppl), 168–168. https://doi.org/10.1200/JCO.2017.35.6_suppl.168.

(11) Batiste, L.; Unzue, A.; Dolbois, A.; Hassler, F.; Wang, X.; Deerain, N.; Zhu, J.; Spiliotopoulos, D.; Nevado, C.; Caflisch, A. Chemical Space Expansion of Bromodomain Ligands Guided by in Silico Virtual Couplings (AutoCouple). *ACS Cent Sci* **2018**, *4* (2), 180–188. https://doi.org/10.1021/acscentsci.7b00401.

(12) Muthengi, A.; Wimalasena, V. K.; Yosief, H. O.; Bikowitz, M. J.; Sigua, L. H.; Wang, T.; Li, D.; Gaieb, Z.; Dhawan, G.; Liu, S.; Erickson, J.; Amaro, R. E.; Schönbrunn, E.; Qi, J.; Zhang, W. Development of Dimethylisoxazole-Attached Imidazo[1,2-a]Pyridines as Potent and Selective CBP/P300 Inhibitors. *J Med Chem* **2021**, *64* (9), 5787–5801. https://doi.org/10.1021/acs.jmedchem.0c02232.

(13) Shoemaker, R. H. The NCI60 Human Tumour Cell Line Anticancer Drug Screen. *Nat Rev Cancer* **2006**, *6* (10), 813–823. https://doi.org/10.1038/nrc1951.

(14) Kabsch, W. XDS. *Acta Crystallogr D Biol Crystallogr* **2010**, *66* (Pt 2), 125–132. https://doi.org/10.1107/S0907444909047337.

(15) McCoy, A. J.; Grosse-Kunstleve, R. W.; Adams, P. D.; Winn, M. D.; Storoni, L. C.; Read, R. J. Phaser Crystallographic Software. *J Appl Crystallogr* **2007**, *40* (4), 658–674. https://doi.org/10.1107/S0021889807021206.

(16) Liebschner, D.; Afonine, P. V.; Baker, M. L.; Bunkoczi, G.; Chen, V. B.; Croll, T. I.; Hintze, B.; Hung, L. W.; Jain, S.; McCoy, A. J.; Moriarty, N. W.; Oeffner, R. D.; Poon, B. K.; Prisant, M. G.; Read, R. J.; Richardson, J. S.; Richardson, D. C.; Sammito, M. D.; Sobolev, O. V.; Stockwell, D. H.; Terwilliger, T. C.; Urzhumtsev, A. G.; Videau, L. L.; Williams, C. J.; Adams, P. D. Macromolecular Structure Determination Using X-Rays, Neutrons and Electrons: Recent Developments in Phenix. *Acta Crystallogr D Struct Biol* **2019**, *75* (10), 861–877. https://doi.org/10.1107/S2059798319011471.

(17) Emsley, P.; Lohkamp, B.; Scott, W. G.; Cowtan, K. Features and Development of Coot. *Acta Crystallogr D Biol Crystallogr* **2010**, *66* (4), 486–501. https://doi.org/10.1107/S0907444910007493.

(18) Afonine, P. V.; Grosse-Kunstleve, R. W.; Echols, N.; Headd, J. J.; Moriarty, N. W.; Mustyakimov, M.; Terwilliger, T. C.; Urzhumtsev, A.; Zwart, P. H.; Adams, P. D. Towards Automated Crystallographic Structure Refinement with Phenix.Refine. *Acta Crystallogr D Biol Crystallogr* **2012**, *68* (4), 352–367. https://doi.org/10.1107/S0907444912001308.

(19) *Caco-2 Permeability - Evotec*. https://www.evotec.com/en/drug-permeability-and-transporters/caco-2-permeability (accessed 2025-04-08).

(20) *NCI-60 Screening Methodology*. Developmental Therapeutics Program (DTP). https://dtp.cancer.gov/discovery_development/nci-60/methodology.htm (accessed 2024-02-09).

(21) Mancuso, L.; Jürjens, G.; Hermane, J.; Harmrolfs, K.; Eichner, S.; Fohrer, J.; Collisi, W.; Sasse, F.; Kirschning, A. Bioreduction of Aryl Azides during Mutasynthesis of New Ansamitocins. *Org Lett* **2013**, *15* (17), 4442–4445. https://doi.org/10.1021/ol401989e.

(22) Ohsawa, A.; Kaihoh, T.; Itoh, T.; Okada, M.; Kawabata, C.; Yamaguchi, K.; Igeta, H. Reactions of N-Aminopyrazoles with Halogenating Reagents and Synthesis of 1,2,3-Triazines. *Chem Pharm Bull (Tokyo)* **1988**, *36* (10), 3838–3848. https://doi.org/10.1248/cpb.36.3838.

(23) Junker, A.; Balasubramanian, R.; Ciancetta, A.; Uliassi, E.; Kiselev, E.; Martiriggiano, C.; Trujillo, K.; Mtchedlidze, G.; Birdwell, L.; Brown, K. A.; Harden, T. K.; Jacobson, K. A. Structure-Based Design of 3-(4-Aryl-1H-1,2,3-Triazol-1-Yl)-Biphenyl Derivatives as P2Y14 Receptor Antagonists. *J Med Chem* **2016**, *59* (13), 6149–6168. https://doi.org/10.1021/acs.jmedchem.6b00044.

(24) Coterón, J. M.; Catterick, D.; Castro, J.; Chaparro, M. J.; Díaz, B.; Fernández, E.; Ferrer, S.; Gamo, F. J.; Gordo, M.; Gut, J.; De Las Heras, L.; Legac, J.; Marco, M.; Miguel, J.; Muñoz, V.; Porras, E.; De La Rosa, J. C.; Ruiz, J. R.; Sandoval, E.; Ventosa, P.; Rosenthal, P. J.; Fiandor, J. M. Falcipain Inhibitors: Optimization Studies of the 2-Pyrimidinecarbonitrile Lead Series. *J Med Chem* **2010**, *53* (16), 6129–6152. https://doi.org/10.1021/jm100556b.
